# The most detailed anatomical reconstruction of a Mesozoic coelacanth

**DOI:** 10.1371/journal.pone.0312026

**Published:** 2024-11-06

**Authors:** Luigi Manuelli, Jorge Mondéjar Fernández, Kathleen Dollman, Kudakwashe Jakata, Lionel Cavin

**Affiliations:** 1 Department of Genetics and Evolution, University of Geneva, Geneva, Switzerland; 2 Department of Geology and Paleontology, Natural History Museum of Geneva, Geneva, Switzerland; 3 Senckenberg Research Institute and Natural History Museum, Frankfurt am Main, Germany; 4 Muséum National d’Histoire Naturelle, Paris, France; 5 European Synchrotron Radiation Facility, Grenoble, France; New York Institute of Technology, UNITED STATES OF AMERICA

## Abstract

Although the split of coelacanths from other sarcopterygians is ancient, around 420 million years ago, the taxic diversity and the morphological disparity of the clade have remained relatively low, with a few exceptions. This supposedly slow evolutionary pace has earned the extant coelacanth *Latimeria* the nickname “living fossil”. This status generated much interest in both extinct and extant coelacanths leading to the production of numerous anatomical studies. However, detailed descriptions of extinct taxa are made difficult due to the quality of the fossil material which generally prevents fine comparisons with the extant *Latimeria*. Here we describe a new genus and species of coelacanth, *Graulia branchiodonta* gen. et sp. nov. from the Middle Triassic of Eastern France, based on microtomographical imaging using synchrotron radiation. Through exquisite 3D preservation of the specimens, we reconstructed the skeletal anatomy of this new species at an unprecedented level of detail for an extinct coelacanth, and barely achieved for the extant *Latimeria*. In particular, we identified a well-developed trilobed ossified lung whose function is still uncertain. The skeletal anatomy of *G*. *branchiodonta* displays the general Bauplan of Mesozoic coelacanths and a phylogenetic analysis resolved it as a basal Mawsoniidae, shedding light on the early diversification of one of the two major lineages of Mesozoic coelacanths. However, despite its exquisite preservation, *G*. *branchiodonta* carries a weak phylogenetic signal, highlighting that the sudden radiation of coelacanths in the Early and Middle Triassic makes it currently difficult to detect synapomorphies and resolve phylogenetic interrelationships among coelacanths in the aftermath of the great Permo-Triassic biodiversity crisis.

## Introduction

The Triassic period (between 252–201 million years ago) represents one of the most fascinating chapters in the long evolutionary history of coelacanths. The fossil record reveals that after the origin of the clade Actinistia in the Palaeozoic at the beginning of the Devonian period (more than 420 million years ago), the highest peak of coelacanth diversity occurred at the beginning of the Mesozoic during the early Triassic, with minor peaks in the late Devonian, early Carboniferous and late Jurassic [1, 2]. However, recent discoveries have revealed that not only the taxonomical diversity of coelacanths was at its highest during the early Mesozoic [3, 4], but also the morphological disparity, exemplified by unusual Triassic forms like *Rebellatrix divaricerca* from Canada [5], *Foreyia maxkuhni* and *Rieppelia heinzfurreri* from Switzerland [6, 7]. These exceptional departures from the ‘classic’ coelacanth body-plan, together with Palaeozoic forms like *Holopterygius nudus* from the Devonian [8] and *Allenypterus montanus* from the Carboniferous [2, 9], challenge the conventional portrayal of coelacanths as morphologically invariant and highlight our still approximate understanding of the evolution and paleoecology of these fishes.

Coelacanths display a unique set of features among sarcopterygians (lobe-finned fishes), many of them retained by the extant genus *Latimeria* (*L*. *chalumnae* Smith 1939 [10] from the South-eastern African coast and *L*. *menadoensis* Pouyaud, Wirjoatmodjo, Rachmatika, Tjakrawidjaja, Hadiaty, Hadie 1999 [11] and a possible third species [12] from Indonesia). Devonian taxa like *Miguashaia bureaui* [13] and *Gavinia syntrips* [14] are considered morphologically primitive coelacanths due to the retention of a generalized sarcopterygian arrangement of the bones of the skull roof and a heterocercal caudal fin. By contrast, anatomically modern coelacanths (i.e., taxa with two pairs of parietals, an elongated preorbital portion of the skull roof, and a trilobed caudal fin) [15] display derived features shared with *Latimeria* [2] that can be traced back to the Early Devonian. This apparent morphological evolutionary conservatism is considered one of the most remarkable traits of coelacanths among osteichthyans (bony fishes).

Following the mass extinction at the Permian-Triassic boundary, ca. 252 million years ago, coelacanths appear to have diversified during the Lower-Middle Triassic, leading to the exceptional diversity peak of the early Triassic [1–3, 16–19]. The specific and morphological diversity of coelacanths was particularly high in the Western Tethys during the Middle Triassic, especially between 242 and 240 million years ago. It is also during this time, that the two main Mesozoic coelacanth lineages diverged: the families Latimeriidae and Mawsoniidae [2, 7, 20], gathered within the clade Latimerioidei. Prior to the Mesozoic, coelacanths mainly inhabited marine environments, with only sparse occurrences in freshwaters from the Carboniferous and Permian (e.g., *Rhabdoderma* sp., *Synaptotylus newelli*, *Spermatodus pustulosus*) [2]. On the other hand, the Latimerioidei had representatives in both freshwater and marine environments, some of which attained gigantic sizes during the Mesozoic (e.g., the mawsoniids *Mawsonia* and *Axelrodichthys* and the latimeriid *Megalocoelacanthus*) [21–23]. The Latimeriidae is an exclusively marine lineage of coelacanths, including the extant *Latimeria* and other extinct representatives well known from the Jurassic and Cretaceous of Europe and North America [24], while the Mawsoniidae are common in brackish and freshwater environments from the Jurassic and Cretaceous of North and South America [20, 22, 24–27], Africa and Madagascar [20, 22, 28–30], Asia [31], and Europe [32–36]. Mawsoniids are assumed to have originated during the Late Triassic in North America, where they are known from freshwater environments. These occurrences include *Chinlea sorenseni* from the Upper Triassic [37, 38] and *Diplurus longicaudatus* from the Lower Jurassic [39, 40]. However, recently described mawsoniid fossil remains from the marine Upper Triassic (Rhaetian) of France [35] and Germany [36] confirm that mawsoniids already inhabited marine environments by the end of the Triassic in Europe.

Here we describe a new marine coelacanth from the Middle Triassic of Central Europe, represented by two specimens from France. A detailed microtomographical study using synchrotron radiation has revealed in exquisite detail the entire skeleton, comprising the skull, fins, lung, and the vertebral column. This new species represents one of the oldest mawsoniid coelacanths and constitutes the most completely preserved Mesozoic coelacanth to date, furnishing key information on the anatomy and interrelationships of these iconic fishes during the aftermath of the great Permian-Triassic mass extinction.

## Results

### Systematic paleontology

Class OSTEICHTHYES Huxley, 1880

Subclass SARCOPTERYGII Romer, 1955

Infraclass ACTINISTIA Cope, 1891

Order COELACANTHIFORMES Huxley, 1861

Suborder LATIMERIOIDEI Schultze, 1993

Family MAWSONIIDAE Schultze, 1993

*Graulia branchiodonta* gen. et sp. nov.

urn:lsid:zoobank.org:act:F0F7DD30-CF66-4F50-A83F-C7337A6A8BB3

urn:lsid:zoobank.org:act:4C892CE0-6606-4D2C-9F53-FC24D3521DD4

#### Holotype

**Fig 1:**
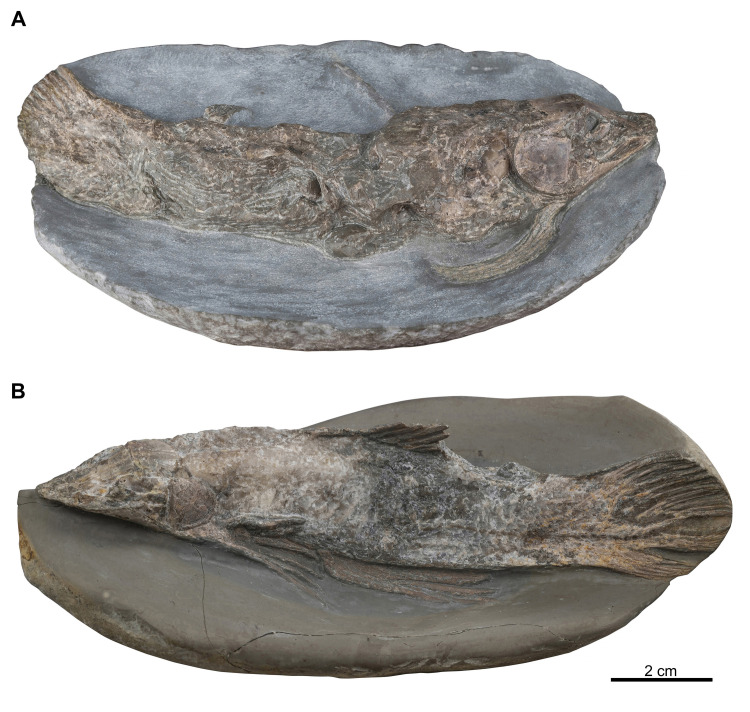
*Graulia branchiodonta* gen. et sp. nov. Photographs of the specimens in laterodorsal view. (A) MHNG GEPI V5787, holotype. (B) MHNG GEPI V5788.

MHNG GEPI V5787, complete specimen (Fig 1A).

#### Etymology

The genus name *Graulia* refers to the Graoully, Graouli or Graully, a mythical dragon from the folklore of Lorraine, the region of France where the specimens were found. The species name *branchiodonta*, from the greek βράγχια “gills” and ὀδούς, ὀδόντος “tooth” refers to the large teeth found on the ceratobranchials.

#### Diagnosis

Mawsoniid coelacanth characterized by the following association of characters: anteromedial process of the posterior parietal present; one pair of lateral extrascapulars; few pores at the sutural contact with bones enclosing the supraorbital sensory canal.; the presence of anterior branches of the supratemporal commissure; a preoperculum with an anterior blade-like portion; a simple anterior end of the lachrymojugal; the infraorbital sensory canal running at the anterior margin of the postorbital; long teeth on the ceratobranchial tooth plates; denticles on the rays of the first dorsal fin and on the first rays of the dorsal lobe of the caudal fin; tri-lobed unpaired lung; scales ornamented with pointed small tubercles. Only known species, same diagnosis as for the genus.

#### Locality

Sarraltroff, 57400 Moselle, Grand Est, France.

#### Age

Middle Triassic Muschelkalk, Calcaire à Cératites Formation, Praenodosus biozone (a ceratite ammonite) (Jean-Philippe et François-Xavier Blouet, personnal communication 2018). The praenodosus zone from France corresponds to the Tonhorizont epsilon from Germany, which belongs to the Hohenhole Formation from the Upper Muschelkalk, middle Ladinian (Fassanian, 242–237 Mya), early Middle Triassic. A detailed description of the geological setting will be published in a future article.

#### Referred material

MHNG GEPI V5787, complete specimen (Fig 1B).

#### Remarks

The anatomical descriptions are mainly based on the holotype MHNG GEPI V5787, the specimen with the best-preserved cranial anatomy, unless stated otherwise.

#### Description

The description is based on the anatomical information retrieved from synchrotron microCT scans of the holotype MHNG GEPI V5787 (Figs 2–17) and MHNG GEPI V5788 (Figs 2 and 18–28). Drawings of a restored specimen including information from all the available material are shown in Figs 29, 30.

**Fig 2:**
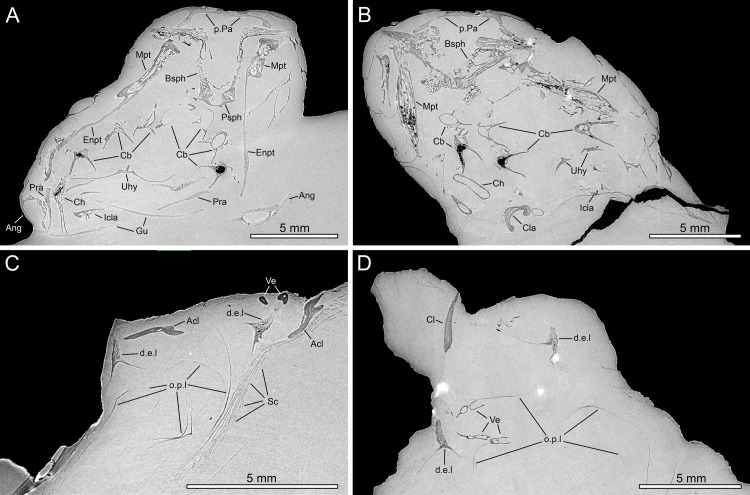
*Graulia branchiodonta* gen. et sp. nov., transversal sections of synchrotron microCT scans. (A) MHNG GEPI V5787, holotype, section through the skull at the level of the intracranial joint. (B) MHNG GEPI V5788, section through the skull at the level of the intracranial joint. (C) MHNG GEPI V5787, holotype, section posterior to the skull showing details of the ossified lung. (D) MHNG GEPI V5788, section posterior to the skull showing details of the ossified lung. A high-resolution version (1200 dpi) of this figure can be downloaded from Figshare (DOI: 10.6084/m9.figshare.26166943).

**Fig 3:**
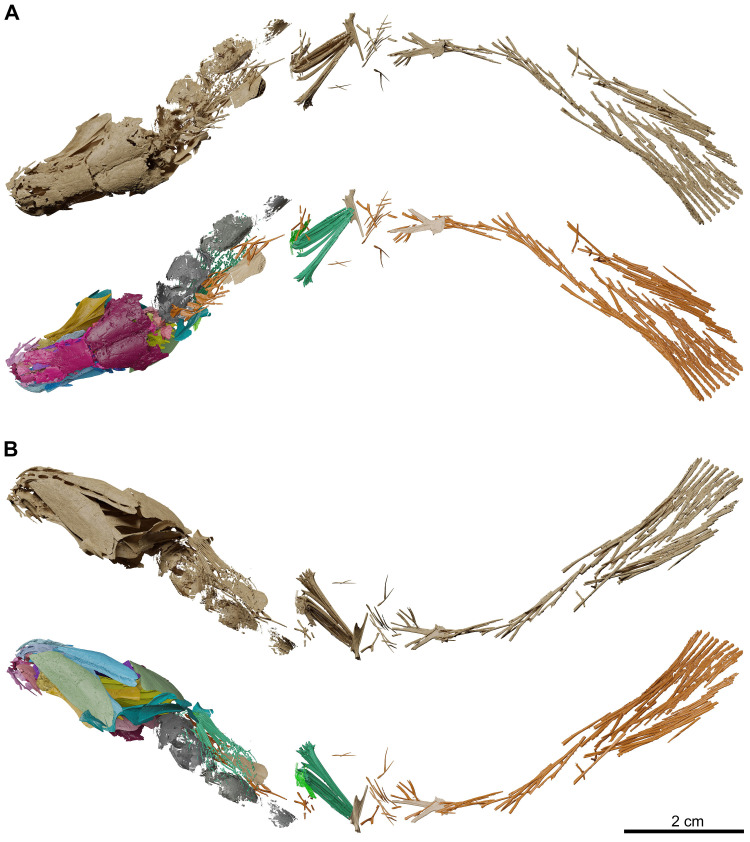
*Graulia branchiodonta* gen. et sp. nov., MHNG GEPI V5787, holotype, surface-rendered 3D models of the entire skeleton, obtained from synchrotron microCT. Several elements from the right side of the skull and from the axial skeleton have not been segmented and are not shown. (A) dorsal views of texturized (above) and colorized (below) skeleton. (B) ventral views of texturized (above) and colorized (below) skeleton. Color legend–Blue: cheek; Golden: hyoid arch; Green: scales and fin rays; Grey: lung; Light Blue: lower jaw; Light Green: operculogular series; Maroon: skull roof; Orange: vertebral column, caudal fin and basal plates of median fins; Pink: neurocranium; Purple: palate; Turquoise: pectoral girdle; Yellow: branchial arches. A high-resolution version (1200 dpi) of this figure can be downloaded from Figshare (DOI: 10.6084/m9.figshare.26166943).

**Fig 4:**
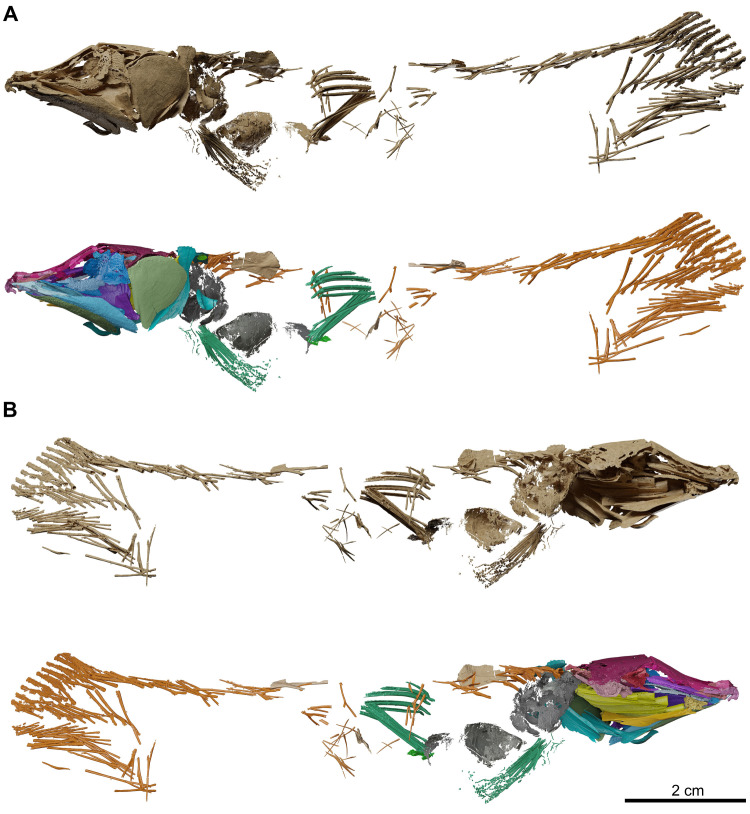
*Graulia branchiodonta* gen. et sp. nov., MHNG GEPI V5787, holotype, surface-rendered 3D models of the entire skeleton, obtained from synchrotron microCT. Several elements from the right side of the skull and from the axial skeleton have not been segmented and are not shown. (A) left lateral views of texturized (above) and colorized (below) skeleton. (B) right lateral views of texturized (above) and colorized (below) skeleton. Color legend as in Fig 3. A high-resolution version (1200 dpi) of this figure can be downloaded from Figshare (DOI: 10.6084/m9.figshare.26166943).

**Fig 5:**
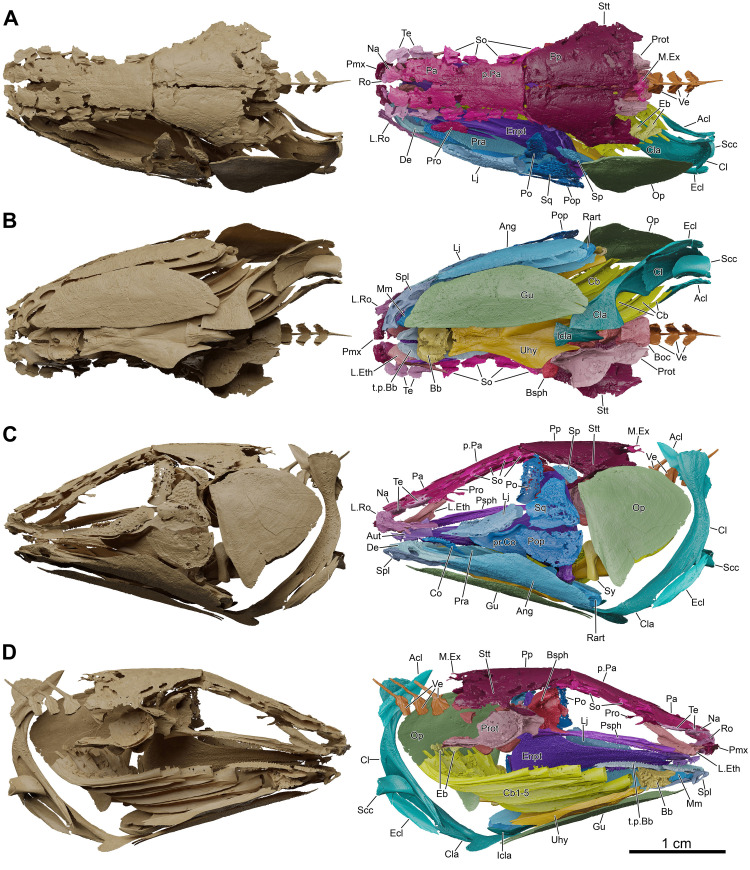
*Graulia branchiodonta* gen. et sp. nov., MHNG GEPI V5787, holotype, surface-rendered 3D models of the skull and pectoral girdle, obtained from synchrotron microCT. The skeletal elements have been repositioned in the 3D space using a 3D model of the skull of *Latimeria chalumnae* as a reference. Several elements from the right side of the skull have not been segmented and are not shown. The lateral rostral has been flipped from the right to the left side. (A) dorsal views of texturized (left) and colourized (right) skull. (B) ventral views of texturized (left) and colourized (right) skull. (C) left lateral views of texturized (left) and colourized (right) skull. (D) right lateral views of texturized (left) and colourized (right) skull. Color legend as in Fig 3. A high-resolution version (1200 dpi) of this figure can be downloaded from Figshare (DOI: 10.6084/m9.figshare.26166943).

**Fig 6:**
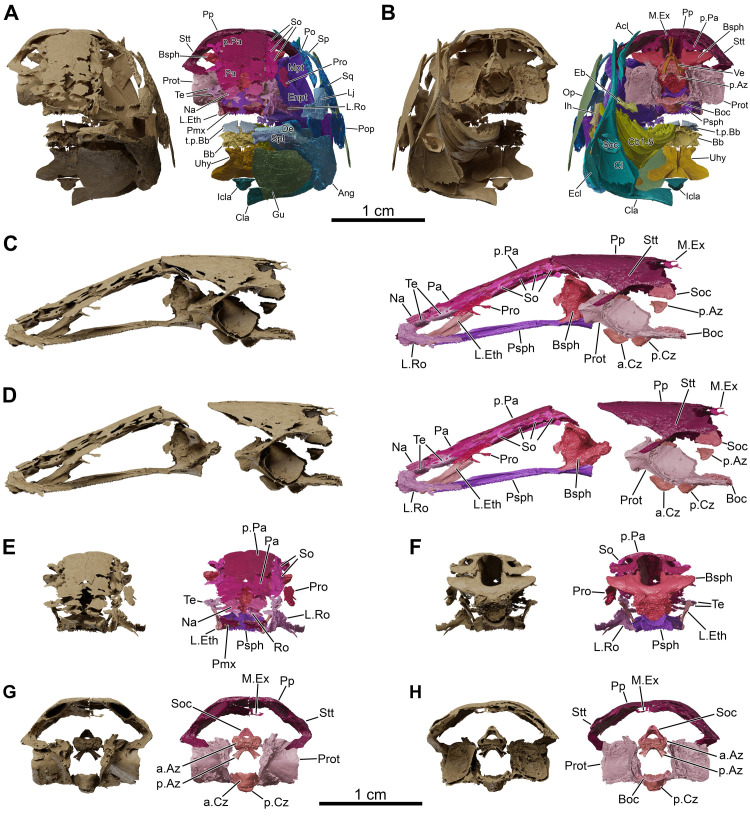
*Graulia branchiodonta* gen. et sp. nov., MHNG GEPI V5787, holotype, surface-rendered 3D models of the skull, pectoral girdle and isolated braincase, obtained from synchrotron microCT. The skeletal elements have been repositioned in the 3D space using a 3D model of the skull of *Latimeria chalumnae* as a reference. Several elements from the right side of the skull have not been segmented and are not shown. The lateral rostral has been flipped from the right to the left side. (A) anterior views of texturized (left) and colourized (right) skull. (B) posterior views of texturized (left) and colourized (right) skull. (C) left lateral views of texturized (left) and colorized (right) braincase. (D) left lateral views of texturized (left) and colorized (right) braincase disarticulated at the level of the intracranial joint. (E) anterior views of texturized (left) and colorized (right) ethmosphenoid portion of the braincase. (F) posterior views of texturized (left) and colorized (right) ethmosphenoid portion of the braincase. (G) anterior views of texturized (left) and colorized (right) otoccipital portion of the braincase. (H) posterior views of texturized (left) and colorized (right) otoccipital portion of the braincase. A high-resolution version (1200 dpi) of this figure can be downloaded from Figshare (DOI: 10.6084/m9.figshare.26166943).

**Fig 7:**
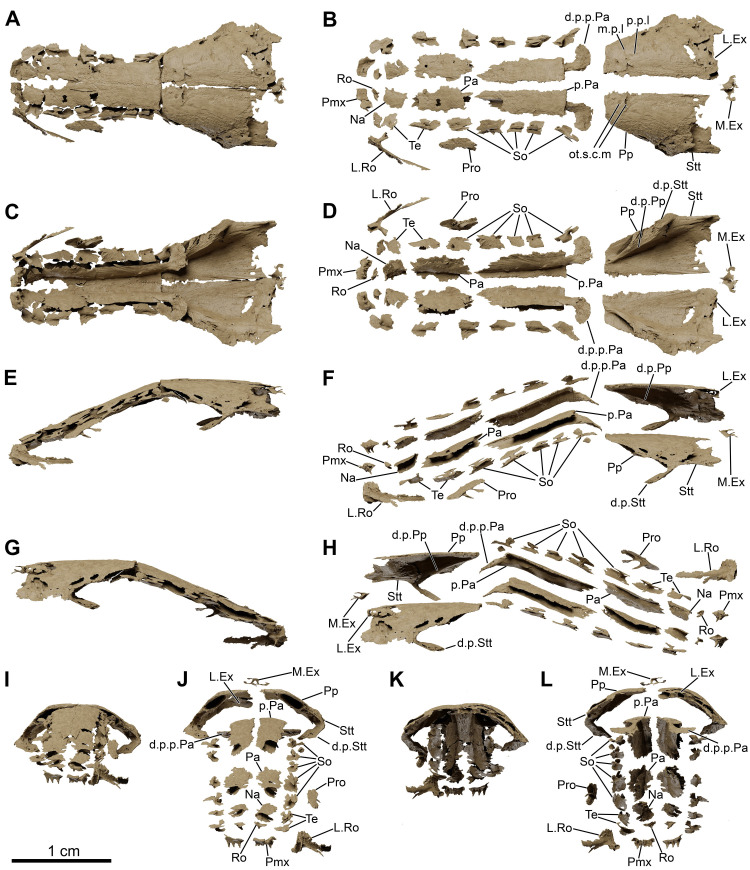
*Graulia branchiodonta* gen. et sp. nov., MHNG GEPI V5787, holotype, texturized surface-rendered 3D models of the skull roof, obtained from synchrotron microCT. The skeletal elements have been repositioned in the 3D space using a 3D model of the skull of *Latimeria chalumnae* as a reference. The lateral rostral has been flipped from the right to the left side. (A) dorsal view of the skull roof. (B) dorsal view of the skull roof with skeletal elements spaced apart. (C) ventral view of the skull roof. (D) ventral view of the skull roof with skeletal elements spaced apart. (E) left lateral view of the skull roof. (F) left lateral view of the skull roof with skeletal elements spaced apart. (G) right lateral view of the skull roof. (H) right lateral view of the skull roof with skeletal elements spaced apart. (I) anterior view of the skull roof. (J) anterior view of the skull roof with skeletal elements spaced apart. (K) posterior view of the skull roof. (L) posterior view of the skull roof with skeletal elements spaced apart. A high-resolution version (1200 dpi) of this figure can be downloaded from Figshare (DOI: 10.6084/m9.figshare.26166943).

**Fig 8:**
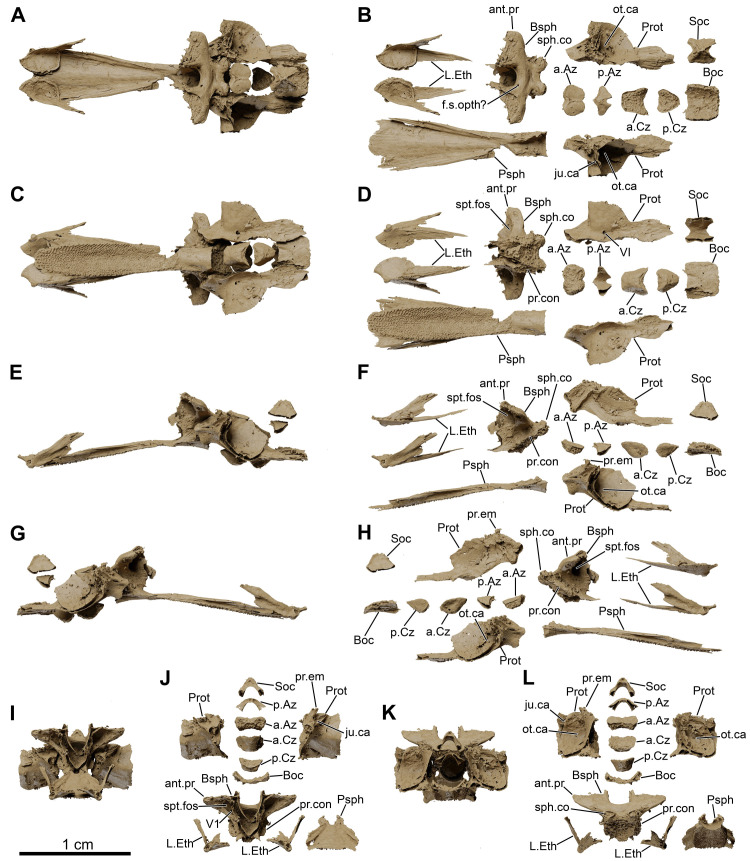
*Graulia branchiodonta* gen. et sp. nov., MHNG GEPI V5787, holotype, texturized surface-rendered 3D models of the neurocranium, obtained from synchrotron microCT. The skeletal elements have been repositioned in the 3D space using a 3D model of the skull of *Latimeria chalumnae* as a reference. (A) dorsal view of the neurocranium. (B) dorsal view of the neurocranium with skeletal elements spaced apart. (C) ventral view of the neurocranium. (D) ventral view of the neurocranium with skeletal elements spaced apart. (E) left lateral view of the neurocranium. (F) left lateral view of the neurocranium with skeletal elements spaced apart. (G) right lateral view of the neurocranium. (H) right lateral view of the neurocranium with skeletal elements spaced apart. (I) anterior view of the neurocranium. (J) anterior view of the neurocranium with skeletal elements spaced apart. (K) posterior view of the neurocranium. (L) posterior view of the neurocranium with skeletal elements spaced apart. A high-resolution version (1200 dpi) of this figure can be downloaded from Figshare (DOI: 10.6084/m9.figshare.26166943).

**Fig 9:**
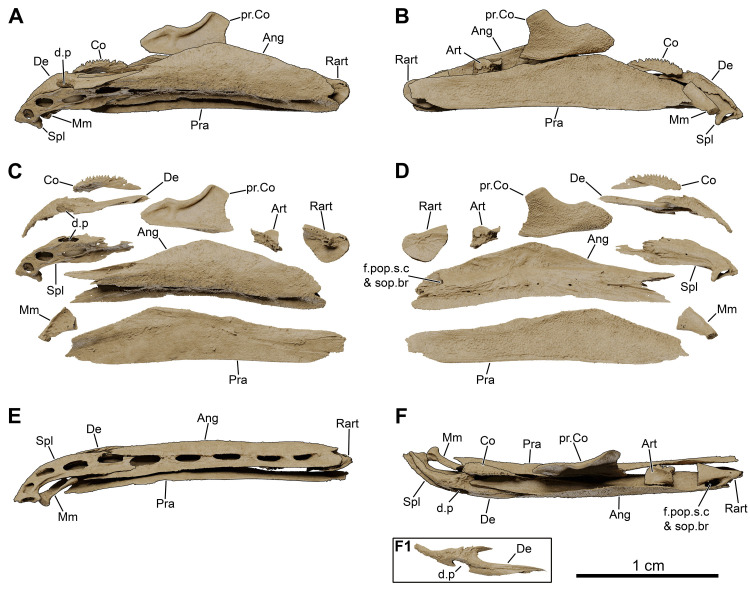
*Graulia branchiodonta* gen. et sp. nov., MHNG GEPI V5787, holotype, texturized surface-rendered 3D models of the lower jaw, obtained from synchrotron microCT. The first coronoids and dentary tooth plates are missing. The skeletal elements have been repositioned in the 3D space using a 3D model of the skull of *Latimeria chalumnae* as a reference. (A) lateroventral view of the lower jaw. (B) mediodorsal view of the lower jaw. (C) lateroventral view of the lower jaw with skeletal elements spaced apart. (D) mediodorsal view of the lower jaw with skeletal elements spaced apart. (E) medioventral view of the lower jaw. (F) laterodorsal view of the lower jaw. (F1) laterodorsal view of isolated dentary showing the hook process. A high-resolution version (1200 dpi) of this figure can be downloaded from Figshare (DOI: 10.6084/m9.figshare.26166943).

**Fig 10:**
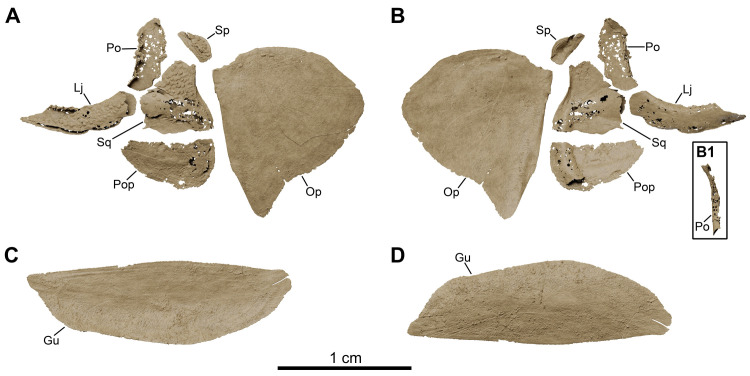
*Graulia branchiodonta* gen. et sp. nov., MHNG GEPI V5787, holotype, texturized surface-rendered 3D models of the cheek bones, operculars and gular plates, obtained from synchrotron microCT. The subopercular is missing here but it has been identified in MHNG GEPI V5788. The skeletal elements have been repositioned in the 3D space using a 3D model of the skull of *Latimeria chalumnae* as a reference. (A) lateral view of cheek bones and opercular. (B) medial view of cheek bones and opercular. (B1) anterior view of isolated postorbital. (C) dorsal view of gular plate. (D) ventral view of gular plate. A high-resolution version (1200 dpi) of this figure can be downloaded from Figshare (DOI: 10.6084/m9.figshare.26166943).

**Fig 11:**
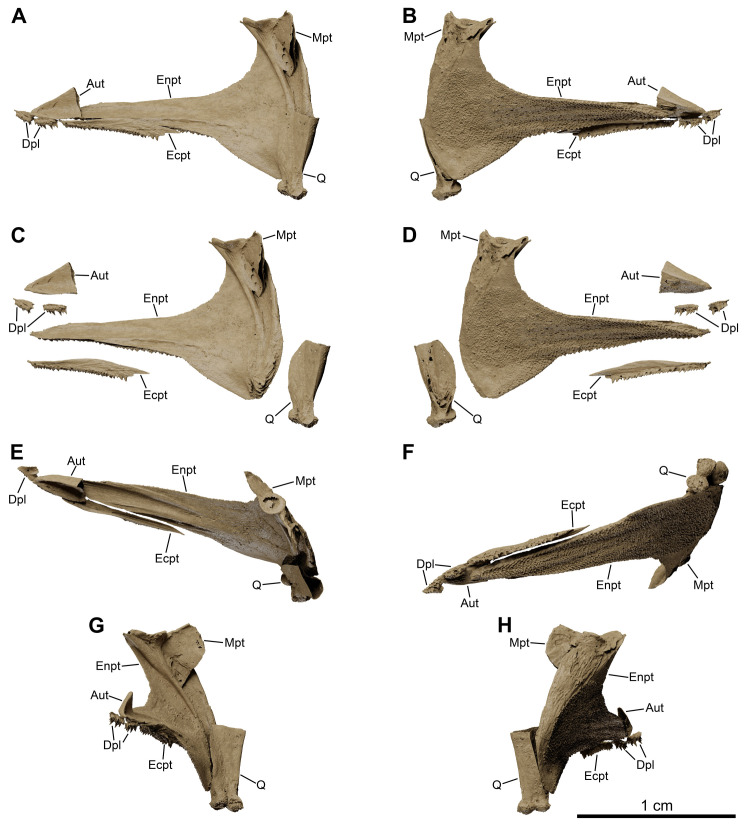
*Graulia branchiodonta* gen. et sp. nov., MHNG GEPI V5787, holotype, texturized surface-rendered 3D models of the palate, obtained from synchrotron microCT. The skeletal elements have been repositioned in the 3D space using a 3D model of the skull of *Latimeria chalumnae* as a reference. (A) lateral view of palate. (B) medial view of palate. (C) lateral view of palate with skeletal elements spaced apart. (D) medial view of palate with skeletal elements spaced apart. (E) dorsal view of palate. (F) ventral view of palate. (G) anterior view of palate. (H) posterior view of palate. A high-resolution version (1200 dpi) of this figure can be downloaded from Figshare (DOI: 10.6084/m9.figshare.26166943).

**Fig 12:**
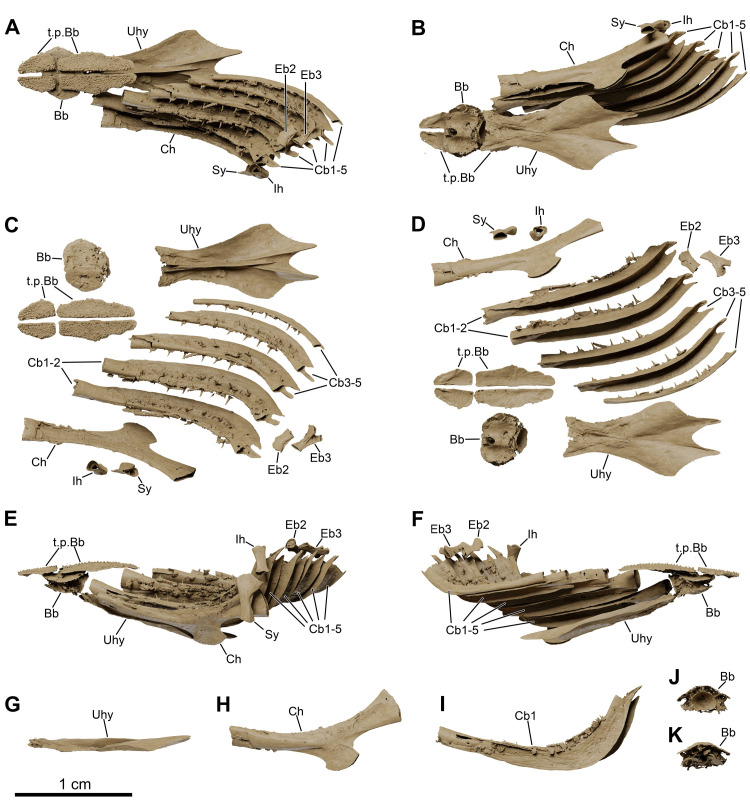
*Graulia branchiodonta* gen. et sp. nov., MHNG GEPI V5787, holotype, texturized surface-rendered 3D models of the hyobranchial skeleton, obtained from synchrotron microCT. The skeletal elements have been repositioned in the 3D space using a 3D model of the skull of *Latimeria chalumnae* as a reference. The gill and hyoid arches from the right side are not shown. (A) dorsal view of hyobranchial skeleton. (B) ventral view of hyobranchial skeleton. (C) dorsal view of hyobranchial skeleton with skeletal elements spaced apart. (D) ventral view of hyobranchial skeleton with skeletal elements spaced apart. (E) lateral view of hyobranchial skeleton. (F) medial view of hyobranchial skeleton. (G) lateral view of urohyal. (H) lateroventral view of ceratohyal. (I) lateroventral view of first ceratobranchial. (J) posterior view of basibranchial. (K) anterior view of basibranchial. A high-resolution version (1200 dpi) of this figure can be downloaded from Figshare (DOI: 10.6084/m9.figshare.26166943).

**Fig 13:**
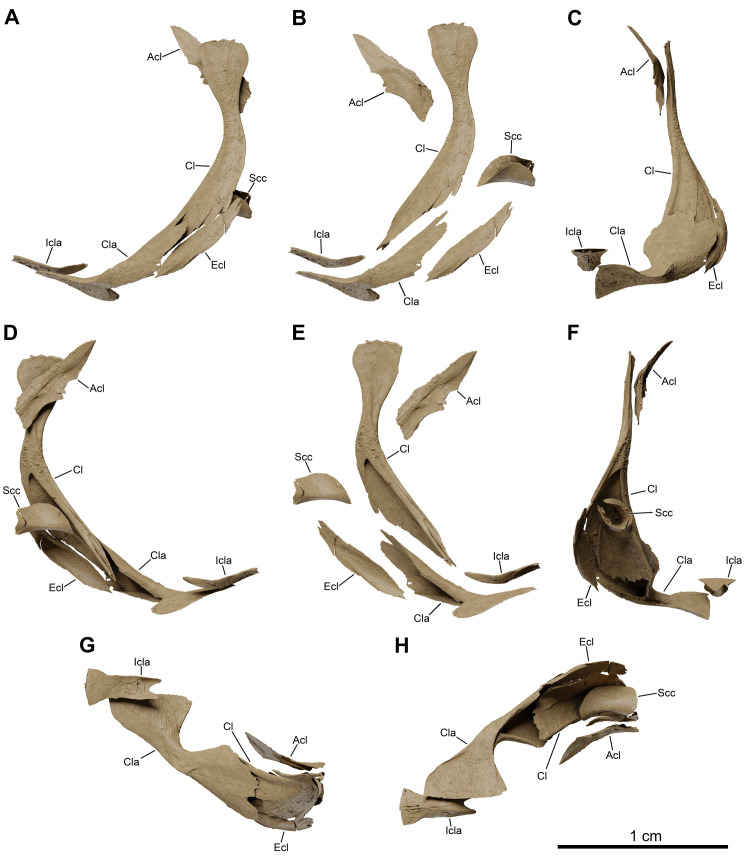
*Graulia branchiodonta* gen. et sp. nov., MHNG GEPI V5787, holotype, texturized surface-rendered 3D models of the pectoral girdle, obtained from synchrotron microCT. The skeletal elements have been repositioned in the 3D space using a 3D model of the pectoral girdle of *Latimeria chalumnae* as a reference. (A) lateral view of pectoral girdle. (B) lateral view of pectoral girdle with skeletal elements spaced apart. (C) anterior view of pectoral girdle. (D) medial view of pectoral girdle. (E) medial view of pectoral girdle with skeletal elements spaced apart. (F) posterior view of pectoral girdle. (G) dorsal view of pectoral girdle. (H) ventral view of pectoral girdle. A high-resolution version (1200 dpi) of this figure can be downloaded from Figshare (DOI: 10.6084/m9.figshare.26166943).

**Fig 14:**
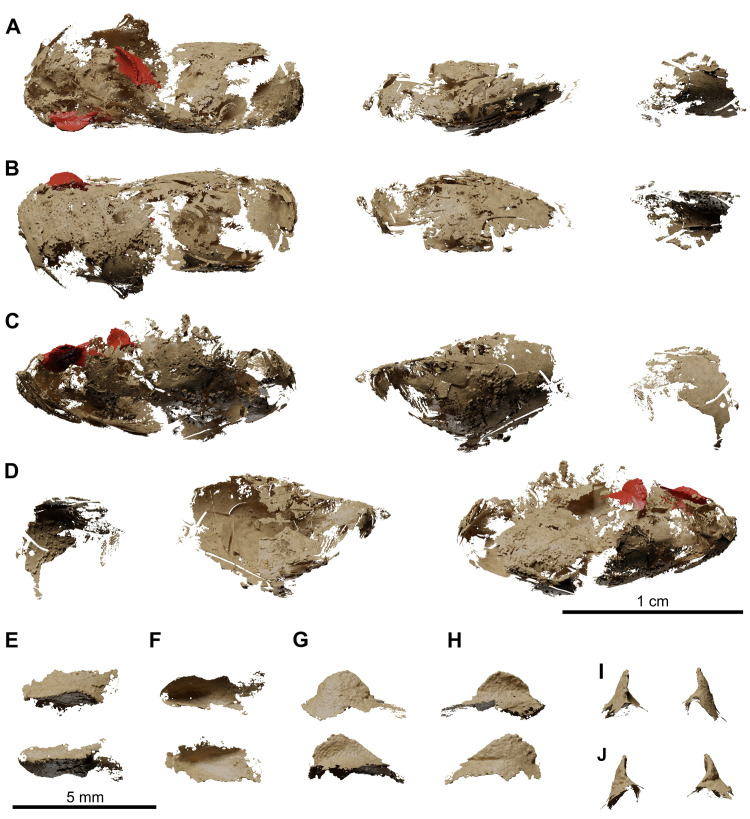
*Graulia branchiodonta* gen. et sp. nov., MHNG GEPI V5787, holotype, texturized surface-rendered 3D models of the lung, obtained from synchrotron microCT. The first lobe of the lung shows two dorsal crests colorized in red in A-D and with a bony texture in E-J. (A) dorsal view of the three lobes of the lung. (B) ventral view of the three lobes of the lung. (C) left lateral view of the three lobes of the lung. (D) right lateral view of the three lobes of the lung. (E) dorsal view of dorsal crests of the first lobe. (F) ventral view of dorsal crests of the first lobe. (G) left lateral view of dorsal crests of the first lobe. (H) right lateral view of dorsal crests of the first lobe. (I) anterior view of dorsal crests of the first lobe. (J) posterior view of dorsal crests of the first lobe. A high-resolution version (1200 dpi) of this figure can be downloaded from Figshare (DOI: 10.6084/m9.figshare.26166943).

**Fig 15:**
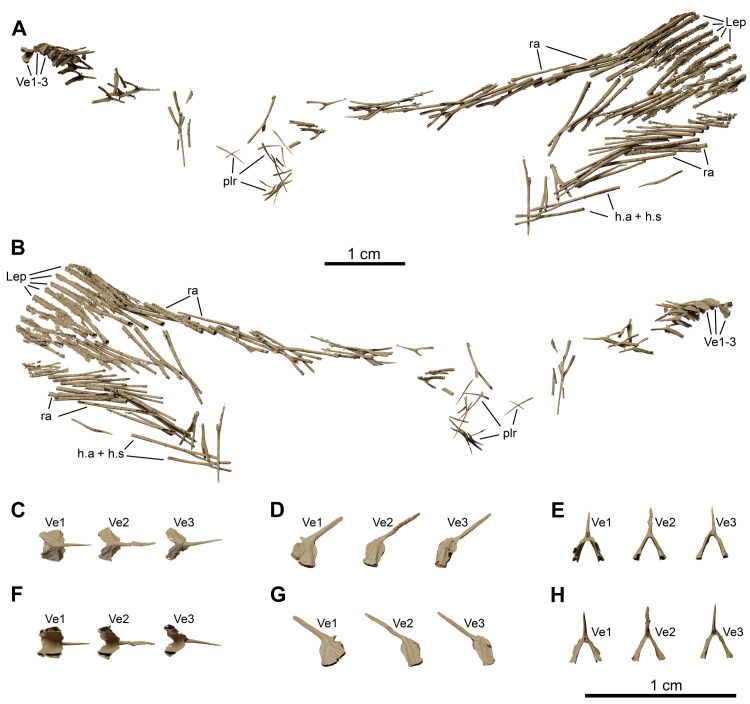
*Graulia branchiodonta* gen. et sp. nov., MHNG GEPI V5787, holotype, texturized surface-rendered 3D models of the postcranial axial skeleton and tail, obtained from synchrotron microCT. (A) left lateral view of postcranial axial skeleton and tail. (B) right lateral view of postcranial axial skeleton and tail. (C) dorsal view of the first three vertebrae. (D) left lateral view of first three vertebrae. (E) anterior view of first three vertebrae. (F) ventral view of first three vertebrae. (G) right lateral view of first three vertebrae. (H) posterior view of first three vertebrae. A high-resolution version (1200 dpi) of this figure can be downloaded from Figshare (DOI: 10.6084/m9.figshare.26166943).

**Fig 16:**
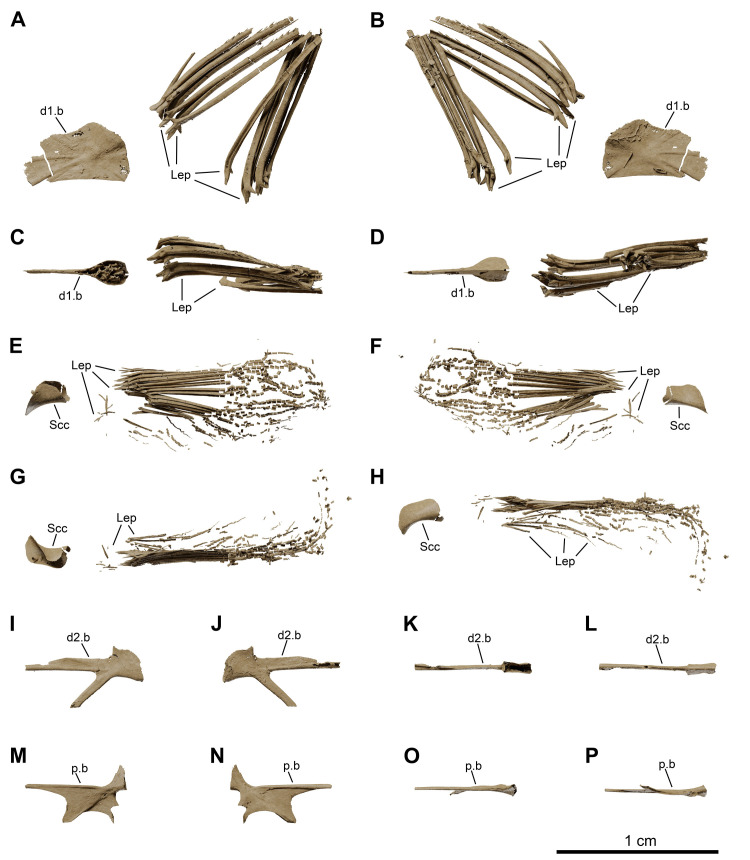
*Graulia branchiodonta* gen. et sp. nov., MHNG GEPI V5787, holotype, texturized surface-rendered 3D models of skeletal elements of the fins, obtained from synchrotron microCT. The skeletal elements have been repositioned in the 3D space using a 3D model of the pectoral girdle of *Latimeria chalumnae* as a reference. (A) left lateral view of first dorsal fin. (B) right lateral view of first dorsal fin. (C) dorsal view of first dorsal fin. (D) ventral view of first dorsal fin. (E) lateral view of left pectoral fin. (F) medial view of left pectoral fin. (G) dorsal view of left pectoral fin. (H) ventral view of left pectoral fin. (I) left lateral view of second dorsal fin. (J) right lateral view of second dorsal fin. (K) dorsal view of second dorsal fin. (L) ventral view of second dorsal fin. (M) dorsal view of pelvic fin. (N) ventral view of pelvic fin. (O) lateral view of pelvic fin. (P) medial view of pelvic fin. A high-resolution version (1200 dpi) of this figure can be downloaded from Figshare (DOI: 10.6084/m9.figshare.26166943).

**Fig 17:**
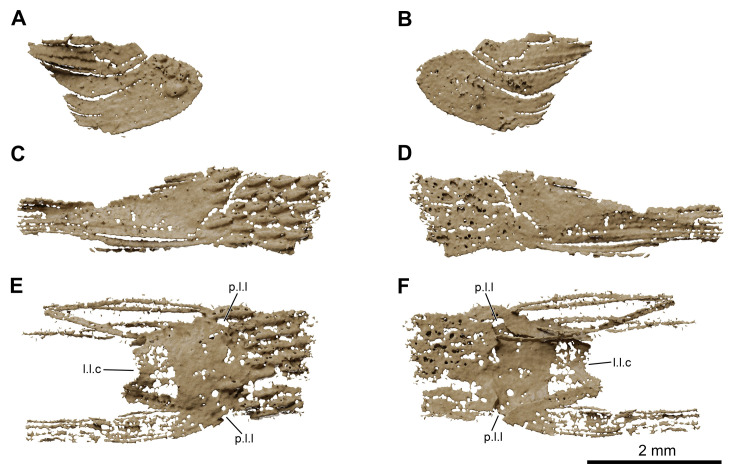
*Graulia branchiodonta* gen. et sp. nov., MHNG GEPI V5787, holotype, texturized surface-rendered 3D models of selected scales, obtained from synchrotron microCT. (A) lateral view of scale from the dorsal region next to the anocleithrum. (B) medial view of scale from the dorsal region found next to the anocleithrum. (C) lateral view of fragment of scale from the thoracical region close to the third lobe of the lung. (D) medial view of fragment of scale from the thoracical region close to the third lobe of the lung. (E) lateral view of damaged lateral line scale found close to the third lobe of the lung. (F) medial view of damaged lateral line scale found close to the third lobe of the lung. A high-resolution version (1200 dpi) of this figure can be downloaded from Figshare (DOI: 10.6084/m9.figshare.26166943).

**Fig 18:**
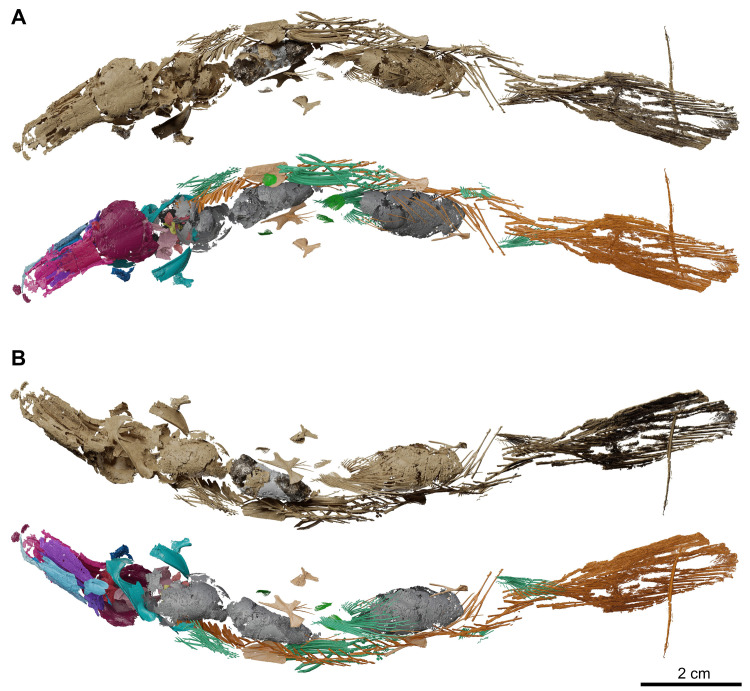
*Graulia branchiodonta* gen. et sp. nov., MHNG GEPI V5788, surface-rendered 3D models of the entire skeleton, obtained from synchrotron microCT. Several elements of the skull and the axial skeleton have not been segmented and are not shown. (A) dorsal views of texturized (above) and colorized (below) skeleton. (B) ventral views of texturized (above) and colorized (below) skeleton. In the texturized renders, parts of the lung are shaded as grey metal or white plastic to represent different densities. Color legend–Blue: cheek; Golden: hyoid arch; Green: scales and fin rays; Grey: lung; Light Blue: lower jaw; Light Green: operculogular series; Maroon: skull roof; Orange: vertebral column, caudal fin and basal plates of median fins; Pink: neurocranium; Purple: palate; Turquoise: pectoral girdle; Yellow: branchial arches. A high-resolution version (1200 dpi) of this figure can be downloaded from Figshare (DOI: 10.6084/m9.figshare.26166943).

**Fig 19:**
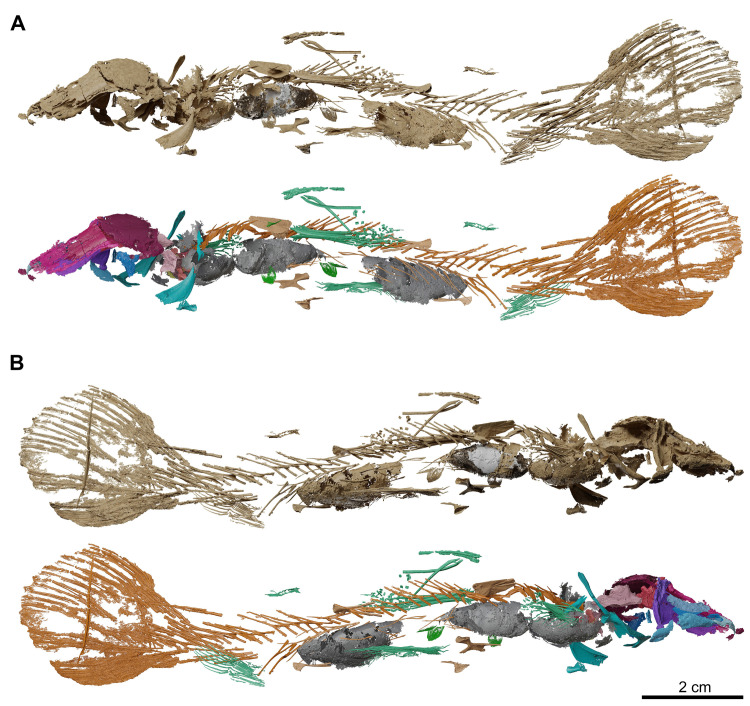
*Graulia branchiodonta* gen. et sp. nov., MHNG GEPI V5788, surface-rendered 3D models of the entire skeleton, obtained from synchrotron microCT. Several elements of the skull and the axial skeleton have not been segmented and are not shown. (A) left lateral views of texturized (above) and colorized (below) skeleton. (B) right lateral views of texturized (above) and colorized (below) skeleton. In the texturized renders, parts of the lung are shaded as grey metal or white plastic to represent different densities. Color legend as in Fig 18. A high-resolution version (1200 dpi) of this figure can be downloaded from Figshare (DOI: 10.6084/m9.figshare.26166943).

**Fig 20:**
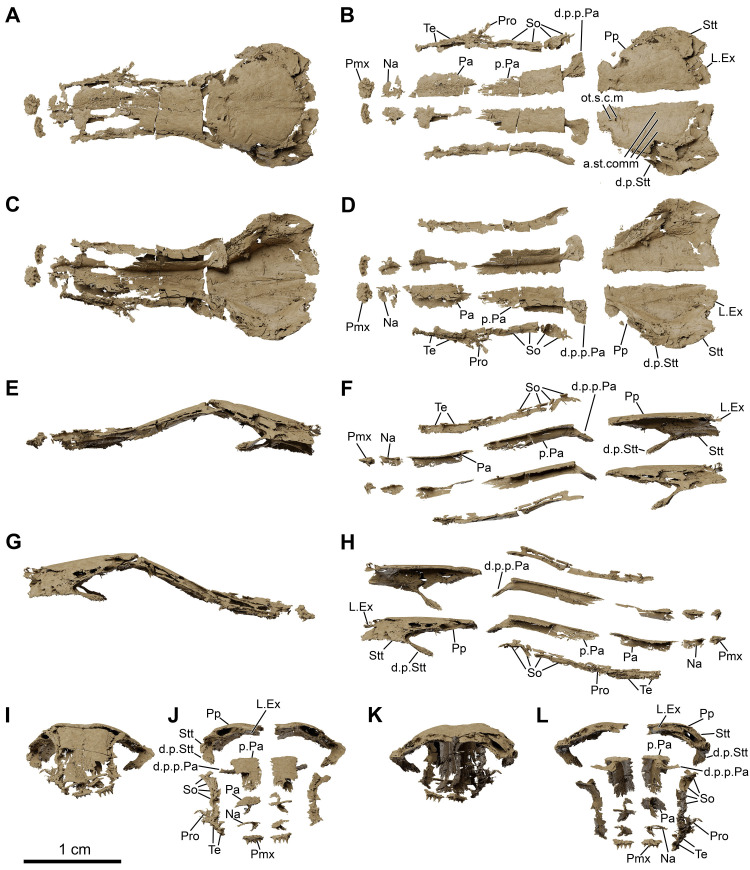
*Graulia branchiodonta* gen. et sp. nov., MHNG GEPI V5788, texturized surface-rendered 3D models of the skull roof, obtained from synchrotron microCT. The skeletal elements in A,C,E,G,I,K are shown in natural position as they were retrieved in the fossil specimen. (A) dorsal view of the skull roof. (B) dorsal view of the skull roof with skeletal elements spaced apart. (C) ventral view of the skull roof. (D) ventral view of the skull roof with skeletal elements spaced apart. (E) left lateral view of the skull roof. (F) left lateral view of the skull roof with skeletal elements spaced apart. (G) right lateral view of the skull roof. (H) right lateral view of the skull roof with skeletal elements spaced apart. (I) anterior view of the skull roof. (J) anterior view of the skull roof with skeletal elements spaced apart. (K) posterior view of the skull roof. (L) posterior view of the skull roof with skeletal elements spaced apart. A high-resolution version (1200 dpi) of this figure can be downloaded from Figshare (DOI: 10.6084/m9.figshare.26166943).

**Fig 21:**
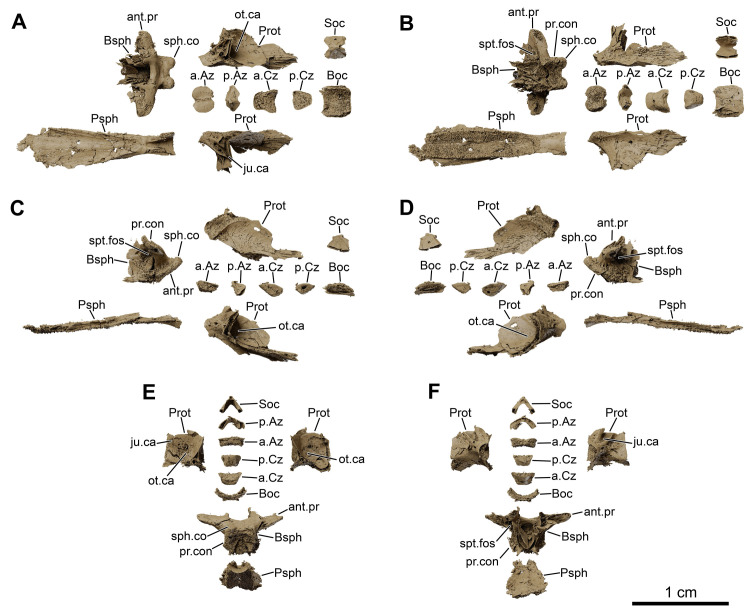
*Graulia branchiodonta* gen. et sp. nov., MHNG GEPI V5788, texturized surface-rendered 3D models of ossified elements of the neurocranium, obtained from synchrotron microCT. (A) dorsal view of the neurocranium with skeletal elements spaced apart. (B) ventral view of the neurocranium with skeletal elements spaced apart. (C) left lateral view of the neurocranium with skeletal elements spaced apart. (D) right lateral view of the neurocranium with skeletal elements spaced apart. (E) posterior view of the neurocranium with skeletal elements spaced apart. (F) anterior view of the neurocranium with skeletal elements spaced apart. A high-resolution version (1200 dpi) of this figure can be downloaded from Figshare (DOI: 10.6084/m9.figshare.26166943).

**Fig 22:**
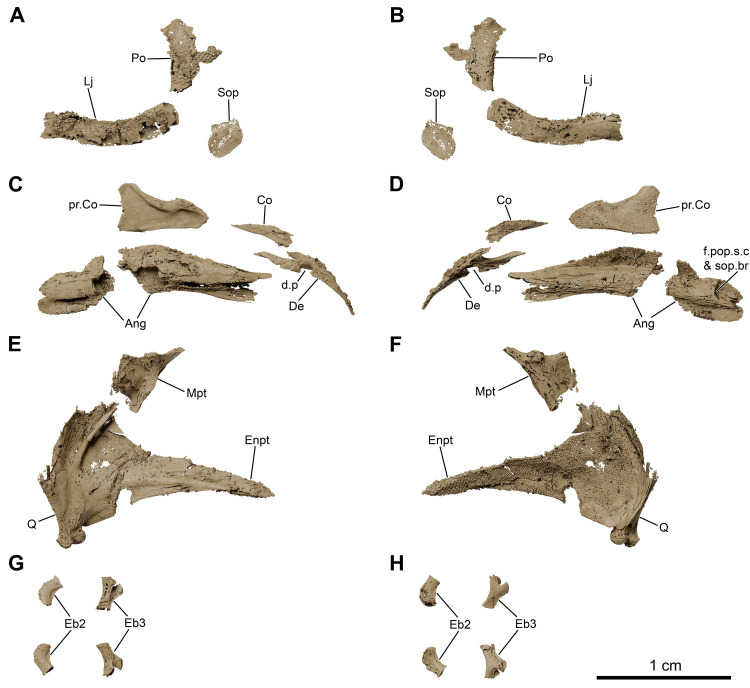
*Graulia branchiodonta* gen. et sp. nov., MHNG GEPI V5788, texturized surface-rendered 3D models of selected elements from the cheek, lower jaw, palate and branchial skeleton, obtained from synchrotron microCT. (A) lateral view of cheek. (B) medial view of cheek. (C) lateroventral view of lower jaw with skeletal elements spaced apart. (D) mediodorsal view of lower jaw with skeletal elements spaced apart. (E) laterodorsal view of palatoquadrate with skeletal elements spaced apart. (F) mediodorsal view of palatoquadrate with skeletal elements spaced apart. (G) dorsal view of epibranchials, spaced apart. (H) ventral view of epibranchials, spaced apart. A high-resolution version (1200 dpi) of this figure can be downloaded from Figshare (DOI: 10.6084/m9.figshare.26166943).

**Fig 23:**
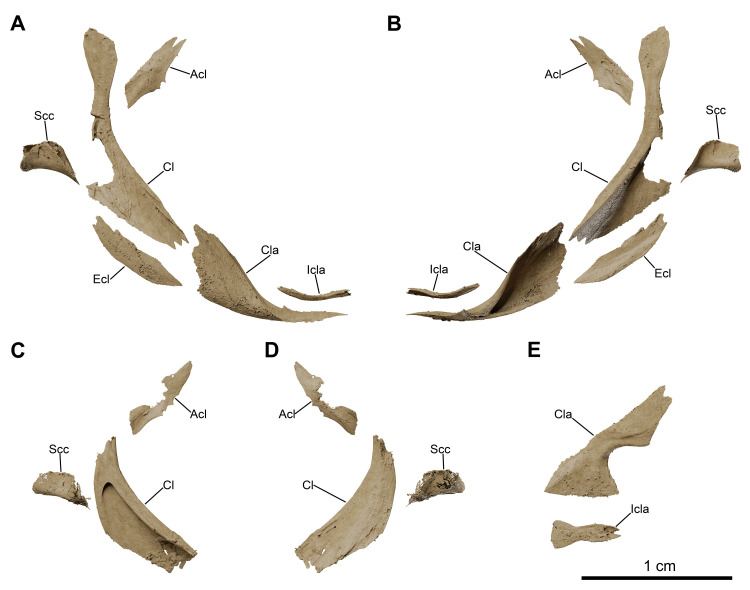
*Graulia branchiodonta* gen. et sp. nov., MHNG GEPI V5788, texturized surface-rendered 3D models of the pectoral girdle, obtained from synchrotron microCT. (A) lateral view of pectoral girdle, right half, with skeletal elements spaced apart. (B) medial view of pectoral girdle, right half, with skeletal elements spaced apart. (C) medial view of pectoral girdle, left half, with skeletal elements spaced apart. (D) lateral view of pectoral girdle, left half, with skeletal elements spaced apart. (E) dorsal view of clavicle and interclavicle from the right half. A high-resolution version (1200 dpi) of this figure can be downloaded from Figshare (DOI: 10.6084/m9.figshare.26166943).

**Fig 24:**
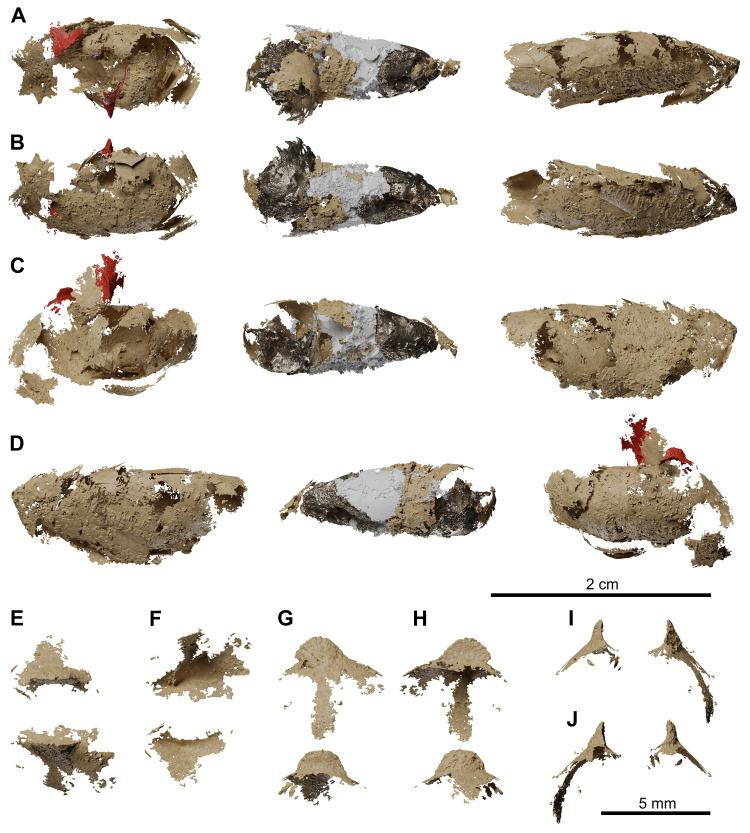
*Graulia branchiodonta* gen. et sp. nov., MHNG GEPI V5788, texturized surface-rendered 3D models of the lung, obtained from synchrotron microCT. The first lobe of the lung shows two dorsal crests colorized in red in A-D and with a bony texture in E-J. The synchrotron scan of the second lobe showed a very dense intrusion that has been rendered with a metallic shading and a very light intrusion that has been rendered in white opaque. (A) dorsal view of the three lobes of the lung. (B) ventral view of the three lobes of the lung. (C) left lateral view of the three lobes of the lung. (D) right lateral view of the three lobes of the lung. (E) dorsal view of dorsal crests of the first lobe. (F) ventral view of dorsal crests of the first lobe. (G) left lateral view of dorsal crests of the first lobe. (H) right lateral view of dorsal crests of the first lobe. (I) anterior view of dorsal crests of the first lobe. (J) posterior view of dorsal crests of the first lobe. A high-resolution version (1200 dpi) of this figure can be downloaded from Figshare (DOI: 10.6084/m9.figshare.26166943).

**Fig 25:**
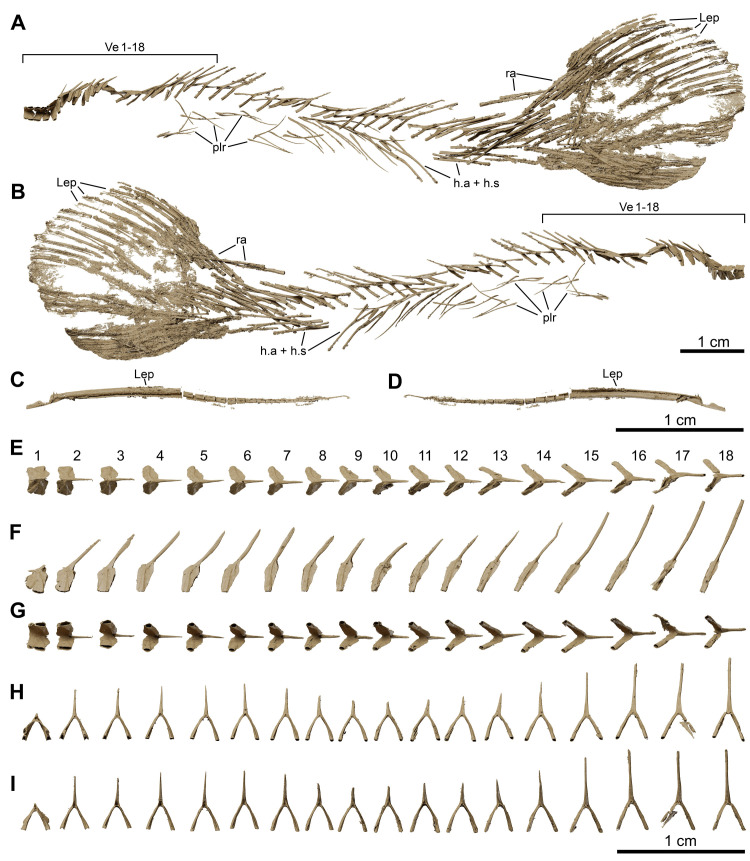
*Graulia branchiodonta* gen. et sp. nov., MHNG GEPI V5788, texturized surface-rendered 3D models of the postcranial axial skeleton and tail, obtained from synchrotron microCT. (A) left lateral view of postcranial axial skeleton and tail. (B) right lateral view of postcranial axial skeleton and tail. (C) left lateral view of isolated lepidotrichia of the tail; (D) right lateral view of isolated lepidotrichia of the tail; (E) dorsal view of vertebrae from 1 to 18. (F) left lateral view of vertebrae from 1 to 18. (G) ventral view of vertebrae from 1 to 18. (H) anterior view of vertebrae from 1 to 18. (I) posterior view of vertebrae from 1 to 18. A high-resolution version (1200 dpi) of this figure can be downloaded from Figshare (DOI: 10.6084/m9.figshare.26166943).

**Fig 26:**
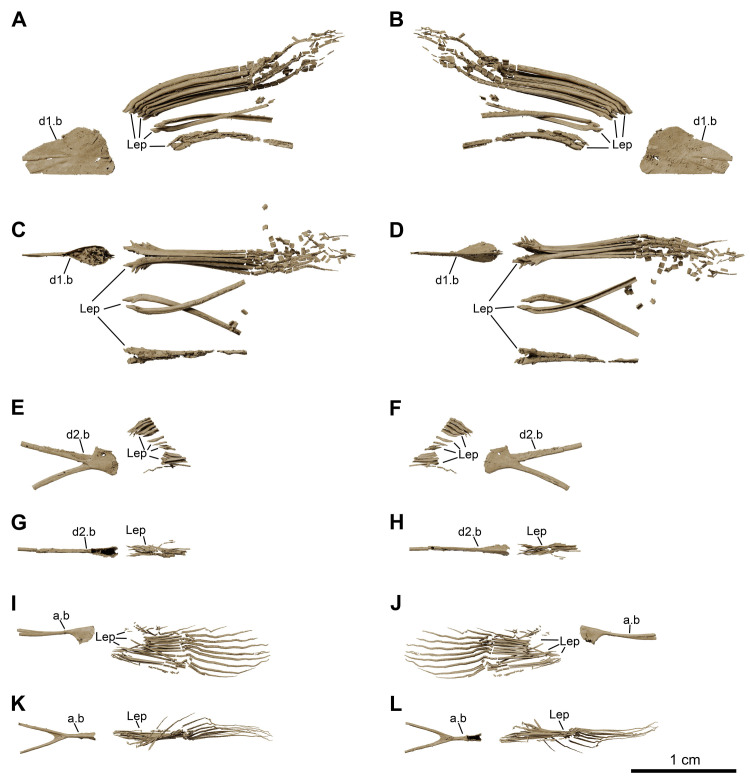
*Graulia branchiodonta* gen. et sp. nov., MHNG GEPI V5788, texturized surface-rendered 3D models of the unpaired fins, obtained from synchrotron microCT. (A) left lateral view of first dorsal fin. (B) right lateral view of first dorsal fin. (C) dorsal view of first dorsal fin. (D) ventral view of first dorsal fin. (E) lateral view of second dorsal fin. (F) medial view of second dorsal fin. (G) dorsal view of second dorsal fin. (H) ventral view of second dorsal fin. (I) left lateral view of anal fin. (J) right lateral view of anal fin. (K) dorsal view of anal fin. (L) ventral view of anal fin. A high-resolution version (1200 dpi) of this figure can be downloaded from Figshare (DOI: 10.6084/m9.figshare.26166943).

**Fig 27:**
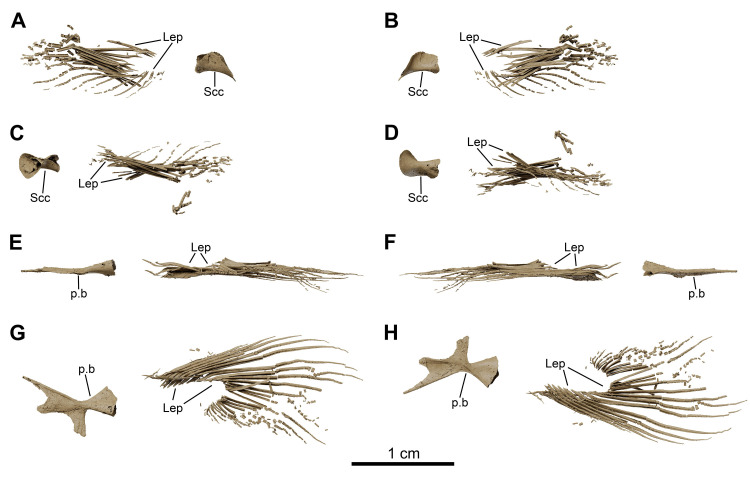
*Graulia branchiodonta* gen. et sp. nov., MHNG GEPI V5788, texturized surface-rendered 3D models of the paired fins, obtained from synchrotron microCT. (A) lateral view of right pectoral fin. (B) medial view of right pectoral fin. (C) dorsal view of right pectoral fin. (D) ventral view of right pectoral fin. (E) medial view of right pelvic fin. (F) lateral view of right pelvic fin. (G) dorsal view of right pelvic fin. (H) ventral view of right pelvic fin. A high-resolution version (1200 dpi) of this figure can be downloaded from Figshare (DOI: 10.6084/m9.figshare.26166943).

**Fig 28:**
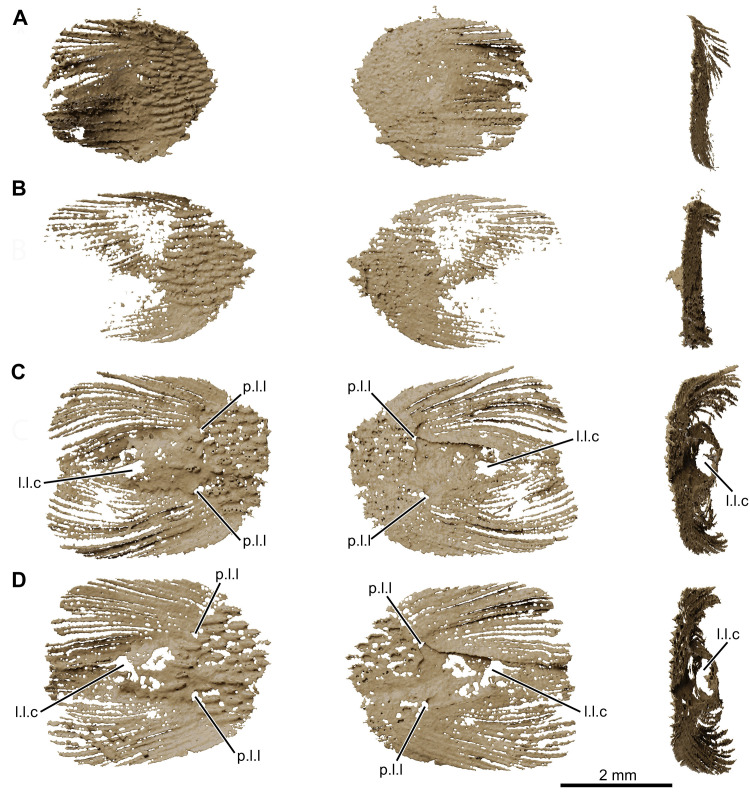
*Graulia branchiodonta* gen. et sp. nov., MHNG GEPI V5788, texturized surface-rendered 3D models of selected scales, obtained from synchrotron microCT. All scales come from the left side of the specimen. Each scale is shown in lateral, medial and frontal view, from left to right. (A) scale from the dorsal region next to the basal plate of the first dorsal fin. (B) scale from the flank region at the level of the basal plate of the first dorsal fin. (C,D) lateral line scales found at the level of the pelvic bones. A high-resolution version (1200 dpi) of this figure can be downloaded from Figshare (DOI: 10.6084/m9.figshare.26166943).

**Fig 29:**
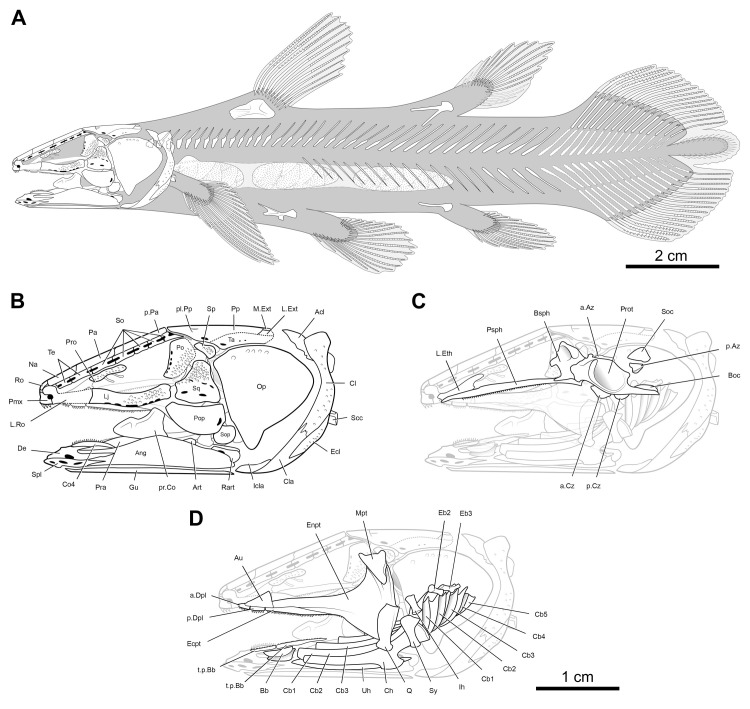
*Graulia branchiodonta* gen. et sp. nov., interpretative body reconstructions and detailed skull drawings in lateral view. (A) body profile. (B) lateral view of skull and pectoral girdle. (C) lateral view of skull and pectoral girdle with neurocranium highlighted. (D) lateral view of skull and pectoral girdle with palate and hyobranchial skeleton highlighted. A high-resolution version (1200 dpi) of this figure can be downloaded from Figshare (DOI: 10.6084/m9.figshare.26166943).

**Fig 30:**
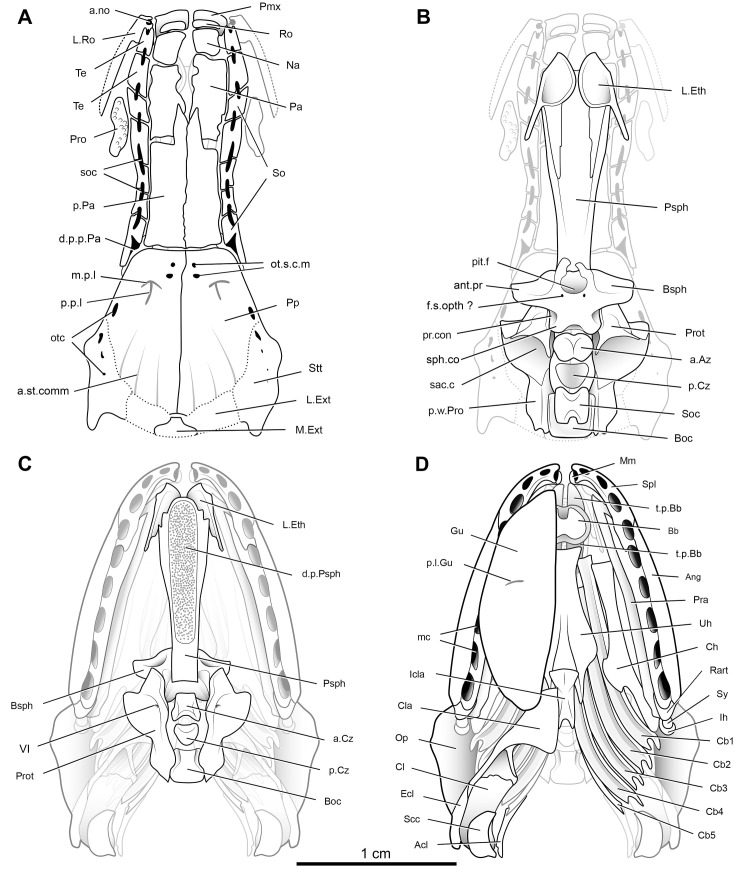
*Graulia branchiodonta* gen. et sp. nov., detailed skull drawings in dorsal and ventral view. (A) dorsal view of skull roof. (B) dorsal view of neurocranium. (C) ventral view of skull and pectoral girdle with neurocranium highlighted. (D) ventral view of skull and pectoral girdle with the left half of the pectoral girdle and the left gular plate moved to the background. A high-resolution version (1200 dpi) of this figure can be downloaded from Figshare (DOI: 10.6084/m9.figshare.26166943).

#### Dermal skull

The dermal skull includes elements from the cheek (lachrymojugal, postorbital, squamosal, preoperculum, suboperculum and spiracular), the operculo-gular series (operculum and gular), and the skull roof (premaxilla, rostrals, nasals, tectals, lateral rostral, preorbital, supraorbitals, anterior and posterior parietals, postparietals, tabulars (supratemporals), and extrascapular series), with a different degree of preservation. Several bones of the skull roof have been damaged during preparation, with a general loss of details of the dorsal surface of the bones and some smaller elements likely missing.

#### Skull roof (Figs 5–7, 20, 30)

Both parietonasal and postparietal shields are present, but they differ in preservation state. The postparietal shield is slightly shorter than the parietonasal shield, the two are not sutured to each other. The posterior margin of the postparietal shield (comprising the postparietals, tabulars (supratemporals) and extrascapular series) is badly preserved and the shape and connections of the bones are difficult to identify in dorsal view, but sutures between the tabulars (supratemporals), postparietals and lateral extrascapulars are visible in ventral view. The parietonasal shield appears to be slightly larger than the postparietal shield; it is relatively narrow throughout its length, with parallel lateral margins, while the postparietal shield broadens posteriorly. All skull roof bones are smooth and do not carry any significant ornamentation, which could be due to excessive polishing during preparation. This is supported by the fact that the preorbital of MHNG GEPI V5787, shifted deeper in the matrix, as well as several bones of the cheek are instead strongly ornamented with tubercles, suggesting a similar ornamentation pattern for other dermal bones of the skull roof.

#### Parietonasal shield

*Premaxilla*. Two premaxillae (Pmx) compose the anterior most margin of the skull. The bones are symmetrically arranged around the tip of the snout, they are rectangular in outline and do not present a dorsal lamina. The premaxillae of MHNG GEPI V5787 bear three large teeth, while those of MHNG GEPI V5788 bear four teeth, a clear example of intraspecific variation. The posterior margin is slightly concave, anteriorly framing the ethmoidal commissure of the infraorbital sensory canal. The postero-lateral margin of the left premaxilla is notched, marking the position of the anterior nostril.

*Rostral*. One pair of rostrals (Ro) is present at the anteriormost edge of the parietonasal shield. They are small and delicate bones, and bear a ventral process, possibly posteriorly framing the course of the ethmoidal commissure of the infraorbital sensory canal.

*Lateral rostral*. The lateral rostrals (L.Ro) are very elongated posteriorly but badly preserved so we segmented only the right one (MHNG GEPI V5787). The bone is composed of two portions: a broad anterior section for the dorsal loop of the infraorbital sensory line canal, and a thinner, posteriorly elongate posterior portion. The posterior segment bears a prominent descending process articulating with the lateral ethmoid and a canal for the passage of the infraorbital sensory line canal, visible on the internal surface, posteriorly prolonging itself into the lachrymojugal. The exact location of the anterior and posterior nostrils as well as the presence of pores cannot be confidently stated due to the incomplete preservation of the external surface.

*Preorbital*. Only the left preobital (Pro) has been confidently identified. It is an elongate and flat bone with two distinctive descending processes ventrally, possibly protecting the walls of the rostral organ towards its posteriormost exit pore. The preorbital is ornamented by numerous rounded tubercles, possibly representing the unpreserved ornamentation pattern of other dermal bones of the skull roof.

*Nasal*. One pair of nasals (Na) can be identified. Both bones are roughly squared in outline, with the right nasal being more badly preserved than the left one. The supraorbital lateral line canal (soc) runs through a deep trench on the lateral side of both bones, continuing its path into the anterior parietals.

*Tectals*. Two pairs of tectals (anterior and posterior tectals) (Te) lie anteriorly to the first supraorbitals. They bear a ventral process that opens medially and prolongs itself posteriorly into the supraorbital series for the passage of the supraorbital lateral line canal (soc). Two distinctive notches visible in the right posterior tectal likely correspond to pores associated with the course of this sensory canal, similar to those present in the supraorbital series.

*Supraorbital series*. Several small and disarticulated supraorbital bones (So) have been identified, forming a series of 5 elements on each side of the skull. These butterfly-shaped bones are composed of two thin horizontal laminae joined by a narrow small vertical column for the passage of the supraorbital lateral line canal (soc) between the external and internal laminae. The supraorbital canal opens through elongate pores at the sutural contact between the bones.

*Parietals*. Two pairs of parietals are present, the anterior pair being slightly smaller than the posterior one. The anterior parietals (Pa) are badly preserved and lack any distinctive features albeit a ridge on the ventral surface associated to the passage of the supraorbital lateral line canal. The posterior parietals (p.Pa) are more completely preserved, both bones have a rectangular outline and straight anterior and posterior margins. The path of the supraorbital lateral line canal can be seen through a ventral trench on the lateral side of both anterior and posterior parietals, laterally framed by the supraorbital series. The posterior parietals overlap the anterior parietals through a median quadrangular expansion. No distinct pores are visible on the external surface. The descending posteroventral processes of the posterior parietals (d.p.p.Pa) greatly broaden at the suture with the antotic processes of the basisphenoid.

#### Postparietal shield

*Postparietal*. The two postparietals (Pp) are entirely preserved and could be modelled separately. The anterior margin is straight without indentations forming the intracranial joint. The posterior margin is slightly embayed. The ventral surface carries a descending process (d.p.Pp) in the shape of a broad longitudinal ridge with a deep lateral funnel for the passage of the otic sensory canal. The sensory canals are greatly developed in each postparietal; the large cavity for the passage of the otic sensory canal (otc) opens laterally with two pores and anteroventrally with a huge pore to receive the canals from the cheek (infraorbital) and the parietals (supraorbital) respectively. The medial branch of the otic sensory canal (o.t.s.c.m) can be clearly identified with two openings, one anterior smaller and one posterior larger, close to the midline. The dorsal surface of the postparietals appears smooth and lack any distinctive ornamentation. However, faint antero-posteriorly oriented grooves can be seen in MHNG GEPI V5788, radiating from the posterior margin of the postparietals. These could represent anterior branches of the supratemporal commissure (a.st.comm) running through cartilage or soft tissue as in *Latimeria*, leaving only shallow grooves on the external surface of the bones. Similarly, in the anterior portion of the postparietals in MHNG GEPI V5787, deeper grooves likely correspond to pit-lines, as found in other fossil coelacanths, developed as a short transverse middle pit-line (m.p.l) and a longer longitudinal posterior pit-line (p.p.l).

*Supratemporal*. The sutures between the supratemporals (Stt) and the postparietals are solely visible ventrally in MHNG GEPI V5787 and MHNG GEPI V5788, marking a relatively large supratemporal. The supratemporal contains the junction of the otic canal (otc), the supratemporal commissure and the main lateral line canals, as evidenced by a large pore on the antero-lateral corner and smaller pores posteriorly following the course of the sensory canal. No supratemporal is sufficiently well preserved to allow distinctive pores to be reconstructed. The long and narrow descending processes are anteriorly oriented.

*Lateral extrascapular series*. The posterior margin of the skull roof of MHNG GEPI V5787 is badly preserved, with numerous small bony fragments (ossicles) of indeterminate shape, likely representing the fragments of the extrascapular series. However, in ventral view one large element corresponding to a lateral extrascapular (L.Ex) can be identified. In MHNG GEPI V5788, the ventral surface of the postparietal shield reveals two large ossifications carrying a canal that runs parallel to the natural posterior margin of the postparietal shield. These likely represent two large, roughly triangular lateral extrascapulars that may have laterally framed the median extrascapular. The posterior margin of the postparietal shield was thus mainly formed by the postero-lateral corner of the supratemporal and the two lateral extrascapulars.

*Median extrascapular*. A disarticulated but badly preserved median extrascapular (M.Ex) has been identified, clearly not sutured to the postparietals. The bone has a butterfly shape and carries a canal for the supratemporal commissure, with a median anterior projection that may have fitted in a gap of the posterior margin of the postparietals.

#### Cheek (Figs 5, 6, 10, 22, 29)

The bones of the cheek are extremely thin and have been partly damaged during preparation, especially at the level of the postorbital and the lachrymojugal. The cheek comprises the lachrymojugal, postorbital, squamosal, preoperculum, suboperculum, and spiracular. No sclerotic ossicles have been found. Many of the cheek bones are ornamented with small ovoid tubercles. Given the size and position of the bones, we would expect a small orbital space mostly occupied by the eye. The lachrymojugal, postorbital, squamosal and preoperculum show large cavities housing sensory canals, associated with a series of pores of different sizes. No pit lines have been confidently identified.

*Lachrymojugal*. The lachrymojugal (Lj) is large, curved and broad, with parallel margins. It ventrally frames the orbit and carries a large canal for the passage of the infraorbital sensory canal. The anterior portion is not particularly expanded nor angled and ends in a vertical edge. The external surface is ornamented with large rounded tubercles, running parallel to the margins of the bone. The ventrolateral margin of the bone presents numerous pores, associated with the course of the sensory canal.

*Postorbital*. The postorbital (Po) is badly preserved, represented mainly in MHNG GEPI V5787 by the internal wall of the cavity of the infraorbital sensory canal, which runs through the postorbital into the postparietal, where it merges with the otic canal. The ornamented external surface of the bone is only partially preserved in MHNG GEPI V5788, along with a fragment of a thin posterior expansion, still bearing ornamentation composed of small rounded tubercles. However, due to its bad preservation, the true extension of this posterior expansion is difficult to reconstruct. Some small pores can be identified along the anterior edge of the postorbital, suggesting that the sensory canal runs close to the anterior margin of the bone, but we could not identify an anterior excavation. The postorbital was likely positioned behind the intracranial joint.

*Squamosal*. The squamosal (Sq) is a large and broad bone, slightly dorsally expanded, and positioned in the middle of the cheek, not reaching the skull roof. The bone does not show any overlapping surfaces for the lachrymojugal and the postorbital anteriorly, suggesting that it lied detached from these bones, however the posterior margin displays a narrow overlapping facet for the anterior edge of the operculum, revealing a close contact between the squamosal and the operculum. It is densely ornamented by large rounded tubercles, concentrated in the postero-dorsal portion of the bone. The squamosal carries the jugal canal that runs posteriorly into the preoperculum, as evidenced by a series of large pores revealing the course of the canal on the external surface. The path of the canal can also be clearly followed on the internal surface: it enters the squamosal anteriorly at the level of the posterior margin of the lachrymojugal, and exits on the postero-ventral corner of the bone.

*Preoperculum*. The preoperculum (Pop) is a thin bone displaying a large anterior blade-like expansion and a broad canal posteriorly, well visible in internal view. It is located immediately ventral to the squamosal and has roughly the same length as the latter. No overlapping surfaces for the adjoining bones have been clearly identified. As opposed to other cheek bones, it is not ornamented. The jugal canal running through the preoperculum from the squamosal is neatly followed by a large dorso-ventrally oriented canal located on the posterior portion of the bone, carrying the sensory canal into the lower jaw.

*Suboperculum*. The suboperculum (Sop) is a small bone, preserved only in MHNG GEPI V5788. It has a rounded, scale-like outline, and is slightly convex externally and concave internally. No clear overlapping surfaces have been identified due to its poor preservation. The suboperculum was likely located between the posterior margin of the preoperculum and the antero-ventral corner of the operculum, possibly overlapped by the former as in *Latimeria*.

*Spiracular*. A small spiracular bone (Sp) lies between the operculum and the skull roof, posterior to the postorbital but not tightly sutured to other bones of the cheek. However, it shows a narrow depressed area on its antero-ventral margin, probably framing the dorsal margin of the spiracular groove or being overlapped by the unpreserved dorsal margin of the postorbital. The posterior margin of the bone also displays a small overlapping surface, suggesting that a dorsal overlap by the supratemporal may have occurred. Despite its small size, the spiracular is strongly ornamented with rounded tubercles on its flat external surface, while the internal surface is deeply concave.

#### Operculo-gular series (Figs 5, 6, 10, 22, 29)

The operculo-gular series is represented by large opercular and gular bones from both sides of the skull. No median gular nor branchiostegal rays are present.

*Operculum*. The operculum (Op) is the largest preserved bone of the skull. In MHNG GEPI V5787, only the left operculum has been modelled but it is completely preserved. The dorsal and posterior margins are clearly rounded. The ventral margin is stretched by a bony projection forming a triangular antero-ventral corner. The operculum is overall flat, only gently concave, without raised areas, excepting a thicker anterior margin. The external surface is ornamented with few slightly elongate, pointed tubercles, more concentrated in the dorsal portion, while the rest of the bone is smooth.

*Gular*. Two large gular bones (Gu) ventrally floor the intermandibular space. The median contact between the two bones is straight and they laterally overlap the ventral margin of the angulars from both sides of the skull. Both bones are unornamented but a median pit line (gu.p.l) can be identified on the left gular in MHNG GEPI V5787, close to the ossification centre.

#### Lower jaw (Figs 5, 6, 9, 22, 29, 30)

The highly apomorphic lower jaw of coelacanths is here represented by the dentary, splenial, angular, prearticular, mentomeckelian, articular and retroarticular, and two coronoids (4^th^ and 5^th^) from both sides of the skull. No submandibulars are present.

*Dentary*. The dentary (De) is a narrow but complex bone of the mandible. It is antero-posteriorly elongated with an enlarged anterior margin, medially bending but not forming the symphysis of the lower jaw. The posterior end of the bone is hook-shaped, with the medial tip of the hook connecting to the 4^th^ coronoid, while the lateral tip contacts the angular. The dentary does not carry any associated tooth plates. However, such tooth plates are loosely attached to the dentary in *Latimeria*, which indicates that, if present here, they may have shifted away. Another explanation would be that the anteriormost part of the skull has been excessively polished, accidentally removing the tooth plates. The dentary is pierced by a large pore (d.p) at the suture with the splenial.

*Mentomeckelian*. The mentomeckelian (Mm) has been preserved as a small, lightly ossified, and roughly triangular element in lateral view located at the symphysis of the lower jaw, enclosed between the splenial, the dentary and the anterior tip of the prearticular. It displays a flat circular anterior margin forming the mandibular symphysis and as it broadens posteriorly it becomes laterally concave, probably framing the anterior portion of the mandibular sensory canal.

*Splenial*. The splenial (Spl) is the most anterior element of the reduced infradentary series in coelacanths. It is anteriorly enlarged and slightly angled downwards. Large foramina associated with the course of the mandibular sensory canal run through the ventral margin of the bone. Four pores associated with the course of the mandibular sensory canal are located entirely on the splenial: one at the jaw symphysis (symphysial pore) and three lateroventrally. These large pores are followed posteriorly by similar ones in the angular. A supplementary pore spans the suture with the angular posteriorly. No distinctive ornamentation has been identified.

*Prearticular*. The prearticulars (Pra) from both sides of the skull are long bones, almost entirely preserved. Both prearticulars are as long as the angular and lingually frame it. The bones are overall rectangular in shape, becoming deeper in its anterior portion and decreasing in height towards the rear. The lingual side of the prearticular is covered with a shagreen of small denticles. The prearticular contacts the angular anteriorly, close to the mentomeckelian, but not posteriorly where their connection is mediated by the retroarticular.

*Angular*. The angular (Ang) is the second element of the infradentary series and the largest bone of the lower jaw. The posterior portion is narrower than the middle portion. The bone deepens anterior to the retroarticular forming a coronoid expansion, and becomes narrower again anteriorly with a peculiar two-pronged anterior tip. The external (labial) surface is unornamented and no pit lines have been confidently identified. The ventral margin carries a narrow but elongate overlapping surface for the gular. The bone is medially inflated by the course of the mandibular sensory canal. The canal penetrates the angular posteriorly at the level of the articular glenoid fossa, descending from the preoperculum in the cheek into the lower jaw. The canal then runs antero-posteriorly within the angular through a large lumen in the bone, carrying the mandibular sensory canal and the mandibular ramus of nerve VII. The lingual side of the angular, framed by the prearticular, carries the external mandibular ramus of nerve VII but no distinctive grooves could be modelled due to the scanning resolution. A series of small pores occur on the lingual side, following the course of the canal, probably for the innervation of the neuromasts. The angular displays four additional lateroventral pores on its labial side, following those of the splenial, plus a dorsal pore at the suture with the retroarticular for the preopercular and subopercular canals.

*Coronoids*. The first coronoids are absent, likely eroded during preparation of the specimen (MHNG GEPI V5787), and thus an exact number of coronoids cannot be confidently stated. We found a large putative fourth coronoid (Co), partly damaged at his anterior tip. It displays a small postero-ventral projection for the contact with the angular. It is ornamented by numerous small denticles, slightly more developed and pointed along the dorsal margin, and carries no fangs.

*Principal coronoid*. As in other coelacanths, the 5^th^ coronoid (pr.Co) is the largest element of the coronoid series and has a characteristic shape. It lies at the level of the deepest part of the angular and is separated from the more anterior coronoids by a diastema. It has an elongate shape, with a prominent dorsal process oriented posteriorly and a neat concavity on its dorsal edge. The lingual surface is covered by small denticles, similar to the ones on the prearticular and the smaller coronoid anteriorly. The labial surface carries a strong ridge across the dorsal margin, associated with the course of the adductor muscle. The ventral edge may have fit into a small gap on the wide dorsal edge of the prearticular.

*Articular and retroarticular*. The articular (Art) and retroarticular (Rart) bones represent the posterior portion of the Meckelian ossifications. The articular-retroarticular complex forms the glenoid fossa for the articulation with the quadrate, and because both bones were found clearly separated from each other, we suspect that a cartilage infilling of the glenoid fossa connected them. The retroarticular bears a pronounced lateral process and displays a small articulation surface for the symplectic posteriorly. The lateral process forms the posterior wall of the dorsal pore associated with the subopercular and preopercular sensory canals (f.pop.s.c & sop.br). The two sensory canals likely merged before entering the jaw, as in *Latimeria*.

#### Palate (Figs 5, 6, 8, 11, 22, 29, 30)

The almost complete series of bones forming the palate has been retrieved from both sides of the skull: the palatoquadrate complex, the autopalatine, two dermopalatines, the ectopterygoid, and the median parasphenoid. Unfortunately, no vomers have been identified.

*Palatoquadrate complex*. Both palatoquadrate complexes, comprising both dermal (entopterygoid) and endoskeletal (quadrate and metapterygoid) elements, are fairly well preserved.

*Entopterygoid*. The entopterygoid (Entp) is entirely preserved. It is slender and anteriorly elongate, with a roughly triangular shape, characteristic of coelacanths. The lingual side is slightly concave and covered in a shagreen of small denticles. The dorsal edge is concave, with a semi-circular outline, while the ventral margin is straight and displays a narrow antero-posterior depression for the contact with the lingual lamina of the ectopterygoid. The anterior projection is dorsally concave and would have probably housed a thin sheet of cartilage for the attachment of the autopalatine, as in *Latimeria*. A shallow ventral swelling also occurs in the portion connecting the anterior expansion with the postero-ventral corner, overlain by the quadrate.

*Metapterygoid*. The metapterygoid (Mpt) could be modelled separately on the right side of MHNG GEPI V5788 but not in MHNG GEPI V5787 where it is tightly fused to the entopterygoid. The dorsal margin has a saddle-like articulatory surface for the antotic process of the basisphenoid from the braincase. The space between the metapterygoid and the quadrate may have been filled with a sheet of cartilage connecting both bones and lining the labial surface of the entopterygoid, similar to the condition in *Latimeria*. A strong ridge runs antero-laterally across the metapterygoid and prolongs itself ventrally onto the entopterygoid, parallel to its posterior margin.

*Quadrate*. The quadrate (Q) is an elongate and robust element, vertically oriented, but only weakly ossified internally. The posterior margin is formed by a blunt crest, while the anterior margin is concave to accommodate the postero-ventral corner of the entopterygoid. The ventral end carries a double condyle for the articulation with the glenoid fossa of the lower jaw; the articular heads were probably capped with cartilage.

*Autopalatine*. The autopalatine (Aut) is short and triangular in outline. It would have articulated posteriorly with the anterior most tip of the entopterygoid and dorsally with a ventral depression of the lateral ethmoid, and held in place by a sheet of cartilage.

*Dermopalatines and ectopterygoid*. Two dermopalatines and one ectopterygoid have been found in natural articulation. The two dermopalatines (anterior and posterior dermopalatines) (Dpl) are small, roughly equal in size. The ectopterygoid (Ecpt) is elongate, broadening posteriorly, and twice as large as both dermopalatines combined. The dermopalatines show a flat, slightly concave dorsal surface. The posterior dermopalatine articulates with the autopalatine, while the anterior dermopalatine articulates with the lateral ethmoid, only contacting the anterior tip of the autopalatine. The dorsal surface of the ectopterygoid carries a groove for articulation with the ventral margin of the anterior projection of the entopterygoid. The dentition of both the dermopalatines and ectopterygoid is organized into various rows of small teeth, slightly larger in the posterior dermopalatine, and carried on a horizontal lamina close to the labial side; there are no distinctive larger fangs or replacement sockets.

*Parasphenoid*. The parasphenoid (Psph) is entirely preserved. It is a broad bone, with a roughly rectangular outline, widening anteriorly. The ventral surface is slightly concave and carries an elongate, blade-like and anteriorly rounded dental plate, bearing a shagreen of small denticles, and covering most of the palatal surface of the bone. The dorsal surface is composed of two short and slightly laterally curved lateral flanges (or ascending laminae) contacting the lateral ethmoids and delimiting a flat median surface between their ridges, which converge posteriorly at the level of the articulation with the basisphenoid. The unornamented posterior portion is broad and its dorsal margin comprises a pit to accommodate the anterior expansion of the basisphenoid. No open buccohypophysial canal has been identified.

#### Neurocranium (Figs 5, 6, 8, 21, 29, 30)

Numerous elements of the neurocranium have been retrieved both from the ethmosphenoid and otico-occipital portions. These include the lateral ethmoids, basisphenoid, prootics, basioccipital, and supraoccipital. There are no traces of exoccipitals. The zygal series include two anterior and posterior anazygals and catazygals. The separate ossifications of the parasphenoid, basisphenoid, prootics, and basioccipital confirm that the braincase was not ossified as a single unit.

*Lateral ethmoid*. The two large lateral ethmoids (L.Eth) have been retrieved, separated from each other and not meeting in the midline. Each lateral ethmoid is composed of three distinct portions: an anterior roughly circular concavity in dorsal view, a long postero-dorsal process that contacts the skull roof, and a pointed posterior projection for the overlapping of the parasphenoid. The lateral wall of the postero-dorsal process is marked by a shallow trough for the contact with the autopalatine.

*Basisphenoid*. The median basisphenoid (Bsph) is entirely preserved. The paired *processus connectens* (pr.con) are developed as blunt and elongated postero-lateral margins that partially meet the parasphenoid; their unfinished margins were certainly capped by large cartilage pads to articulate with the otic shelves of the prootics. The antotic processes (ant.pr) are fairly well preserved, roughly triangular in shape in dorsal view, with dorsal sutural surfaces for the descending process of the posterior parietal encompassing the U-shaped embayment between the antotic processes. The *dorsum sellae* forms a relatively broad median groove, dorsally framing the pituitary fossa (pit.f). A pair of sphenoid condyles (sph.co) separated by a gap are well developed in the posterior edge for the articulation with the anterior anazygal. In ventral view, a large notochordal pit is anteriorly bordered by a small ridge and laterally by the ventral edge of the *processus connectens*. Anteriorly, the elongate surfaces of attachment for the parasphenoid form hollow cavities separated by a median ridge embracing the postero-dorsal furrow of the parasphenoid. Ventral to the antotic processes, lies the suprapterygoid fossa (spt.fos), the point of origin of the *adductor palatini* muscles. The suprapterygoid fossa is pierced by the foramen for the *ramus ophthalmicus profundus* (V1) of the trigeminal nerve (V). Two pores open dorso-medially to each of the antotic processes at the junction with the *dorsum sellae*, probably representing the foramina for the trochlear nerve (IV) and the superficial ophthalmic ramus of the trigeminal nerve (V) (f.s.opth?) that would have pierced the basisphenoid.

*Prootic*. Both prootics (Prot) are entirely preserved as separate elements, forming the lateral commissure of the neurocranium. The otic shelf is short and carries a deep dorso-medial groove to accommodate the *processus connectens* of the basisphenoid. The dorsal and posterior margins of the prootics appear jagged due to the absence of preservation of an extensive cartilaginous connection with the rest of the braincase. The lateral margins are relatively thin and badly preserved and thus the hyomandibular facet cannot be confidently reconstructed as it was probably made of cartilage entirely. Dorsal to the otic shelf, a small antero-dorsal projection (the prefacial eminence) (pr.em) corresponds to a small ascending process to connect to the postparietals, suggesting that the temporal excavation was lined with cartilage. Lateral to it, the small overlapping surface for the descending process of the supratemporal is elongate and antero-ventrally oriented. The medial surface is smooth and slightly concave, laterally framing the notochord. The ventral surface carries a groove for the course of the basicranial muscle, which probably inserted on the posterior wing of the prootic. The saccular chamber (or otic capsule) (ot.ca) is large, ovoid in shape, and postero-dorsally open. It housed the otoliths but none were preserved. A thin medial wall separates the saccular chamber from the notochordal canal. The ventral floor of the saccular chamber passes posteriorly into the posterior wing of the prootic, which is medially inclined and terminated by a margin for the suture with the basioccipital. No large distinctive canals have been identified, although a small foramen ventral to the otic shelf could correspond to the canal for the orbitonasal artery. The jugular canal (ju.ca), containing the jugular vein and the hyomandibular ramus of the facial nerve (VII), is not enclosed dorsally by bone, likely having a cartilaginous roof. It is represented here as a deep thin grove starting at the base of the prefacial eminence, dorsal to the posterior edge of the otic shelf, and exits laterally. The ventral surface is pierced by a large pore for the abducens nerve (VI) to innervate the basicranial muscle and the lateral rectus eye muscle. There are other pores that cannot be attributed to a specific nerve or vein.

*Supraoccipital*. The supraoccipital (Soc) is a butterfly-shaped bone, strongly arched ventrally and forming the dorsal framing of the foramen magnum at the rear or the braincase. The posterior edge is thickened by two lateral depressions, possibly covered in cartilage and contacting the neural arch of the first vertebra. The supraoccipital is crossed medially by the foramen for the supradorsal ligament. The same foramen is visible in the neural arches.

*Basioccipital*. The basioccipital (Boc) is entirely preserved, detached from the rest of the ossified braincase. It has semi-lunar shape, being dorsally concave and ventrally convex. The dorsal surface is smooth and perfectly semi-circular to accommodate the ventral portion of the notochord. The antero-lateral margins are thin and likely sutured with the posterior wings of the prootics.

*Zygals*. The complete zygal series is preserved, comprising two anazygals and two catazygals. The zygal bones are median endoskeletal elements that surround and support the unconstructed notochord in the otic region.

*Anazygals*. The first or anterior anazygal (a.Az) lies dorsal to the notochord, articulating with the basisphenoid. This large anazygal is well ossified, has a quadrangular saddle shape, and is dorsally convex with a faint midline groove, and ventrally concave. It articulates with the basisphenoid condyles via two well-developed anterior depressions separated by a narrow gap. The anterior anazygal is the most commonly found and described anazygal among coelacanths, however in *Latimeria* a second anazygal has been described by Millot and Anthony in 1958 as a “basioccipital suschordal” [41], located dorsal to the notochord at the level of the basioccipital, but this bone has been poorly illustrated and, to our knowledge, has not been described previously in any other fossil coelacanth. Here, a second or posterior anazygal (p.Az) is identified as follows: it is a butterfly-shaped bone with a narrow median portion, smaller than the anterior anazygal and has a convex dorsal margin and a concave ventral margin to accommodate the dorsal portion of the notochord. Given the differences in shape between the posterior anazygal of *L*. *chalumnae* and the one identified in the new taxon (Figs 8 and 21), we acknowledge that we may have misidentified such element being instead a highly derived first vertebra or the supraoccipital. If so, the bone that we have identified as the supraoccipital may be instead the first or second vertebra and the posterior anazygal may be absent in the new taxon. Further studies on additional specimens may clarify this issue.

*Catazygals*. The catazygals are semi-lunar bones, dorsally concave and ventrally convex, without articulation facets as they do not contact any bone of the neurocranium. The catazygals ventrally frame the notochord, occupying the basicranial fenestra, and are posteriorly followed by the basioccipital. The anterior catazygal (a.Cz) is quadrangular in outline, slightly larger and more ossified than the posterior catazygal (p.Cz), which has a more triangular shape in dorsal view.

#### Hyoid and branchial arches (Figs 5, 6, 12, 22, 29, 30)

The hyoid and branchial arches are overall complete on each side of the skull. The hyoid arch comprises the symplectic, interhyal, and ceratohyal. There are five branchial arches composed of five ceratobranchials and two preserved epibranchials. No distinctive hypohyals, hypobranchials nor pharyngobranchials have been identified. The urohyal is entirely preserved, as is the single basibranchial.

#### Hyoid arch

*Symplectic*. The symplectic (Sy) has a characteristic inverted tripodal shape. The ventral portion is a cylindrical shaft and displays a circular ventral end, certainly covered in cartilage for the articulation with the retroarticular in the lower jaw. The enlarged triangular dorsal portion displays a concave internal surface, probably for the course of the posterior mandibuloid ligament, which runs on the inner side of the symplectic, connecting the retroarticular to the hyomandibula.

*Interhyal*. The interhyal (Ih) is found in close proximity to the ceratohyal and symplectic, with which it would have articulated. It is a small, elongate, weakly ossified tubular bone, with thin periosteal walls.

*Ceratohyals*. Both ceratohyals (Ch) are almost entirely preserved. They are curved, elongate elements with a well-developed and characteristic quadrangular ventro-lateral flange. The proximal portion is roughly triangular in shape with a concave external surface and displays a large proximal end to accommodate a probably well-developed cartilage pad for the articulation with the symplectic and the interhyal. The circular anterior edge is incompletely ossified and appears open as it was certainly connected to the basibranchial by a cartilaginous articulation.

#### Branchial arches

*Epibranchials*. Of all the elements from the epibranchial series, only two have been retrieved. The epibranchials (Eb) are small but stout bones, probably corresponding to the second and third epibranchials of the series. The putative second epibranchial (Eb2) is roughly cylindrical whereas the more median one (Eb3) is somewhat pear-shaped. The proximal articulation head of Eb3 is circular in outline but the distal one is bifid, divided into two ovoid articulatory facets separated by a groove, probably for the passage of an efferent branchial artery.

*Ceratobranchials*. The ceratobranchials (Cb) are the main elements of the branchial arches. They are five in number, decreasing in size postero-medially, and all display a similar curved shape with an elongate, cylindrical ventral section and a shorter, trapezoidal dorsal section. The ventral surface of the first ceratobranchials (Cb1-4) is characterized by a large groove for the passage of the efferent branchial arteries. The fifth ceratobranchial (Cb5) is the smallest of the series, lacks a distinctive groove and is inwardly curved, probably articulating with the anterior ceratobranchial (Cb4), as in other sarcopterygians. The first ceratobranchial (Cb1) is the longest one. The anterior tips of the ceratobranchials are circular in outline and appear open since they were probably capped with large cartilaginous heads connected to the basibranchial plate. Each ceratobranchial is covered dorsally by a shagreen of tiny villiform denticles, crossed by longitudinal series of small tooth plates bearing large pointed teeth, functionally analogous to the gill rakers of other fishes [42].

*Basibranchial*. A single basibranchial plate (Bb) is entirely preserved. It displays a roughly diamond-shaped in outline, without traces of partial division. It is only weakly ossified internally and composed of a perichondral bone shell encasing a mainly cartilaginous internal portion. The dorsal surface is slightly convex with two lateral depressions bordering a more elevated median section. The ventral surface is overall concave. The lateral margins would have housed cartilaginous articulation surfaces for the first four ceratobranchials laterally and for the ceratohyal anteriorly. Ventrally lies a large, posteriorly oriented circular facet that was also probably covered by a cartilage pad for the connection to the urohyal. Two sets of large dental plates (t.p.Bb) are associated with the basibranchial, the anterior pair being smaller and stouter than the more elongate posterior one. Both pairs of tooth plates carry small denticles, uniform in size, different from the single pointy teeth from the ceratobranchials. The size of the dental platelets relative to the ossified basibranchial indicate that the basibranchial may have been considerably larger in life and encased within large cartilage pads for the articulation with the urohyal, ceratohyal and ceratobranchials.

*Urohyal*. The well-ossified urohyal (Uh) is entirely preserved and has the characteristic inverted Y-shape of coelacanths. It is dorso-ventrally flattened and is composed of a wide bifid posterior portion narrowing anteriorly. The posterior expansion displays two lateral flanges with small ridges framing the median gap in the ventral surface and a prominent median ridge running across the dorsal surface.

#### Pectoral girdle and fin (Figs 5, 6, 16, 23, 27, 29, 30)

The pectoral girdles are well-preserved and comprise the clavicle, the extracleithrum, the cleithrum, the scapulocoracoid, the anocleithrum, as well as an interclavicle. No postemporal and supracleithrum bones have been identified. Ornamentation is limited to a few sparse tubercules on the lateral surfaces of the clavicle, cleithrum and extracleithrum. Only the properly ossified elements from the girdle and the fin rays have been retrieved, the rest of the endoskeletal bones (mesomeres and radials) of the fin was likely cartilaginous.

*Interclavicle*. The interclavicle (Icla) is a small and elongate median ossification, with an hour-glass shape, and gently curved dorsally. The dorsal surface is flat, smooth, and slightly concave towards the rear whereas the ventral surface carries a median ridge. The anterior portion is wider than the rest of the bone and the posterior margin is shaped as a two-pronged fork. The interclavicle is not ornamented.

*Clavicle*. The clavicle (Cla) is overall well-preserved. It can be divided into two distinct portions: a large dorso-lateral section, overlapping the ventral edge of the cleithrum and of the extracleithrum, and a dorso-ventrally flattened antero-ventral flange. The dorso-lateral section displays a blade-like pointed process embracing a groove present in the antero-lateral portion of the cleithrum and overlapping it, while the internal surface is strongly concave and in the form of a triangular funnel. The anterior portion shows a prominent medial expansion with a straight median margin for the suture with the adjoining clavicle and is connected to the dorso-lateral section by a narrow bony bridge.

*Cleithrum*. The cleithrum (Cl) is completely preserved. It is wider anteriorly and postero-dorsally, with a narrower mid-section. The postero-dorsal expansion shows a roughly quadrangular outline dorsally, while the ventral projection is acute and blade-like, fitting into the internal groove of the clavicle. The external surface is slightly convex, except for the anterior narrow groove that accommodates the clavicle, and ornamented by small unevenly distributed tubercles. The internal surface is concave in the form of a triangular gutter associated with a prominent ridge across the anterior leading margin, probably housing a large cartilaginous blade supporting the scapulocoracoid.

*Extracleithrum*. The extracleithrum (Ecl), a unique feature of coelacanths among osteichthyans, is a narrow and elongate bone. It is externally convex and internally concave. The ventral edge is overlapped by the clavicle, abutting a ventro-lateral buttress, while the major part of the bone fits in a postero-lateral depression of the cleithrum. The postero-dorsal edge displays a small depression concomitant with a similar depression in the posterior margin of the cleithrum. As for the cleithrum, ornamentation consist of small tubercles located close to the postero-lateral margin.

*Anocleithrum*. The anocleithrum (Acl) has been retrieved in each specimen. However, it shows an interesting intraspecific variability and asymmetry. In MHNG GEPI V5787 it is a blade-like, unornamented element, with a wide ventral section and a pointed dorsal section, whereas in MHNG GEPI V5788 the right anocleithrum displays a bifurcated dorsal projection, different from the simple projection of the left anocleithrum. Despite this variation, in each specimen the external surface is slightly concave in the ventral portion for the articulation with the dorsal margin of the cleithrum while the internal surface displays a gutter running dorso-ventrally probably for muscular attachments from the branchial arches.

*Scapulocoracoid*. The articulatory portion of the scapulocoracoid (Scc) is completely preserved, although lightly ossified with extremely thin periosteal walls resulting in an almost tubular element. The base is quadrangular, slightly narrowing antero-ventrally. The medial columnar section is dorso-posteriorly inclined and the ovoid proximal edge would have housed the probably convex cartilaginous articulatory head for the first mesomere of the pectoral fin (humerus). There are no traces of perforations on the outer surface of the bone. No other elements from the pectoral fin endoskeleton have been retrieved, suggesting that they were certainly entirely cartilaginous.

*Fin rays*. The pectoral fin carries around 20 ossified fin rays (lepidotrichia) (Lep). Each lepidotrichium is composed of two symmetrical hemirays (hemilepidotrichia) forming a bifurcated proximal end and embracing the most distal portion of the unpreserved fin´s radials. The proximal region of each ray is long and unjointed, whereas the distal portion is richly segmented, formed by numerous small bony segments of equal size. The first two rays from the preaxial (ventral) side of the fin are the smallest of the series and do not show any distal segmentation. The next rays become abruptly elongate and then decrease in size towards the postaxial (dorsal) side. The length of the unjointed proximal segment appears to vary from preaxial to medial relative to the distal segmented portion of the ray. However, the distal region of the fin web is greatly disorganized and thus it is complicated to identify the longest rays or any significant pattern of size gradation.

#### Pelvic girdle and fin (Figs 16, 27, 29)

The pelvic fins lie in an abdominal position and appear to be located between the first and second dorsal fins. As for the pectoral fins, only the girdle and several fin rays have been retrieved, the rest of the endoskeletal components of fin was certainly unossified.

*Pelvic girdle*. The pelvic girdle (p.b) is a stout element organized into three main portions: the anterior process, the medial process, and the posterior portion. Both left and right pelvic girdles have been found separated from each other in MHNG GEPI V5788, revealing that the hemi-girdles were not fused in the midline. The anterior process consists of a long and flattened projection composed of two apophyses connected by a thin bony membrane. The lateral apophysis is thinner and more elongate than the medial one. The medial process is a short and rectangular expansion, perpendicularly arranged relative to the main axis of the girdle. The median border is simple, without digitations, and would have connected with the opposite girdle through ligaments. The posterior part of the girdle displays a triangular expansion, with an unfinished posterior margin, which was certainly covered in cartilage forming the articular socket (or acetabulum) for the first axial element of the fin (femur). There is no evidence of a bony lateral expansion.

*Fin rays*. The pelvic fins carry around 25 ossified fin rays (lepidotrichia) (Lep). As in the pectoral fins (see above), the proximal end of each lepidotrichium is formed of two symmetrical hemirays. The lepidotrichia display a long proximal segment and numerous smaller, equidimensional segments distally. The first two rays from the preaxial (lateral) side of the fin are small and unjointed. The next rays drastically increase in size, with the longest being the L7-9, and then gradually decrease in length toward the postaxial (medial) side. The length of the unjointed proximal segment decreases from lateral to medial relative to the distal segmented portion of the rays (from 2/3 preaxially to 1/2 of the ray´s total length postaxially).

#### Vertebral column and median fins (Figs 15, 16, 25, 26, 29)

Almost the complete axial skeleton has been retrieved, comprising numerous vertebrae and the bony bases of the median fins (both dorsal and anal fins), caudal fin radials, as well as associated bony rays (lepidotrichia).

#### Vertebral column

*Vertebrae*. Approximately 63 vertebral ossifications have been reconstructed. They consist of around 51 lightly ossified neural and 12 haemal arches and spines. All vertebrae (Ve) are composed of a fused neural arch and spine, without traces of suture. The neural arches of the most anterior vertebrae, immediately behind the head, are larger and slightly more ossified than the rest, framing a broad notochord. The dorsal portion of the neural arches is slightly expanded into thin anterior and posterior flanges where the neural spine joins the neural arch. As for the supraoccipital, a foramen for the supradorsal ligament is visible in the dorsal portion of the neural arch at the base of the neural spine. The neural spines are posteriorly inclined and variable in size and shape. The spine in Ve3 is larger and shorter than the longer and more slender spines from more posterior vertebrae (e.g., Ve16-18), which increase in size throughout the vertebral column from anterior to posterior. The neural spines are somewhat shorter from Ve8 to Ve14, probably to accommodate the broad first dorsal fin base. There are no ossified vertebral centra. Haemal arches have been found in the caudal region, behind the anal fin. They consist of haemal arches and fused median haemal spines (h.a+h.s), ventral mirror images of the neural arches and spines dorsally. The longest haemal spines are located in the anterior part of the caudal region and progressively decrease in size posteriorly. There is no evidence of epineural (supraneural) nor epihaemal (suprahaemal) spines, the neural and haemal spines would have directly articulated with the radials distally.

*Ribs*. 15 pairs of ossified pleural ribs (plr) have been retrieved, bilaterally arranged, occupying the abdominal cavity. They consist of slender bony rods, with a slightly enlarged proximal portion, and pointy tapering distal ends. They are postero-ventrally oriented, gently curved inwards, and laterally framing an ossified lung. The most anterior ribs have been retrieved at the level of the first dorsal fin and appear shorter than the more posterior ones, which progressively increase in length towards the rear. The longest ribs are located immediately anterior to the first preserved haemal arches and spines and surround the most posterior portion of the lung.

#### Median fins

*Dorsal fins*. Both dorsal fins are preserved. The first dorsal fin is represented by a roughly triangular basal plate-like element (d1.b), displaying two ridges anteriorly converging at the ossification centre of the plate. The second dorsal fin is also represented by a basal plate (d2.b), strongly bifurcated anteriorly, but no distal axial mesomeres and radials have been retrieved, likely due to their cartilaginous state. There is also no evidence of a postero-ventral process as in *Laugia* (ossified) or *Latimeria* (cartilaginous) [2]. The fin rays have been heavily damaged during preparation. The first dorsal fin carries at least 10 stout fin rays of which the first one is significantly smaller, while the second dorsal fin carries approximately 13 rays, solely represented by their forked proximal edges in MHNG GEPI V5788 as the rest of the rays has been eroded.

*Anal fin*. The anal fin in MHNG GEPI V5788 also displays an anteriorly bifurcated basal plate (a.b), similar to the second dorsal fin, but in the anal fin these are symmetrically arranged lateral projections, posteriorly connected to the flattened and vertically oriented body of the plate. The posterior portion shows a small descending ventral expansion. As for the second dorsal fin, no axial mesomeres nor any putatively cartilaginous ventral or dorsal processes have been retrieved. The anal fin carries approximately 15 fin rays.

*Caudal fin*. The caudal fin is relatively short and is composed of two symmetrical (epi- and hypochordal) lobes, roughly equal in size, separated by a gap for the middle supplementary lobe. The tail of MHNG GEPI V5788 has a circular contour but this may be due to an incomplete preservation or excessive preparation of the specimen. The supplementary lobe could not be clearly reconstructed because of the delicate condition of the fin rays and the lack of ossified radials. However, based on the overall preservation of the tail, it doesn´t appear that the supplementary lobe would have projected much further posteriorly but instead remained within the posterior boundaries of the dorsal and ventral lobes. It is difficult to correctly count the number of radials and fin rays forming the lobes due to the excessive preparation of the tail, but the dorsal (epichordal) lobe of MHNG GEPI V5788 displays around 15 fin rays, showing a one-to-one relationship with the radials.

*Radials*. Dorsal and ventral radials (ra) associated with the neural and haemal spines respectively are present in the caudal region. They are elongate tubular elements, only perichondrally ossified, with a narrow middle shaft and only slightly larger proximal and distal extremities. In the caudal region, each preserved radial articulates with a neural or haemal spine proximally and a single lepidotrichium distally.

*Fin rays*. Ossified fin rays (lepidotrichia) (Lep) are present in the first and second dorsal, anal and caudal fins. They are stout osseous elements composed of two symmetrical hemirays, contralaterally arranged on both sides of the most distal elements of the fin endoskeleton (i.e., the broad basal plate of the first dorsal fin, the unpreserved mesomeres from the second dorsal and anal fins, and the lightly ossified radials of the caudal fin), resulting in a two-pronged proximal edge. The lepidotrichia are segmented (or jointed) with a long proximal segment and numerous smaller, equidimensional segments more distally, except the first one or two most anterior rays in every fin. The lepidotrichia of the first dorsal fin carry numerous pointed tubercles on the leading edge, both on the distal portion of the long proximal segment and on the small segments distally. No evidence of distal bifurcation has been found in any fin.

#### Scales (Figs 17 and 28)

The axial skeleton of MHNG GEPI V5787 and MHNG GEPI V5788 is surrounded by clusters of small scales with different degrees of preservation. Only a few scales have been modelled: in MHNG GEPI V5787, two partially preserved scales from the ventral region close to the pelvic fin, and one complete from the antero-dorsal portion of the trunk; in MHNG GEPI V5788, four nicely preserved scales from the left flank of the trunk, two at the level of the lateral line and the other two located more dorsally or ventrally. The shape of the scales varies from ovoidal to slightly quadrangular with some degree of antero-posterior elongation and a rounded anterior margin. All scales display ornamentation. The external surface is slightly convex and is composed of two areas: an overlapped area (or anterior field) that appears smooth in the 3D renders, and an exposed ornamented area (or posterior field). The overlapped area occupies between 1/2 to 2/3 of the external surface of the scales but it can only be partially reconstructed, hinting at the extreme thinness of the anterior field. The ornamentation of the exposed area consists of numerous large, slightly antero-posteriorly elongate tubercles, apparently homogenously distributed across the exposed area but impossible to investigate histologically. Several scales present subtle differences in the size and shape of the tubercles, revealing a possible variation of the ornamentation across the body. The internal surface is flat to slightly concave but it is too badly preserved to be reconstructed and described, possibly revealing a partially unossified basal layer. Some large scales carry the main lateral line sensory canal (l.l.c). They bear a canal consisting of a simple tube that opens via two small symmetrical pores (p.l.l) on the posterior field of the scale.

#### Ossified lung (Figs 2, 14, 24, 29)

A calcified elongated mass occupying the abdominal cavity occurs in MHNG GEPI V5787 and MHNG GEPI V5788 and we consider it here as an ossified lung. The modelled elements correspond to the ossified plates surrounding a single lung, as known in several other fossil coelacanths (e.g., *Allenypterus*, *Piveteauia*, *Coccoderma*, *Axelrodichthys*, *Macropoma*, *Swenzia)* [43–45]. Based on the distribution of the ossified plates, the lung would have stretched from the rear of the skull to the anal fin. The presence of three distinctive regions suggests that the lung was organized into three chambers separated by constrictions. The first chamber, or lobe, bears two dorsally projected crests, parallel to each other, histologically very different from the rest of the ossified plates of the lung as they appear in synchrotron microCT sections (Fig 2). The function of the two crests can be only speculated at this stage, maybe part of a larger, mainly cartilaginous connection between lung and notochord. Further investigations are currently underway.

### Phylogenetic analysis

**Fig 31:**
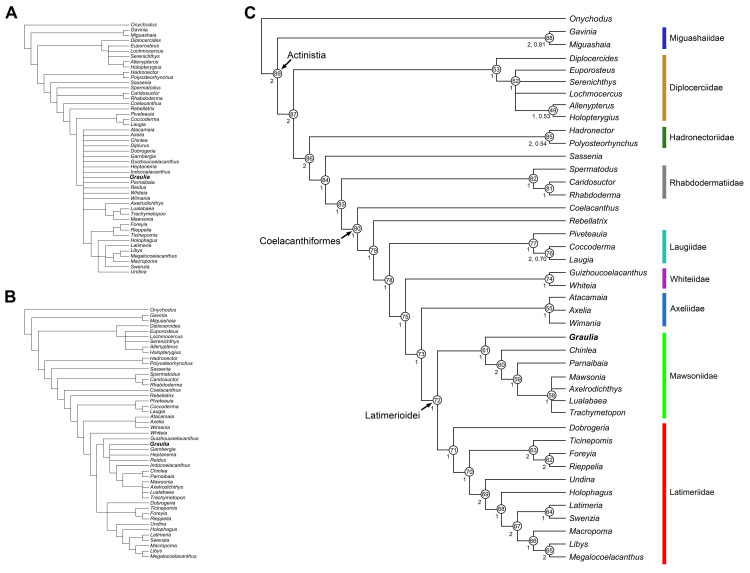
Phylogenies of Actinistia including *Graulia branchiodonta* gen. et sp. nov. (A) strict consensus tree of 33165 equally parsimonious trees (length = 323; consistency index = 0.38; retention index = 0.68) obtained from the first phylogenetic analysis. (B) strict consensus tree of 1890 equally parsimonious trees (length = 311; consistency index = 0.39; retention index = 0.69) obtained from the second phylogenetic analysis performed without the taxon *Diplurus*. (C) strict consensus tree of 75 equally parsimonious trees (length = 307; consistency index = 0.40; retention index = 0.68) obtained from the third phylogenetic analysis performed without the taxa *Diplurus*, *Garnbergia*, *Heptanema*, *Indocoealacanthus*, *Reidus*. Integers indicate the Bremer decay indices. Bootstrap values are indicated after the Bremer decay indices if greater than 50%.

A series of phylogenetic analyses was performed to clarify the phylogenetic placement of *Graulia branchiodonta*. The first phylogenetic analysis, including the whole dataset of 48 taxa and 111 characters (S1 Table) (S1 Appendix), resulted in a poorly resolved strict consensus tree from 33165 equally parsimonious trees (length = 323; consistency index = 0.38; retention index = 0.68) (Fig 31A). Within such tree, *Graulia* was in polytomy with *Atacamaia*, *Axelia*, *Chinlea*, *Diplurus*, *Dobrogeria*, *Garnbergia*, *Guizhoucoelacanthus*, *Heptanema*, *Indocoelacanthus*, *Parnaiba*, *Reidus*, *Whiteia*, *Wimania*, (*Axelrodichthys* + *Lualabea* + *Trachymetopon* + *Mawsonia*) and the Latimeriidae. Considering that our data matrix was almost identical to the one included in Ferrante and Cavin [7] that resulted in a much better resolved tree, the only modifications being the addition of *Graulia* and the removal of character 66 (Subopercular branch of the mandibular sensory canal present/absent), the phylogenetic disturbance was related to the inclusion of *Graulia* and/or the removal of the character. A second phylogenetic analysis performed excluding *Graulia* resulted in a strict consensus tree from 135 equally parsimonious trees (length = 309; consistency index = 0.39; retention index = 0.70) with the same topology as the strict consensus tree from Ferrante and Cavin [7], confirming that *Graulia* was responsible for the polytomies. Such tree is not shown here as it represented just a test for the quality of the phylogeny from Ferrante and Cavin [7]. To identify which other taxa could be responsible for the polytomies, we performed 16 different phylogenetic analyses excluding one taxon from the polytomy at the time.

The taxon creating more phylogenetic disturbance was *Diplurus*, as upon its removal we obtained a better resolved strict consensus tree of 1890 equally parsimonious trees (length = 311; consistency index = 0.39; retention index = 0.69) (Fig 31B) with *Graulia* placed at the base of the Mawsoniidae in a polytomy with *Garnbergia*, *Heptanema* and *Reidus*. *Diplurus* is a well-known taxon with a history of conflicting phylogenetic placements, being recovered as a basal Mawsoniidae [7, 46] but also as a Latimeriidae [4, 5, 15]. To further investigate the position of *Graulia* within the family Mawsoniidae, we performed a phylogenetic analysis excluding *Diplurus*, *Garnbergia*, *Heptanema*, *Indocoelacanthus* and *Reidus*. The rationale for excluding the latter four taxa was related to previous studies defining them as phylogenetically unstable [4, 24]. The analysis produced a strict consensus tree of 75 equally parsimonious trees (length = 307; consistency index = 0.40; retention index = 0.68) (Fig 31C) with *Graulia* placed as a the most basal Mawsoniidae. We retained this tree as our final tree. The removal of the most phylogenetically unstable taxa is common practice in phylogenetic studies of coelacanths [4, 24] due to the presence of very poorly known taxa that generates uncertainty for the impossibility to code most of the character states, or taxa that are well known but generate uncertainty in a context of poorly known taxa. Bremer and Bootstrap values are shown in the final tree (Fig 31C) to highlight the nodes with the strongest support, but were not used as a parameter to retain the tree as most branches should have been collapsed according to their poor statistical support (this is common practice in coelacanth phylogenies due to the very low statistical support).

Despite the removal of 5 taxa and 1 character and the addition of *Graulia* the strict consensus tree closely resembles the one from Ferrante and Cavin [7], with the same topology supporting 9 coelacanth families: Miguashaiidae, Diplocerciidae, Hadronectoridae, Rhabdodermatidae, Laugiidae, Whiteiidae, Axeliidae, Mawsoniidae and Latimeriidae. The only notable differences concern the clade corresponding to Mawsoniidae, including *Graulia* + (*Chinlea* + (*Parnaiba* + (*Mawsonia*, *Axelrodichthys*, *Lualabea* and *Trachymetopon*))), which is a smaller clade here compared to Ferrante and Cavin [7] as we removed *Diplurus*, *Heptanema*, *Indocoelacanthus* and *Reidus* from the data matrix. The family Mawsoniidae is supported by three unambiguous synapomorphies: the presence of extrascapulars forming part of the skull roof (consistency index: 1.000) (character 19{1}), the presence of ventrally directed pores of the mandibular sensory canal on the splenial (consistency index: 0.500) (character 67{1}; 68 in Ferrante and Cavin [7]) and the presence of ossified ribs (consistency index: 1.000) (character 95{1}, 96 in Ferrante and Cavin [7]). *Graulia* is the only Mawsoniidae presenting ossified ribs at the level of the anterior abdominal region, while the other taxa have them in the posterior abdominal region. The clade is supported also by five ambiguous synapomorphies: the extrascapulars sutured to the postparietals (consistency index: 0.200) (character 18{0}), the skull roof ornamented with rugosities and striae (consistency index: 0.333) (character 28{2}), the cheek ornamented with round tubercles (consistency index: 0.333) (character 54{1}), the presence of a dentary sensory pore (consistency index: 0.500) (66{1}; 67 in Ferrante and Cavin [7]) and the absence of markings for the oral pit line on the angular (consistency index: 0.250) (character 68{1}; 69 in Ferrante and Cavin [7]).

The presence of *Graulia* changed some of the synapomorphies supporting Mawsoniidae when compared to the results of Ferrante and Cavin [7], where it was unambiguously supported by the ornamentation of rugosities and striae on the skull roof and cheek (character 28{2}) (character 54{1}), the dentary with a prominent lateral swelling (character 59{1}) and the presence of ossified ribs (character 95{1}, 96 in Ferrante and Cavin [7]). In *Graulia* the ornamentation consists of ovoidal tubercles, and the lateral swelling of the dentary is not so pronounced, producing ambiguity in the related synapomorphies. Mawsoniidae was also ambiguously supported in Ferrante and Cavin [7] by the absence of a supratemporal descending process (character 16{0}), the pit lines marking the anterior third of the postparietals (character 27{1}) and the presence of a dentary sensory pore (character 67{1}; 67 in Ferrante and Cavin [7]). In *Graulia* there is a descending process of the supratemporal that invalidates the synapomorphy for Mawsoniidae. Furthermore, the synapomorphy related to the presence of a dentary sensory pore changed from ambiguous in Ferrante and Cavin [7] to unambiguous in our analysis, but such change was related to the removal of *Diplurus*, *Heptanema*, *Indocoealacanthus* and *Reidus*.

*Graulia* was retrieved as sister group of other Mawsoniidae that was supported by 5 unambiguous synapomophies: the absence of the preorbital (consistency index: 0.333) (character 13{0}), the postorbital spanning the intracranial joint (consistency index: 0.500) (character 34{1}), the absence of a suboperculum (consistency index: 0.200) (character 41{0}), a large orbital space not occupied entirely by the eye (consistency index: 0.333) (character 55{1}) and the differentiated ornamentation of the scales (consistency index: 0.333) (character 111{1}; 112 in Ferrante and Cavin [7]). The Mawsoniidae minus *Graulia* was also supported by 5 ambiguous synapomophies: five or more premaxillary teeth (consistency index: 0.200) (character 4{0}), the absence of a descending process on the supratemporal (consistency index: 0.500) (character 16{1}), the pit lines marking the posterior half the postparietals (consistency index: 0.250) (character 27{0}), the ornamentation of the cheek consisting of rugosities and striae (consistency index: 0.333) (character 54{1}) and the temporal excavation lined with bone (consistency index: 0.250) (character 73{1}; 74 in Ferrante and Cavin [7]).

## Discussion

### Anatomical comparisons and systematic identification

**Fig 32:**
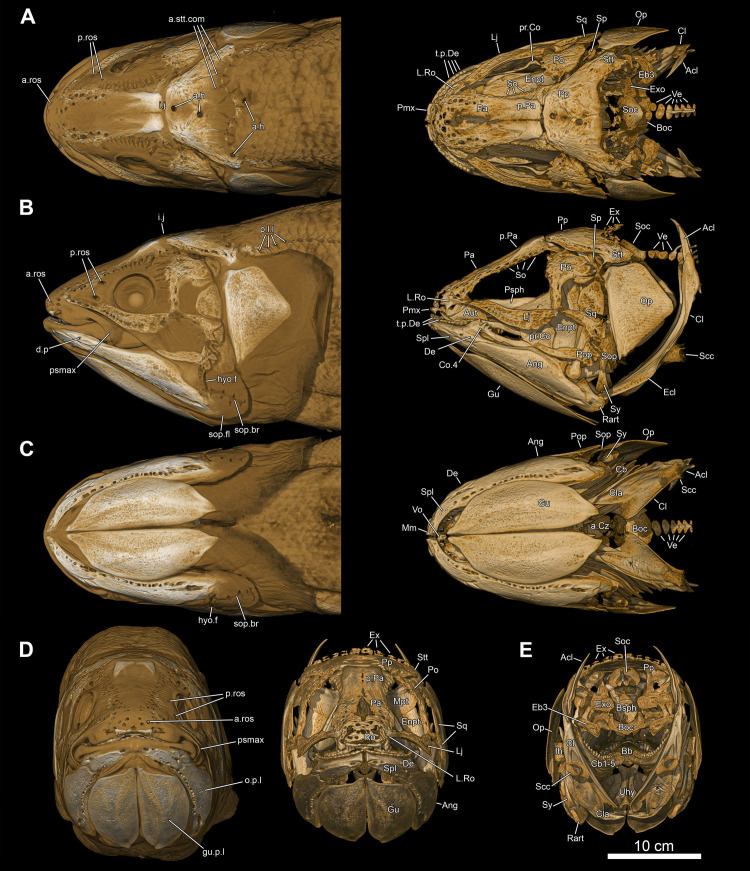
*L*. *chalumnae*, MHNG 1080.070, male, 116 cm TL. Volume-rendered 3D models of the head, up to the posterior end of the pectoral girdle, obtained from microCT. The density gradient is colorized from brown to white in ascending order. (A) dorsal view of entire head (left) and isolated skeleton (right). (B) lateral view of entire head (left) and isolated skeleton (right). (C) ventral view of entire head (left) and isolated skeleton (right). (D) anterior view of entire head (left) and isolated skeleton (right). (E) posterior view of cranial skeleton and pectoral girdle. A high-resolution version (600 dpi) of this figure can be downloaded from Figshare (DOI: 10.6084/m9.figshare.26166943).

**Fig 33:**
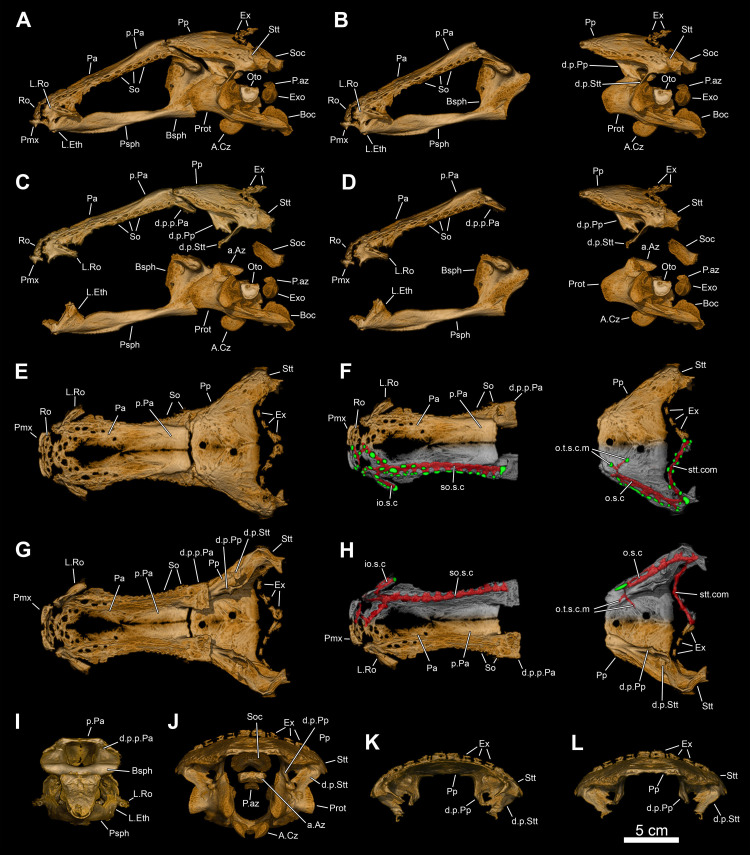
*L*. *chalumnae*, MHNG 1080.070, male, 116 cm TL. Volume-rendered 3D models of the braincase, obtained from microCT. The density gradient is colorized from brown to white in ascending order. The red casts of the lateral line cavities are shown in transparency, with the pores labelled green. (A) lateral view of the articulated braincase. (B) lateral view of the braincase with ethmosphenoid and otoccipital portions spaced apart. (C) lateral view of the braincase with the skull roof detached from the neurocranium. (D) lateral view of the braincase with ethmosphenoid and otoccipital portions spaced apart and the skull roof detached from the neurocranium. (E) dorsal view of the skull roof. (F) dorsal view of the skull roof with the parietonasal and postparietal shields spaced apart. (G) ventral view of the skull roof. (H) ventral view of the skull roof with the parietonasal and postparietal shields spaced apart. (I) posterior view of the ethmosphenoid portion of the braincase. (J) anterior view of the otoccipital portion of the braincase. (K) posterior view of the postparietal shield. (L) anterior view of the postparietal shield. A high-resolution version (600 dpi) of this figure can be downloaded from Figshare (DOI: 10.6084/m9.figshare.26166943).

**Fig 34:**
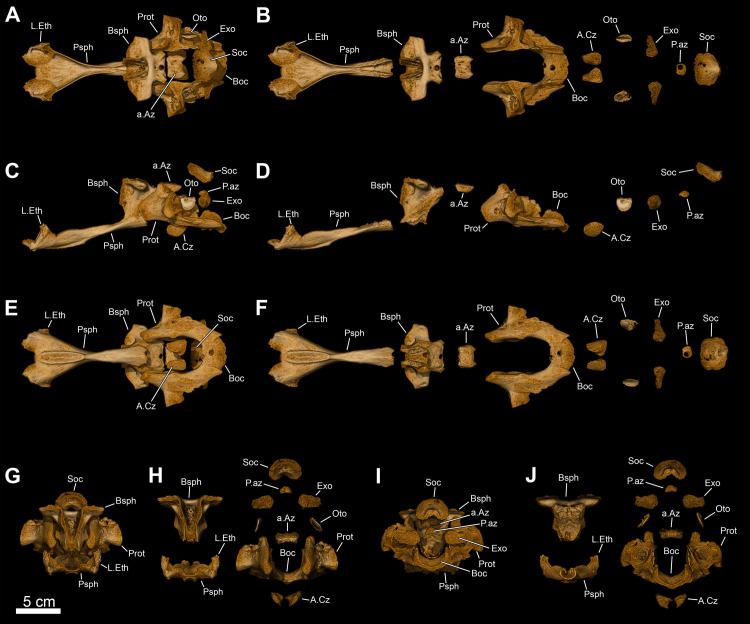
*L*. *chalumnae*, MHNG 1080.070, male, 116 cm TL. Volume-rendered 3D models of the neurocranium and parasphenoid, obtained from microCT. The density gradient is colorized from brown to white in ascending order. (A) dorsal view of neurocranium. (B) dorsal view of neurocranium with skeletal elements spaced apart. (C) lateral view of neurocranium. (D) lateral view of neurocranium with skeletal elements spaced apart. (E) ventral view of neurocranium. (F) ventral view of neurocranium with skeletal elements spaced apart. (G) anterior view of neurocranium. (H) anterior view of neurocranium with skeletal elements spaced apart. (I) posterior view of neurocranium. (J) posterior view of neurocranium with skeletal elements spaced apart. A high-resolution version (600 dpi) of this figure can be downloaded from Figshare (DOI: 10.6084/m9.figshare.26166943).

**Fig 35:**
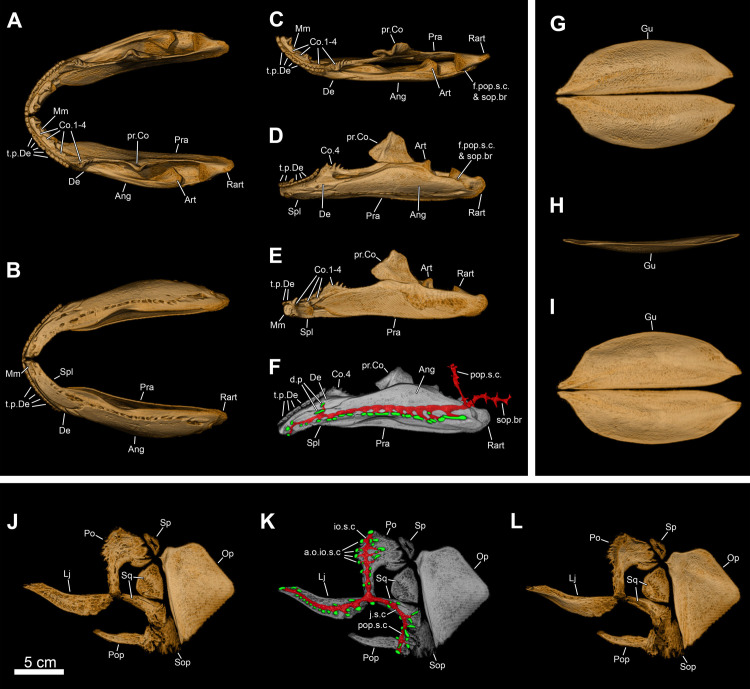
*L*. *chalumnae*, MHNG 1080.070, male, 116 cm TL. Volume-rendered 3D models of the lower jaw, cheek bones and gular plates, obtained from microCT. The density gradient is colorized from brown to white in ascending order. The red casts of the lateral line cavities are shown in transparency, with the pores labelled green. (A) dorsal view of lower jaw. (B) ventral view of lower jaw. (C) dorsolateral view of lower jaw, left half. (D) lateral view of lower jaw, left half. (E) medial view of lower jaw, left half, flipped horizontally. (F) lateroventral view of lower jaw with casts of the sensory canals. (G) ventral view of gular plates. (H) lateral view of gular plates. (I) dorsal view of gular plates. (J) lateral view of cheek bones. (K) lateral view of check bones with casts of the sensory canals. (L) medial view of cheek bones, flipped horizontally. A high-resolution version (600 dpi) of this figure can be downloaded from Figshare (DOI: 10.6084/m9.figshare.26166943).

**Fig 36:**
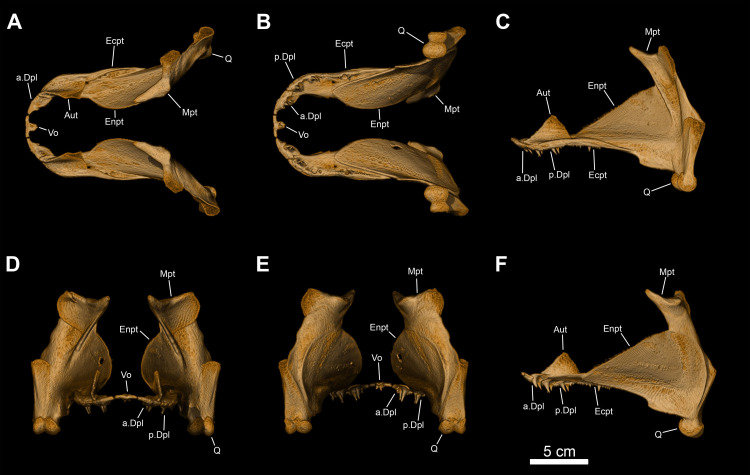
*L*. *chalumnae*, MHNG 1080.070, male, 116 cm TL. Volume-rendered 3D models of the palate, obtained from microCT. The density gradient is colorized from brown to white in ascending order. (A) dorsal view of palate. (B) ventral view of palate. (C) lateral view of palate, left half. (D) anterior view of palate. (E) posterior view of palate. (F) medial view of palate, left half, flipped horizontally. A high-resolution version (600 dpi) of this figure can be downloaded from Figshare (DOI: 10.6084/m9.figshare.26166943).

**Fig 37:**
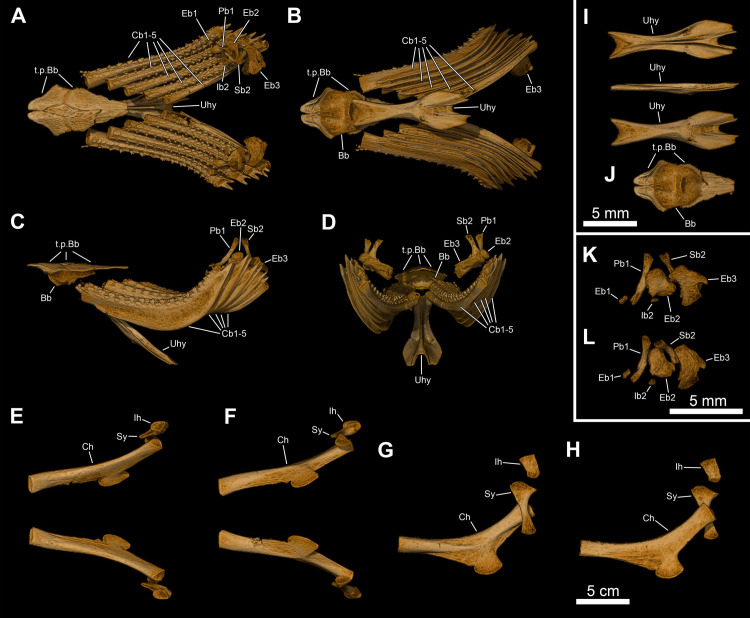
*L*. *chalumnae*, MHNG 1080.070, male, 116 cm TL. Volume-rendered 3D models of the hyobranchial skeleton, obtained from microCT. The density gradient is colorized from brown to white in ascending order. (A) dorsal view of gill arches, basibranchial and urohyal. (B) ventral view of gill arches, basibranchial and urohyal. (C) lateral view of gill arches, basibranchial and urohyal. (D) posterior view of gill arches, basibranchial and urohyal. (E) dorsal view of hyoid arch. (F) ventral view of hyoid arch. (G) lateroventral view of left hyoid arch. (H) dorsomedial view of left hyoid arch, flipped horizontally. (I) from top to bottom, urohyal in dorsal, lateral and ventral view. (J) ventral view of basibranchial. (K) lateral view of upper gill arches. (L) medial view of upper gill arches. A high-resolution version (600 dpi) of this figure can be downloaded from Figshare (DOI: 10.6084/m9.figshare.26166943).

**Fig 38:**
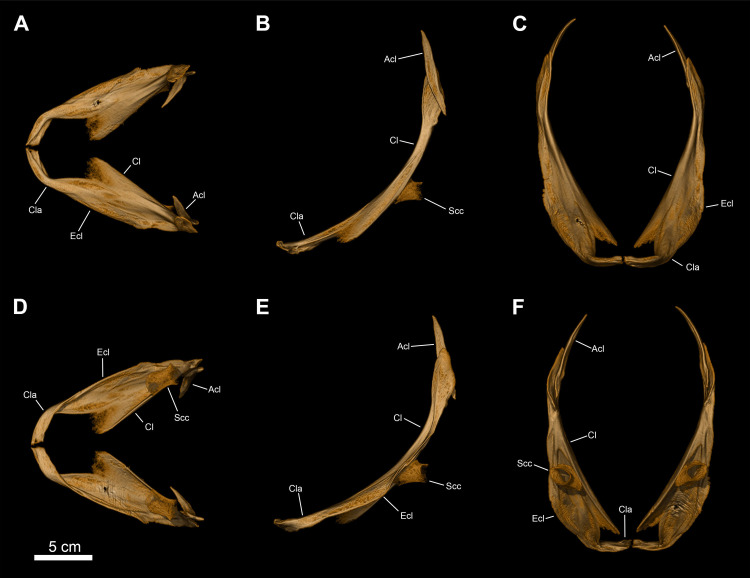
*L*. *chalumnae*, MHNG 1080.070, male, 116 cm TL. Volume-rendered 3D models of the pectoral girdle, obtained from microCT. The density gradient is colorized from brown to white in ascending order. (A) dorsal view. (B) medial view (left half only). (C) anterior view. (D) ventral view. (E) lateral view (left half only). (F) posterior view. A high-resolution version (600 dpi) of this figure can be downloaded from Figshare (DOI: 10.6084/m9.figshare.26166943).

Despite its excellent quality of preservation and the occurrence of two relatively complete specimens, the phylogenetic position and affinities of *Graulia branchiodonta* with other coelacanths has been difficult to decipher, mainly due to the phylogenetic instabilities produced by other Mesozoic taxa (e.g., *Diplurus*, *Heptanema*, *Indocoealacanthus* and *Reidus*). The position of *Graulia* as an early mawsoniid is supported by several diagnostic features such as the presence of extrascapulars forming part of the skull roof, the presence of ventrally directed pores of the mandibular sensory canal on the splenial, and the presence of ossified ribs. However, *Graulia* also displays some characters typically found outside of the Mawsoniidae, such as the presence of preorbital and a subopercular bones, the presence of postparietal and supratemporal descending processes, and the presence on the postparietals of anterior branches of the supratemporal commissure. This mix of features in a Triassic coelacanth highlights the diversity of character combinations during the early Mesozoic at the dawn of the diversification of the Latimerioidei and the establishment of the diagnostic traits of its two families: the Latimeriidae and the Mawsoniidae. The features of each anatomical module of *Graulia* are discussed in the following paragraphs via a thorough comparison with other coelacanth taxa. For a direct comparison with the extant species, a series of plates illustrating the cranial anatomy of *L*. *chalumnae* are shown in Figs 32–38.

#### Skull roof (Figs 5–7, 20, 30; *L*. *chalumnae* Figs 32, 33)

*Graulia branchiodonta* displays the classic coelacanth condition of a dermal skull roof divided by the intracranial joint into parietonasal and postparietal shields, with the exception of the fused skull roof of *Foreyia* and *Rieppelia* [6, 7]. The parietonasal shield is slightly longer than the postparietal shield (ratio of 1.35) as in many Mesozoic and Recent Latimerioidei (e.g., *Axelrodichthys*, *Mawsonia*, *Diplurus*, *Swenzia* and *Latimeria*), but also as in some Paleozoic forms like *Allenypterus*, *Hadronector*, and *Polyosterynchus* [2]. Paired parietals as displayed in *Graulia branchiodonta* are common among coelacanths, with the exceptions of *Miguashaia*, *Atacamaia*, *Axelia*, *Laugia* and *Wimania*. The joint between the posterior parietal and postparietal bones is straight and simple, similar to that of many coelacanths from *Miguashaia* to *Latimeria*, but different from the digitated margins of certain forms like *Spermatodus* and *Atacamaia* [47]. The mosaic of rostral bones, clearly separated from each other, differs from the consolidated snouts of *Laugia*, *Macropoma*, *Megalocoelacanthus* and *Swenzia*. The premaxilla carries between three and four large teeth, a reduced number compared with earlier coelacanths like *Miguashaia* and *Spermatodus*, but more similar to the condition of latimeriids with four or less teeth like *Ticinepomis*, *Macropoma* and *Latimeria* than to mawsoniids with five or more teeth (with the exception of *Axelrodichthys*). The supraorbital series is composed of numerous small bones carrying the supraorbital sensory canal, which opens through pores at the sutural contact between bones like in *Diplurus* but different from the many pores in *Whiteia* and *Ticinepomis*, or the continuous groove of *Foreyia* [6].

The occurrence of a preorbital bone in *Graulia branchiodonta* is variable across coelacanths. The preorbital is primitively present in many Palaeozoic and Mesozoic coelacanths (e.g., *Diplocercides*, *Lochmocercus*, *Spermatodus*, *Hadronector*, *Caridosuctor*, *Axelia*, *Sassenia*, *Whiteia*, *Laugia* and the Ticinepominae) but it disappears in *Coelacanthus* and many Mesozoic taxa like *Undina*, *Holophagus*, *Macropoma*, *Lybis*, *Macropomoides*, *Diplurus*, and *Latimeria*. Its absence has even been considered a synapomorphy of the derived Mawsoniidae (e.g., *Chinlea Axelrodicthys*, *Mawsonia*) [7] but could also characterize derived latimeriids (e.g., *Undina*, *Holophagus*, *Macropoma*, *Libys*, and *Latimeria*) as it is primitively present in the Ticinepominae (e.g., *Foreyia*, *Rieppelia* and *Ticinepomis*). Forey [46] stated that, when present, the preorbital is always perforated by the posterior opening of the rostral organ, however no pores occur in *Ticinepomis* and in *Graulia branchiodonta*. The condition of *Graulia branchiodonta* is reminiscent of *Laugia* in which the openings of the rostral organ form notches on the postero-ventral margin of the preorbital [2] as opposed to pores enclosed within the body of the bone. It may as well be that the ventral processes of the preorbital in *Graulia branchiodonta* simple support the canal of the rostral organ towards its posterior pore that would be located nearby.

The postparietal shield of *Graulia branchiodonta* is composed of the large postparietals, the postero-laterally arranged tabulars (supratemporals) and the extrascapular series forming the posterior margin of the skull roof. The term ‘tabular’ may be more appropriate than the commonly used ´supratemporal´ in coelacanth literature [2, 40, 48] to describe the posterior-most bone carrying the otic lateral-line canal and laterally flanking the postparietals. Primitively for sarcopterygians, the postparietals are laterally flanked by two lateral-line carrying bones: the supratemporal (anterior) and the tabular (posterior), with the tabular being located immediately anterior to the lateral extrascapular. *Miguashaia* (and possibly *Gavinia*) displays this primitive condition for coelacanths, with a complete series of lateral line canal-bearing bones lateral to the postparietals, including from anterior to posterior an intertemporal, supratemporal, tabular and lateral extrascapular [13]. The supratemporal was probably lost or fused to the anterior portion of the postparietal early during coelacanth history [1], leaving the tabular as the only postero-laterally-flanking bone of the postparietal shield. Posteriorly, the position of the posterior margin of the tabular (supratemporal) is also variable in coelacanths: either it ends at the same level as the posterior margin of the postparietal (as in *Diplocercides*, *Allenypterus*, *Rhabdoderma*, or *Laugia*), or it extends beyond the posterior margin of the postparietal and extrascapular series (as in *Coelacanthus*, *Whiteia*, *Diplurus*, *Axelrodichthys*, *Mawsonia*, *Macropoma* and *Latimeria*). The latter condition is assumed to result from the fusion of the lateralmost extrascapular with the tabular (supratemporal), as in these taxa the otic canal and supratemporal commissure join within the tabular (supratemporal), resulting in an embayed posterior margin of the skull roof [2, 46].

The postparietals reveal numerous interesting phylogenetic characters as highlighted in *Graulia branchiodonta*. Medial branches of the otic canal are well developed, as in many post-Carboniferous coelacanths (e.g., *Spermatodus*, *Laugia*, *Coccoderma*, *Whiteia*, *Macropoma*, *Mawsonia* and *Latimeria*). Posteriorly, shallow grooves reveal the path of anterior branches of the supratemporal commissure spreading from the extrascapular series onto the postparietals. These structures were previously known only in the Latimeriidae (e.g., *Macropoma*, *Swenzia* and *Latimeria*, and probably in *Holophagus*) [2, 49], while they never occur in classic Mawsoniidae (e.g., *Axelrodichthys*, *Chinlea*, *Diplurus* and *Mawsonia*). Their occurrence in an early mawsoniid like *Graulia branchiodonta* may indicate their primitive presence in the Latimerioidei, retained by the Latimeriidae, and their secondary loss in more derived Mawsoniidae.

The number and arrangement of the extrascapular bones is also extremely variable in coelacanths. Three extrascapulars primitively occur in Palaeozoic taxa (e.g., *Miguashaia*, *Diplocercides*, *Allenypterus*), whereas five extrascapulars are known in *Rhabdoderma* and many post-Carboniferous coelacanths (e.g., *Caridosuctor*, *Laugia*, *Coccoderma*). Supernumerary extrascapulars are present in *Diplurus*, *Axelia*, *Macropoma* and *Latimeria*, but are usually an uneven number, whereas in certain mawsoniids the extrascapular series is even, either formed of four (e.g., *Chinlea*, *Parnabaia*) or just two (e.g., *Mawsonia*) extrascapulars, in which the median extrascapular appears to have been medially divided. *Graulia branchiodonta* displays three extrascapulars forming the posterior portion of the skull roof, with two large lateral extrascapulars partially fused to the postparietals and a smaller median extrascapular, detached from the rest of the skull roof. This condition is typical of the mawsoniids *Axelrodichthys*, *Lualabaea*, and *Trachymetopon* but also recalls that of *Guizocoelacanthus* and *Yunnanocoelacanthus*, however in all of these taxa the median extrascapular is considerably larger than in *Graulia branchiodonta*.

#### Cheek (Figs 5, 6, 10, 22, 29; *L*. *chalumnae* Figs 32 and 35)

The cheek of *Graulia branchiodonta* displays the derived conditions of many fossil coelacanths with separated bones, as opposed to Palaeozoic forms with consolidated cheeks like *Miguashaia*, *Diplocercides* and *Spermatodus*, but also the Triassic *Rieppelia* [7]. The occurrence of spiracular bones, wedged between the postorbital, opercular and the skull roof, is variable across coelacanths. Spiraculars are primitively present in the Devonian and Carboniferous genera (e.g., *Diplocercides*, *Serenichthys*, *Rhaboderma*) and still retained in certain Triassic forms (e.g., *Whiteia*, *Sassenia*, *Chinlea*), but they are absent from many Jurassic and Cretaceous mawsoniids (e.g., *Axelrodichthys*, *Mawsonia*, *Parnaibaia*) and Mesozoic latimeriids (e.g., *Foreyia*, *Rieppelia*, *Holophagus*, *Libys*, *Undina*). It is thus difficult to identify clear evolutionary trends, but is appears that within the mawsoniids, spiracular bones were primitively present (e.g., *G*. *branchiodonta*, *Diplurus*, *Indocoelacanthus*) but subsequently lost in the derived mawsoniids, as opposed to the reappearance of the spiracular in derived latimeriids like *Swenzia* and *Latimeria*. The postorbital of *Graulia branchiodonta* is relatively simple, without an anterodorsal excavation (as in latimeriids like *Foreyia*, *Swenzia* and *Latimeria*), and still relatively large (different to the narrow tube found in *Allenypterus*, *Coelacanthus*, *Diplurus*, *Foreyia* or *Ticinepomis*). However, as opposed to more derived mawsoniids in which the postorbital spans the intracranial joint (e.g., *Axelrodichthys*, *Mawsonia*, and *Chinlea*), the postorbital of *Graulia branchiodonta* occupies the primitive position located behind the intracranial joint, as in *Diplurus* and many other fossil coelacanths. Moreover, the path of the infraorbital lateral line canal through the postorbital is located at the anterior portion of the bone in *Graulia branchiodonta*. This is a primitive condition, present in Palaeozoic coelacanths (e.g., *Hadronector*, *Rhabdoderma*) and especially retained in latimeriids (e.g., *Undina*, *Holophagus*, *Swenzia*, *Latimeria*) but unknown to date in mawsoniids (e.g., *Mawsonia*, *Axelrodichthys*, *Diplurus*).

The large squamosal sits in a central position in the cheek, separated from the skull roof by the postorbital and the spiracular, as in many Mesozoic coelacanths (e.g., *Whiteia*, *Holophagus*), with the exception of the reduced and narrow squamosals of *Ticinepomis* and *Diplurus*. *Graulia branchiodonta* displays a preoperculum bearing the jugal lateral line canal through a posteriorly located tube associated with an anterior blade-like projection, a feature exclusively known previously in derived latimeriids (e.g., *Macropoma*, *Lybis*, *Swenzia*, and *Latimeria*), as opposed to stouter and undifferentiated preopercular bones of other fossil coelacanths, revealing a relative plasticity of the shape of the preoperculum in Latimerioidei. The presence or absence of a suboperculum is difficult to survey in fossil coelacanths due to the reduced size of the bone that may have caused it to shift in certain taxa. However, in the Latimerioidei, a suboperculum is primitively present in latimeriids (e.g., *Dobrogeria*, *Foreyia*, *Undina*, *Holophagus*), while its absence is considered a synapomorphy of derived mawsoniids (e.g., *Chinlea*, *Diplurus*, *Axelrodichthys*, and *Mawsonia*). The presence of a suboperculum in an early mawsoniid like *Graulia branchiodonta* points again to a primitive presence in the Latimerioidei and a shared loss of this bone in more derived mawsoniids.

The lachrymojugal is a unique bone of coelacanths with important phylogenetic implications. In *Graulia branchiodonta* it is unfortunately among the worst preserved bones of the cheek but still reveal some interesting features. In *Graulia branchiodonta*, the anterior end of the lachrymojugal is not angled nor expanded as in the Latimerioidei (e.g., *Axelrodichthys*, *Mawsonia*, *Macropoma* or *Latimeria*), thus recalling the simple primitive condition of many fossil coelacanths (e.g., *Miguashaia*, *Diplocercides*, *Sassenia*, and the Laugiidae). Moreover, in *Graulia branchiodonta* the lachrymojugal is separated from the tectal and supraorbital series by the preorbital, which probably carried the posterior canals for the rostral organ, as opposed to other Latimerioidei in which the openings of the rostral organ mark the anterior edge of the lachrymojugal due to the lack of a preorbital.

#### Lower jaw (Figs 5, 6, 9, 22, 29, 30; *L*. *chalumnae* Figs 32, 35)

The coelacanth lower jaw is considered as derived within sarcopterygians and it is also quite variable among coelacanths. The glenoid fossa for the articulation with the quadrate is composed of two separate ossifications in *Graulia branchiodonta*: the articular and retroarticular. This derived condition is known in many post-Devonian coelacanths where it is considered as a paedormorphic trait [2]. The dentary of *Graulia branchiodonta* may have carried its teeth onto small separated (but unpreserved) dental platelets, which together with a hook-shaped shape of the bone is a common feature of derived coelacanths (e.g., *Piveteauia*, *Whiteia*) and of all Latimerioidei, revealing the presence of a muscular lip fold in the living animals as is the case in *Latimeria*. The large principal coronoid is not sutured to the angular in *G*. *branchiodonta*, as in many other fossil coelacanths, but differs from some Jurassic and Cretaceous mawsoniids (e.g., *Lualabaea*, *Axelrodichthys*, *Mawsonia*, and *Trachymetopon*). The dentary also displays a dentary pore located at the suture with the splenial, a common feature of post Carboniferous coelacanths but probably homoplastic as found in the Lower Devonian *Eoactinistia*, one of the oldest coelacanths [15, 50].

The ornamentation of the angular is variable among coelacanths but it can be diagnostic of certain taxa, especially mawsoniids, where the angulars are ornamented by grooves and ridges rather than tubercles (e.g., *Axelrodichthys*, *Mawsonia*, *Trachymetopon*, *Chinlea*) [2, 23, 33, 37]. However, in *Graulia branchiodonta*, the angular is considered smooth, without a clear tubercular, granular, or ridged ornamentation, similar to *Diplurus*, latimeriids and most other non-mawsoniids [2, 40]. The mandibular sensory canal opens through the angular and splenial by numerous pores as in many fossil coelacanths (e.g., *Coelacanthus*, *Laugia*, *Sassenia*, *Whiteia*) but in *G*. *branchiodonta* they are exceptionally large. Moreover, these pores open rather ventrally as in *Axelrodichthys*, *Mawsonia*, *Parnaibaia*, *Diplurus* and *Reidus*, as opposed to non-mawsoniid coelacanths in which the pores open laterally, revealing a new phylogenetic derived feature of mawsoniids.

#### Neurocranium and palate (Figs 5, 6, 8, 11, 21, 22, 29, 30; *L*. *chalumnae* Figs 32, 34, 37)

The braincase of coelacanths shows a clear evolutionary trend towards the reduction of its ossification rate and the fragmentation of a primitively solidly ossified neurocranium into separated smaller ossifications connected by cartilage [2]. *Graulia branchiodonta* displays the derived condition with the separation of the lateral ethmoids, parasphenoid, basisphenoid, prootics, supra- and basioccipital. The development of descending processes in the skull roof on the posterior parietal, postparietal and supratemporal may be associated with this overall reduction of the ossification of the neurocranium [2]. *Graulia branchiodonta* shows descending processes on all of these bones. The descending processes on the anterior ethmosphenoid division of the braincase are carried by the parietals (or posterior parietals) and these appear only in post Devonian coelacanths. In the postparietal portion, descending processes of the postparietals are widespread among the Latimerioidei, but are also present in a few other Mesozoic forms like *Piveteauia*. On the other hand, the descending process of the supratemporal is more common outside the Latimerioidei, as present in earlier forms like *Rhabdoderma*, *Coelacanthus*, *Laugia*, or *Whiteia*. However, its absence in mawsoniids like *Axelrodichthys*, *Mawsonia*, *Diplurus* and *Trachymetopon*, constitutes a diagnostic feature of this clade. Its presence in *Graulia branchiodonta* confirms that the absence of a descending process of the supratemporal is a secondary loss of more derived mawsoniids, probably related to changes in the overall configuration of the skull occurring at the end of the Triassic.

The basisphenoid of mawsoniids characteristically presents a narrow *dorsum sellae*, prominent sphenoid condyles separated by a notch, and a marked inclination between the posterodorsal surface of the basisphenoid and the *processus connectens* [24, 36], whereas in non-mawsoniid coelacanths (e.g., *Latimeria*, *Megalocoelacanthus* or *Dobrogeria*) the sphenoid condyles are less marked and separated by a wider gap [2, 21, 51]. These diagnostic characters usually allow to assign to the Mawsoniidae isolated basisphenoids (e.g., [36]) and are present in the Triassic *Chinlea* and *Diplurus* [37], and in more derived mawsoniids like *Mawsonia* and *Axelrodichthys* [23, 25, 52]. In *Graulia branchiodonta* the basisphenoid shows prominent sphenoid condyles separated by a gap with a relatively narrow *dorsum sellae* supporting its mawsoniid status.

The palate is remarkably complete in *Graulia branchiodonta*, which differs from many other fossil coelacanths in which the smaller elements such as the dermopalatines and ectopterygoid are loosely attached to the autopalatine and palatoquadrate and are thus usually missing. In *Graulia branchiodonta*, the toothed area of the parasphenoid spans almost the entirety of the bone, as in many fossil coelacanths, but different from the reduced area in derived latimeriids (e.g., *Undina*, *Megalocoelacanthus*, *Macropoma* and *Latimeria*). The dermopalatines and ectopterygoid of *Graulia branchiodonta* are similar to the solely denticulated ectopterygoid of *Dobrogeria* [51], as opposed to the combined occurrence of small teeth and larger fangs in *Macropoma* [2] and *Latimeria*. The palatoquadrate of *Graulia branchiodonta* has the typical triangular shape characteristic of coelacanths [2, 46] and is relatively shallow and elongate, congruent with the classic mawsoniid condition as seen in *Axelrodichthys* [2] and *Mawsonia* [53] but different from the stouter palatoquadrates of latimeriids like *Macropoma*, *Megalocoelacanthus*, and *Latimeria*. However, *Graulia branchiodonta* surprisingly displays a ventral swelling of the palatoquadrate, a feature usually associated with the Latimeriidae as present in *Foreyia*, *Libys*, *Macropoma*, *Megalocoelacanthus*, *Undina* and *Latimeria*, but also convergently known outside of the Latimerioidei in *Hadronector* [9] and rarely in derived mawsoniids like *Axelrodichthys* [27]. The occurrence of a moderately developed swelling on the ventral margin of the palatoquadrate in an early mawsoniid like *Graulia branchiodonta* may indicate that this is a common feature primitively present in the Latimerioidei, retained and accentuated by the latimeriids and secondarily lost in most derived mawsoniids.

#### Hyo-branchial skeleton (Figs 5, 6, 12, 22, 29, 30; *L*. *chalumnae* Figs 32 and 37)

Gill arches in coelacanths are rarely preserved, and thus few evolutionary trends can be recognized [2]. The upper portion of the branchial skeleton is poorly known and, in many cases, confusingly illustrated in coelacanths. This is due to the fact that most of our knowledge on the branchial arches come from flattened specimens. Moreover, based on observation on *Latimeria*, a reduction of the ossification rate (decrease of periosteal thickness) occurs from lateral to medial in the cerato, epi- and pharyngobranchial series. This pattern may explain why so few elements from the hyo-branchial skeleton are often missing or unpreserved in fossil coelacanths. *Graulia branchiodonta* exquisitely displays a nearly complete branchial skeleton and three-dimensionally preserved, furnishing new information on the anatomy of the hyo-branchial apparatus in Mesozoic coelacanths.

A consolidation of the basibranchial dentition is one of the single evolutionary trends identified by Forey [2], from numerous small plates in primitive forms (e.g., *Laugia*, *Whiteia*) to fewer and larger plates in more derived coelacanths (e.g., *Macropoma*). However, tooth plates are rarely found articulated in fossil coelacanths, and thus clear evolutionary patterns cannot be identified. The basibranchial tooth plates of *Graulia branchiodonta* consist of four plates organized in two pairs, with the anterior pair being smaller than the posterior one. This condition is similar to that of mawsoniids like *Diplurus* and *Axelrodichthys*, but with a different size ratio. In *Axelrodichthys*, the largest pair is anterior while the posterior one is smaller and narrower. Moreover, in *Diplurus*, an extra pair of plates occur laterally. However, the arrangement of the basibranchial plates in *Graulia branchiodonta* also resembles *Latimeria*, in which the anterior pair is smaller than the posterior one, but in *Latimeria* a median plate is wedged in the midline.

The ceratobranchials of *Graulia branchiodonta* bear several rows of tooth plates, carrying relatively large teeth, similar to the condition of *Rhabdoderma* and *Latimeria*, analogous in function to the gill-rackers of actinopterygians [42, 48]. Due to the reduction in the ossification rate of the epibranchial series, only the second and third epibranchials are known in *Graulia branchiodonta*. Few other fossil coelacanths preserve the dorsal elements of the branchial skeleton: in *Laugia*, only the first two epibranchials and first pharyngobranchial are known, whereas in *Dobrogeria* two putative epibranchials have been found, and in *Rhabdoderma* three epibranchials have been identified [2, 51, 54]. The interhyal is rarely found in fossil coelacanths, as it was usually partly ossified (e.g., *Diplocercides*). In *Graulia branchiodonta* it is exceptionally preserved as a small tubular element, resembling that of *Latimeria*. The urohyal has the typical coelacanth shape but in *Graulia branchiodonta* it is more robust anteriorly, it has a higher width/length ratio and the posterior bifid projections are more developed and pointed than in *Latimeria*.

#### Pectoral and pelvic girdles and fins (Figs 5, 6, 16, 23, 27, 29, 30; *L*. *chalumnae* Figs 32 and 38)

The pectoral girdle of coelacanths is deemed as remarkably conservative in shape and structure [2]. However, *Graulia branchiodonta* reveals several unexpected features uncommon in many known fossil coelacanths. The anocleithrum of *Graulia branchiodonta* presents an intraspecific variation as it asymmetrically displays both the simple and the distally forked shape (see below). The latter condition is uncommon in fossil coelacanths, being only present in the Jurassic *Coccoderma* and *Macropoma*. No known mawsoniid shows the bifid condition and it appears that it may be more common in latimeriids as asymmetrical anocleithri are also known in *Latimeria* [41, 55], thus highlighting the relative plasticity of this feature. The interclavicle is absent in *Latimeria* and is rarely found in fossil specimens, and thus was previously assumed to be lacking in coelacanths [56]. However, interclavicles are known in the Triassic *Laugia*, *Whiteia*, *Foreyia* (even if there are some doubts about its identification) and now in *Graulia branchiodonta*. In all these forms, excluding *Foreyia*, the interclavicle is not ornamented and lies subdermally in the ventral midline of the pectoral girdle, partly overlapped by the clavicles, thus supporting its endochondral nature in coelacanths, as opposed to the dermal origin of the interclavicle in other sarcopterygians and actinopterygians [2, 57]. The scapulocoracoid is a rather small ossification in *Graulia branchiodonta*, as in other fossil coelacanths (e.g., *Macropoma*, *Axelrodichthys*), different from the extensive but mostly cartilaginous scapulocoracoid of *Latimeria* [55]. As such, the large ventral blade of the scapulocoracoid cannot be retrieved in fossil specimens, but its presence is revealed by a long groove on the internal surface of the cleithrum, as observed in *Latimeria*. As in many other fossil coelacanths, the endoskeletal mesomeres and radials forming the pectoral and pelvic fins are unossified, with the exception of *Shoshonia* [58] and *Laugia* [2, 54], but the relative length and width of the pectoral and paired fins can be extrapolated from the gap between the proximal end of the lepidotrichia and the girdle in case of exceptional preservation as in *Graulia branchiodonta*.

#### Median fins (Figs 16, 26, 29)

The base of the first dorsal fin of *Graulia branchiodonta* consists of a roughly triangular basal plate-like element, displaying two ridges anteriorly converging at the ossification center of the plate, similar to the condition of *Undina*, *Macropoma* and *Latimeria* [2] but different from the stouter and less carved bases of *Coelacanthus*, *Diplurus*, or *Laugia*. The ventral edge is smooth as in many Mesozoic coelacanths and differs from the emarginated condition of certain Palaeozoic coelacanths (e.g., *Lochmocercus*, *Caridosuctor*, *Rhabdoderma*) [59]. These indentations received the tips of the adjacent neural arches, whereas in *Graulia branchiodonta*, the neural spines underlying the base of the first dorsal fin are relatively shorter than the rest, creating a shallow gap for the accommodation of the plate, similar to the condition in *Coelacanthus*, *Laugia*, *Luopingcoelacanthus* and *Diplurus* [2, 17]. Only the anteriormost portion of the base of the second dorsal fin is ossified in *Graulia branchiodonta* and it displays two antero-ventral processes as in many other coelacanths (e.g., *Coelacanthus*, *Laugia*, *Diplurus*, *Macropoma*, *Axelrodichthys*, *Holophagus*, *Latimeria*) but in *Graulia branchiodonta* the anterodorsal projection is slightly larger than the ventral one as in the latimeriids *Macropoma* and *Holophagus*, but different from the more elongated ventral projection of *Axelrodichthys* or *Rhabdoderma*. The base of the anal fin is simple, formed by a single anterior projection as in the great majority of fossil coelacanths, with the exception of *Diplurus* and *Luopingcoelacanthus* where it displays two projections anteriorly, matching those of the second dorsal fin [2, 17].

The lepidotrichia of the first dorsal fin are ornamented in *Graulia branchiodonta* by small denticles (probably odontodes) located on the leading edge of the fin and postero-dorsally oriented. Similar denticles are common in coelacanths from the Mesozoic to Recent (e.g., *Axelrodichthys*, *Mawsonia*, *Trachymetopon*, *Foreyia*, *Rieppelia*, *Undina* and *Latimeria*) but appear to be absent in the Palaeozoic (e.g., *Miguashaia*, *Allenypterus*, *Rhaboderma*). Denticles in the fin rays are also known in *Alcoveria* (described as “fulcra” by Beltan [60]). Interlocking flanges are only primitively present in Devonian coelacanths like *Gavinia* [14], *Miguashaia* [13], *Diplocercides* [61] and *Shoshonia* [58], but disappear in more derived forms.

The lobation of the second dorsal and anal fins has increased across coelacanth history, but this is difficult to evaluate given the rare preservation of the endoskeleton of the paired, second dorsal and anal fins [2]. The disposition of the fin rays may give a glimpse of the internal arrangement of the fin skeleton and reveal that many Mesozoic taxa already displayed the general configuration of the extant *Latimeria*. This, associated with the stiffening of the vertebral column by the lack of centra and the development of a broad notochordal shaft, indicates that, as in *Latimeria*, the thrust for locomotion in *Graulia branchiodonta*, as well as in many other fossil coelacanths, was created mainly by the caudal fin and the sculling of the second dorsal and anal fins. The main source of propulsion in “classically-shaped” coelacanths was thus not the flexion of the body but rather the combined movement of the caudal and second dorsal and anal fins. Interesting departures from this conservative morphology include the Paleozoic *Allenypterus*, *Holoperygius* and to some degree the Mesozoic *Foreyia*.

#### Caudal fin (Figs 15, 25, 29)

The diphycercal caudal fin of coelacanths is undoubtedly the chief and most recognizable attribute of the group, characterizing “anatomically modern” coelacanths as opposed to “anatomically primitive” taxa like *Miguashaia* and *Gavinia* with classical sarcopterygian heterocercal tails [13–15]. However, the general shape of the caudal fin and the number of the elements constituting it is quite variable among coelacanths. The supplementary caudal lobe of *Graulia branchiodonta* is poorly preserved, as in many other fossil coelacanths due to a lack of ossification of the radials, but a gap between the dorsal and ventral lobes in MHNG GEPI V5788 matches the overall shape of the supplementary lobe and reveal that it may have remained within the posterior boundaries of the tail, similar to the condition of *Whiteia*, *Axelrodichthys*, *Macropomoides* or *Latimeria*, as opposed to longer lobes in *Rhabdoderma*, *Diplurus*, *Laugia*, *Belemnocerca* [2, 5].

The fin rays also reveal several evolutionary trends within coelacanth history. Early coelacanths have a more than one-to-one relationship between fin rays and radials in the caudal fin (e.g., *Miguashaia*, *Serenichthys*, *Diplocercides*, *Holopterygius*, *Allenypterus*, *Lochmocercus*) [8, 9, 13, 62, 63] and some show distal bifurcation of the lepidotrichia (e.g., *Miguashaia*, *Gavinia*, *Diplocercides*) [13, 14, 61]. All Mesozoic coelacanths display the derived condition of unbranched fin rays with a one-to-one relationship between the radials and lepidotrichia (the previously described condition of *Rieppelia* [7] may be incorrect as pairs of hemirays may have been identified as separated lepidotrichia). In.*Graulia branchiodonta*, approximately 15 radials and their connected lepidotrichia have been confidently identified in the dorsal lobe of the caudal fin, a relatively low number, similar to the condition of *Rhabdoderma*, *Whiteia*, *Ticinepomis*, *Foreyia*, *Rieppelia*, or *Diplurus*, and only slightly fewer than in other mawsoniids like *Axelrodichthys* [2, 7]. The caudal fin rays are richly segmented in their distal portion, a common feature of all coelacanths, with the exception of the Triassic *Rebellatrix*, which has unusually large unsegmented fin rays in the caudal fin, associated with its presumed fast swimming abilities [5]. The first two or three rays of the dorsal lobe in *Graulia branchiodonta* carry small denticles, identical to those present on the first dorsal fin, on the segmented as well as on the proximal unsegmented portion of the lepidotrichia. Denticles on the leading edge of the first rays of the caudal lobes are also known to be present in *Whiteia*, *Changxingia*, *Luopingcoelacanthus*, *Undina*, *Holophagus*, *Macropoma*, *Macropomoides*, *Ticinepomis*, *Foreyia*, and *Rieppelia* (in the latter all fin rays are ornamented with denticles) and in the mawsoniids *Diplurus*, *Heptanema*, *Lualabaea*, *Axelrodichthys* [2].

#### Scales (Figs 16 and 28)

The scales of *Graulia branchiodonta* are ovoidal to slightly quadrangular in shape with a slightly antero-posterior elongation and a rounded anterior margin. This shape is reminiscent of *Guizhoucoelacanthus* and *Latimeria*, as opposed to more ovate or rounded scales in taxa like *Wimania*, *Diplurus*, or *Axelrodichthys* [64]. The scales are also ornamented by small, elongated tubercles, similar to that of many coelacanths (e.g., *Wimania*, *Holophagus*, *Macropoma* or *Latimeria*). However, they do not present any ridges, as usually found in mawsoniids (e.g., *Axelrodichthys*, *Chinlea*, *Diplurus*, *Heptanema*, *Lualabea*, *Mawsonia*). Moreover, in these taxa, the size of the ridges is differentiated with large ridges usually located in the central portion of the exposed area of the scales associated with smaller ridges laterally [2, 27, 29, 64, 65], probably constituting a diagnostic feature of derived mawsoniids, as opposed to the simply tuberculated primitive condition of *G*. *branchiodonta*. The partially preserved scale posterior to the skull appears to be devoid of ornamentation, suggesting a possible variation of the ornamentation across the body, as known in other fossil coelacanths (e.g., *Miguashaia*, *Macropoma*, *Luopingcoelacanthus*) [2, 13, 17, 64].

#### Ossified lung and ossified ribs (Figs 14, 15, 18, 19, 24, 25, 29)

Ossified lung plates are known in numerous fossil coelacanths, both from the Palaeozoic (e.g. *Coelacanthus madagascarensis*, *Holophagus gulo*, *Polyosteorhynchus simplex*, *Rhabdoderma elegans*, *Caridosuctor popolosum*, *Hadronector donbairdi* [2, 66–68], and the Mesozoic (e.g., *Piveteauia madagascarensis Mawsonia gigas*, *Macropoma lewesiensis*, *Coccoderma suevicum*, *Libys polypterus*, *Undina (U*. *penicillata* and *U*. *cirinensis)*, *Axelrodichthys araripensis*, *Laugia groenlandica*, *Swenzia latimerae*, *Luopingcoelacanthus eurylacrimalis*, *Trachymetopon liassicum*) [17, 24, 43, 49, 52, 54, 69–75]. The preservation of these plates confirms the presence of functional lungs in fossil coelacanths, which acted as regulators of volume variation and protected against hydrostatic pressure [44]. Other possible functions include sound production as a resonating chamber or some relation to hearing [2]. Certain forms like *Piveteauia* and *Axelrodichthys* have a divided ossified lung formed by two chambers separated by a constriction, while in *Swenzia*, *Coccoderma* and *Macropoma* the lung is undivided. *Graulia branchiodonta* is thus unique among fossil coelacanths in displaying a three-chambered ossified lung, with the first chamber bearing paired ossified crests that may have met the notochord laterally. *Macropoma mantelli* [72] and *Undina cirinensis* [71] present a bottle neck anteriorly, but this has not been identified in *Graulia branchiodonta*.

Pleural ribs are known to protect the lungs and viscera as well as assisting in air-breathing in piscine sarcopterygians (e.g., dipnoans and coelacanths). However, ribs are not always associated with calcified lungs in coelacanths. In many fossil coelacanths with calcified lungs the ribs are reduced or absent (e.g., *Hadronector*, *Rhabdoderma*, *Coelacanthus*, *Piveteauia*, *Holophagus*, *Undina*, *Swenzia*), whereas certain coelacanths lacking a calcified lung display well-developed ribs (e.g., *Diplurus*). Abdominal ribs constitute the main synapomorphy of Mawsoniidae, since they have been considered to be exclusively present in all known Mesozoic members of the family (i.e, *Axelrodichthys*, *Chinlea*, *Diplurus*, *Heptanema*, *Indocoelacanthus*, *Mawsonia*, *Parnaibaia*, *Trachymetopon* and *Graulia branchiodonta*). However, accounts of ossified ribs are known outside of the mawsoniids, as they have been described to be present in the Permian *Changxingia* [76], which is unfortunately flattened and too incompletely preserved to be reliably included in a phylogenetic analysis, but also in the Triassic *Laugia* [2, 54], the Jurassic latimeriid *Holophagus* [2] and the Cretaceous *Macropomoides* [2], thus questioning the exclusivity of this character to the Mawsoniidae (although arguably the ribs are longer in mawsoniids than in any other taxa). In *Graulia branchiodonta*, the ribs show a strange size gradient, becoming longer from anterior to posterior, whereas in many taxa (e.g., *Laugia*, *Holophagus*, *Macropomoides*) the gradient is reversed, with the ribs becoming shorter from anterior to posterior. *Diplurus* shows a wavy profile with the longest ribs being located in the mid-section of the abdomen and shortening towards the rear. However, no gap in size between the posterior most ribs and the first haemal spines occurs in *Graulia branchiodonta*, as opposed to *Diplurus*, but rather a progressive transition from the ribs into the haemal arches and spines as in *Laugia*, *Macropoma*, and *Axelrodichthys*.

### Ontogenetic stages

The two specimens of *Graulia branchiodonta* (MHNG GEPI V5787, holotype, and MHNG GEPI V5788) are almost complete and have similar body sizes, both measuring an estimated 3,5 cm from the tip of the snout to the posterior end of the pectoral girdle, with an estimated TL of 16 cm. TL was estimated by carefully reconstructing specimen MHNG GEPI V5788, for which we retrieved the skull and the complete axial skeleton, excluding the posterior tip of caudal peduncle. In *Latimeria chalumnae*, late embryos measure 35 cm TL and small juveniles measure around 40 cm TL, while specimens measuring 132 cm TL are already considered adults (230% longer than the juveniles) [77] with the largest specimens measuring up to around 2 m TL [78]. In *Axelrodichthys araripensis* the size difference between juvenile and adults is more dramatic, with the smallest juvenile measuring 70 mm TL and adults estimated up to 1–2 m TL (roughly 1300% up to 2700% more than the juveniles) [79]. We may regard the specimens (MHNG GEPI V5787, holotype, and MHNG GEPI V5788) as juveniles given some marked juvenile features like the large mandibular sensory canal and pores (Fig 9). It was established by Hensel [80] and Forey [2] that the mandibular sensory canal is relatively large in embryos of *L*. *chalumnae* and it shrinks down progressively to a narrow tube in adults. The huge size of the mandibular canal and pores of the specimens may suggest a different lifestyle of the juveniles that would rely more on such part of the sensory system compared to adults. Furthermore, the ornamentation of the scales consisting in rounded tubercles (Figs 17 and 28) may support the juvenile status of the two specimens. In *A*. *araripensis*, juveniles share a similar ornamentation pattern of rounded tubercles on the scales, which changes in the adults having instead irregularly spaced horizontal ridges on the scales [79].

Finally, a few examples of intraspecific variations are known in *Graulia branchiodonta*. The number of premaxillary teeth varies between the two specimens: from three in the holotype MHNG GEPI V5787, to four in MHNG GEPI V5788. Such variations are also known in *Caridosuctor* and *Whiteia* [2]. In the pectoral girdles of the *Graulia branchiodonta*, the anocleithra can be represented by symmetrical blade-like elements with a pointed dorsal section (MHNG GEPI V5787) (Fig 13) or being dorsally bifurcated on one side and pointed on the other (in MHNG GEPI V5788) (Fig 23). These asymmetrical variations also occur in *Latimeria* [41, 55].

### Conclusion

Following the discovery of the extant coelacanth *Latimeria* in 1938, which was "undoubtedly the most important zoological discovery of the 20th century" [81], its anatomy became one of the best studied and best known among vertebrates. On the other hand, knowledge of extinct coelacanths has been limited by the preservation of fossils with material generally represented by articulated but flattened specimens, making volumetric reconstruction difficult, or by material preserved in 3D but often fragmentary. The incredible quality of conservation of two complete and 3D preserved specimens from the Middle Triassic Muschelkalk of Lorraine, Eastern France, combined with the latest X-ray technologies, has allowed a complete description of an extinct coelacanth with an unprecedented level of detail, comparable to the work carried out on the skeleton of extant species. Most skeletal elements were deeply embedded in the rock matrix and perfectly preserved, only the external surface of some bones had been slightly damaged during mechanical preparation. The two specimens are referred to a juvenile of *Graulia branchiodonta* gen. et sp. nov., the best-preserved Mesozoic coelacanth to date, reconstructed here as the oldest Mawsoniidae that roamed in European marine environments during the Triassic (247–237 Mya).

Several characters are diagnostic of *Graulia branchiodonta*, such as the presence of anteromedial processes on the posterior parietals for a tighter suture with the anterior parietal, one pair of large extrascapulars with a much smaller median element, and large tabulars (supratemporals), likely fused with another pair of lateral extrascapulars for a stronger protection of the enlarged sensory canals. Notable ontogenetic variation occurs within this new species. Among these, the size of the cavities associated with the course the sensory canals across the skull is remarkable, even for a juvenile fish, suggesting a strong sensitivity to minimal water movements. Such features, combined with the streamlined shape of the skull and relatively large fins may suggest a very active predatory lifestyle for *Graulia branchiodonta*. The presence of large series of teeth on the ceratobranchials and the absence of fangs in the jaws (simply covered with many tiny teeth) may also indicate a predilection for suction-feeding [24] of strong, dynamic prey that would be hold tightly in the oral cavity by the heavily denticulated ceratobranchials to facilitate a rapid swallowing. Finally, we identified an ossified lung displaying three separated chambers, a unique condition for an extinct coelacanth. Further studies are currently underway to better understand its function and a possible relationship to underwater hearing.

Initial phylogenetic analysis showed some phylogenetic uncertainty related to the placement of *Graulia branchiodonta* among Triassic coelacanth taxa, despite the great amount of morphological information. Phylogenetic uncertainty and incongruences did not come from lack of data (given the completeness of the specimens), but may have derived from conflicting distribution of character states, as the new taxon shows an interesting combination of mawsoniid and latimeriid features. In particular, several characters previously known only in latimeriids are now recognized in mawsoniids, with *Graulia branchiodonta* possibly illustrating the primitive condition for the Latimerioidei. Such characters include the presence of anterior projections of the supratemporal commissure, a reduced number of premaxillary teeth (four or less), the presence of an anterior blade-like projection of the preoperculum and the presence of the suboperculum. Another potential symplesiomorphy is related to the ornamentation of the scales that are tuberculated in *Graulia branchiodonta* as opposed to being covered with irregular ridges in most mawsoniids. However, because the specimens of *Graulia branchiodonta* are likely juveniles we suspect that the ornamentation of the scales may change across the ontogeny. We may discover new adult specimens of *Graulia branchiodonta* with a more “typical” mawsoniid-like ornamentation of the dermoskeleton (i.e. skull roof bones and scales with ridges).

Despite a now well-established diversity peak (both taxonomical and morphological) during the Triassic, the effects of the Permo-Triassic biodiversity crisis among coelacanths are still difficult to quantify. We suspect that the coelacanth radiation that took place at the beginning of the Triassic, within a relatively short time following the mass-extinction, makes it currently challenging to recognize shared characters in phylogenetic analyses and to properly resolve interrelationships between closely related, and sometimes contemporary, taxa. New Triassic forms like *Graulia branchiodonta* illustrate our still approximative understanding of the patterns of recovery of coelacanths after the “Great dying” and encourage a closer look at fossil specimens thanks to the use of new X-ray microtomography techniques,to better understand one of the most important episodes in the long evolutionary history of these iconic fishes.

## Materials and methods

### Specimens

MHNG GEPI V5787 and MHNG GEPI V5788 (Fig 1) comprising a completely preserved body, including the complete skull, axial skeleton and relatively complete paired and median fins. The specimens were discovered by Jean-Philippe and François-Xavier Blouet in spring 2012 during the excavation work before the building of a new TGV train line, near the village of Sarraltroff, 57400 Moselle, France (GPS coordinates and elevation available upon request).

### Micro computed tomography scanning, segmentation, rendering

We used Synchrotron radiation propagation phase contrast micro-computed tomography to study the specimens MHNG GEPI V5787 and MHNG GEPI V5788. Both specimens were scanned at the European Synchrotron and Radiation Facility, Grenoble, France on beamline BM05 (DOI: 10.15151/ESRF-ES-788657609). The beamline setup was for a filtered white beam with a 2000nm thick luAg scintillator, a sample-detector distance of 3.5 meters, and an isotropic voxel size of 14.9μm. MHNG GEPI V5788 was imaged with a filtered white beam setup of 117.5 keV, with molybdenum (2.16mm) and aluminium (4.47 mm) attenuator filters. MHNG GEPI V5787 was imaged with a filtered white beam setup of 110 keV, with copper (6.41mm) attenuator filters. Acquisition was done with a PCO edge 4,2 sCMOS (PCO, Kelheim Germany), recording 6000 projections in accumulation mode, with a total exposure time of 120ms, with four accumulated images of 30ms for MHNG GEPI V5788, and a total exposure time of 27ms, with three accumulated images of 9ms for MHNG GEPI V5787 [82]. The specimens were scanned with a half-acquisition setup [83], with an offset of 900 pixels. The final tomographic volume was reconstructed using PyHST2 [84] and the single distance Paganin phase retrieval approach [85]. The original datasets of the scans are available for free download from the ESRF Paleontology Database (https://paleo.esrf.eu/).

Synchrotron microCT datasets were segmented manually in Dragonfly 4.1 (ORS). Meshes were exported as.ply files and imported in Blender V.3.1.2 (Blender Institute). In Blender, the skeletal elements of the skull of MHNG GEPI V5787 were rearranged in anatomical position using as guidance a 3D model of the skull of *L*. *chalumnae* [86]. To interpret the elements of the postcranial skeleton of MHNG GEPI V5787 and MHNG GEPI V5788, a CT scan of the whole body of *L*. *chalumnae* MHNG 1080.070 (unpublished data) was performed at the Unit of Forensic Imaging and Anthropology, University Center of Legal Medicine, Geneva-Lausanne, and manually segmented. As the neurocranium of *L*. *chalumnae* MHNG 1080.070 was partly damaged, a CTscan of the whole body of a second specimen of *L*. *chalumnae* was downloaded from MorphoSource.org (Media 000398327) to segment the neurocranium (unpublished data). Finally, the 3D models of the fossil specimens were rendered in Blender with two different methods. To produce images with colorized bones, the render engine was set to Workbench, each skeletal elements was vertex colorized, convexities and concavities were enhanced and the contours were highlighted with a thin black line. To produce images with a photorealistic bone-like texture, the render engine was set to Cycles to design a complex shading pattern with several nodes including Noise Texture with a ColorRamp from brown to white and a slight Bump factor. Ambient occlusion was applied to some renders to highlight the cavities. The same lighting setup was employed for all renderings in Blender, consisting in a strong sun-like light coming from the upper left corner of the image, and a softer point light coming from below to soften the shadows. The segmented 3D volumes of *L*. *chalumnae* were instead rendered in Amira 9 (ThermoFisher), where a color map from brown to white was applied to the gradient of density. As a result, the denser parts of the skeleton are colored white and the softer parts are brown. Images were exported from Blender and Amira as.tiff files and imported in Adobe Photoshop 2021 to improve the exposure and combine different images to produce the final plates.

### Phylogenetic analysis

Our data matrix (S1 Table) was assembled in Microsoft Office Excel and NotePad++ and it is based on the recently published data matrix of coelacanths from Ferrante and Cavin [7]. Our matrix includes 48 taxa and 111 characters, of which 90 related to the cranial skeleton and 21 to the postcranial (S1 Appendix). Character 66 (subopercular branch of the mandibular sensory canal {0 absent / 1 present}) was removed for all taxa as we noticed some confusion on the way such character was originally defined. It corresponds to character 60 in Forey 1998, where the presence or absence of the subopercular branch of the mandibular sensory canal is inferred by the presence of a putative exit pore for such canal on the angular, pore that Forey claimed to be present in *L*. *chalumnae*. However, from the 3D skull of *L*. *chalumnae* in Manuelli et al. [86], from Hensel and Balon [87], and from this study (Fig 35F) it is clear that such exit pore does not exist in *L*. *chalumnae*. Instead, the subopercular branch of the mandibular sensory canal shares the same exit pore as the preopercular branch and the branching happens outside the lower jaw, not inside.

We carried out our parsimony analyses in PAUP4 [88]. We performed heuristic searches with random stepwise addition sequence, 1000 replicates, holding 10 trees at each step. We selected the tree-bisection-reconnection branch-swapping algorithm (TBR), swapping only on the best trees. For statistical support, we calculated Bootstrap values and Bremer values. Bootstrapping randomly select columns from the matrix until it matches the original matrix’s number of columns. Since it revisits the original matrix when choosing a new column, some characters may be duplicated in the bootstrap matrix, while others may be excluded. This process is commonly referred to as resampling the data with replacement. In practical terms, although taxa can be randomized, bootstrapping typically involves randomizing characters. Support values for each node are computed through bootstrapping, reflecting the fraction of samples showing that specific node. The highest support value is 100, and values below 70 are generally regarded as weak. Bremer support, also called branch support, is the disparity in the number of steps between the score of the Most Parsimonious Tree(s) and the score of the most parsimonious tree that lacks a specific clade (node, branch). Usually, a Bremer score of 3 or more is considered good [89]. Bootstrap values were calculated as follows: 500 replicates, including groups compatible with 50% majority-rule consensus. PAUP does not calculate Bremer supports directly, so manipulation of the dataset was required to obtain such values indirectly.

### Nomenclatural acts

The electronic edition of this article conforms to the requirements of the amended International Code of Zoological Nomenclature, and hence the new names contained herein are available under that Code from the electronic edition of this article. This published work and the nomenclatural acts it contains have been registered in ZooBank, the online registration system for the ICZN. The ZooBank LSIDs (Life Science Identifiers) can be resolved and the associated information viewed through any standard web browser by appending the LSID to the prefix ""http://zoobank.org/"". The LSID for this publication is: urn:lsid:zoobank.org:pub:03918C29-09F8-4BEE-8808-2F629B779B1A. The electronic edition of this work was published in a journal with an ISSN, and has been archived and is available from the following digital repositories: PubMed Central, LOCKSS.

## Supporting information

S1 Table Data matrix of characters used for the series of phylogenetic analyses. Based on the matrix of Ferrante and Cavin [7].
(PDF)

S1 Appendix List of characters employed in the phylogenetic analyses. The character numbers correspond to the ones of Ferrante and Cavin [7] unless stated otherwise.
(DOCX)

## Abbreviations

Institutional abbreviations MHNG: Muséum d’Histoire naturelle de Genève
ESRF: European Synchrotron Radiation Facility
Anatomical abbreviations a.Az: anterior anazygal
a.b: anal fin basal plate
a.Cz: anterior catazygal
a.Dpl: anterior dermopalatine
a.h: artificial hole
a.r: anal fin ray
a.ros: anterior pore of rostral organ
a.stt.comm: anterior branches of supratemporal commissure
Acl: anocleithrum
Ang: angular
ant.pr: antotic process
Art: articular
Aut: autopalatine
Bb: basibranchial
Boc: basioccipital
Bsph: basisphenoid
Cb: ceratobranchial
Ch: ceratohyal
Cl: cleithrum
Cla: clavicle
Co: coronoid
d.p: dentary pore
d.p.p.Pa: descending process of the posterior parietal
d.p.Pp: descending process of postparietal
d.p.Stt: descending process of supratemporal
d.pl.Bb: basibranchial dental plate
d1.b: first dorsal fin basal plate
d2.b: second dorsal fin basal plate
De: dentary
Dpl: dermopalatine
Eb: epibranchial
Ecl: extracleithrum
Ecpt: ectopterygoid
Enpt: entopterygoid
Exo: exoccipital
Ext: extratemporal
f.pop.s.c & sop.br: foramen for the preopercular sensory canal and subopercular branch
f.s.opth: foramen for superficial ophthalmic nerve
Gu: gular plate
gu.p.l: gular pit line
h.a+h.s: haemal arch plus haemal spine
hyo.f: hyoidean fold
i.j: intracranial joint
Ib: infrapharyngobranchial
Icla: interclavicle
Ih: interhyal
III: oculomotor foramen
io.s.c: infraorbital lateral line sensory canal
j.s.c: jugal sensory canal
ju.ca: jugular canal
L.Eth: lateral ethmoid
L.Ex: lateral extrascapular
l.l.c: lateral line canal
L.Ro: lateral rostral
Lep: lepidotrichia
Lj: lachrymojugal
M.Ex: median extrascapular
m.p.l: middle pit line
M.Ro: median rostral
Ma: maxilla
mc: mandibular sensory canal
Mm: mentomeckelian
Mpt: metapterygoid
n.a: neural arch
n.s: neural spine
Na: nasal
o.p.l: oral pit line
Od: odontode
Op: operculum
ot.ca: otic capsule
ot.s.c.m: medial branch of the otic sensory canal
otc: otic sensory canal
p.Az: posterior anazygal
p.b: pelvic fin basal plate
p.Cz: posterior catazygal
p.Dpl: posterior dermopalatine
p.l.l: pore lateral line
p.p.l: posterior pit line
p.Pa: posterior parietal
p.ros: posterior pore of rostral organ
p.w.Pro: posterior wing of prootic
Pa: anterior parietal
Pb: pharingobranchial
pit.f: pituitary fossa
pl.Gu: gular pit line
pl.Pp: postparietal pit line
plr: pleural rib
Pmx: premaxilla
po: pores
Po: postorbital
Pop: preoperculum
pop.sc: preopercular sensory canal
Pp: postparietal
Pr: postrostral
pr.Co: principal coronoid
pr.con: processus connettens
pr.em: prefacial eminence
Pra: prearticular
Pro: preorbital
Prot: prootic
psmax: pseudomaxillary fold
Psph: parasphenoid
pv.b: pelvic fin basal plate
Q: quadrate
ra: radial
Rart: retroarticular
Ro: rostral
sac.c: saccular chamber
Sb: suprabranchial
Scc: scapulocoracoid
Scl: supracleithrum
So: supraorbital
Soc: supraocciptal
soc: supraorbital lateral line sensory canal
Sop: suboperculum
Sop.br: subopercular branch
sop.fl: subopercular flap
Sp: spiracular
sph.co: sphenoid condyle
Spl: splenial
spt.fos: suprapterigoyd fossa
Sq: squamosal
Stt: supratemporal
Stt.comm: supratemporal commissural lateral line canal
Sy: symplectic
t.p.Bb: tooth plates of basibranchial
t.p.De: dentary tooth plates
t.Pmx: premaxillary teeth
Te: tectal
Uhy: urohyal
V1: profundus foramen
Ve: vertebrae
VI: abducens foramen
Vo: vomer

## References

[1] Cloutier R., Patterns trends, and rates of evolution within the Actinistia. The biology of Latimeria chalumnae and evolution of coelacanths. 1991. pp. 23–58.

[2] Forey P. History of the coelacanth fishes. Springer Science & Business Media; 1997.

[3] Schultze HP. Mesozoic sarcopterygians. Mesozoic Fishes 3—Systematics, Paleoenvironments and Biodiversity. Miinchen, Germany: Verlag Dr. Fl’iedrich Pfeil; 2004.

[4] Toriño P, Soto M, Perea D. A comprehensive phylogenetic analysis of coelacanth fishes (Sarcopterygii, Actinistia) with comments on the composition of the Mawsoniidae and Latimeriidae: Evaluating old and new methodological challenges and constraints. Historical Biology. 2021;33: 3423–3443.

[5] Wendruff AJ, Wilson MV. A fork-tailed coelacanth, Rebellatrix divaricerca, gen. et sp. nov.(Actinistia, Rebellatricidae, fam. nov.), from the Lower Triassic of Western Canada. Journal of Vertebrate Paleontology. 2012;32: 499–511.

[6] Cavin L, Mennecart B, Obrist C, Costeur L, Furrer H. Heterochronic evolution explains novel body shape in a Triassic coelacanth from Switzerland. Scientific Reports. 2017;7: 13695. doi: 10.1038/s41598-017-13796-0 29057913 PMC5651877

[7] Ferrante C, Cavin L. Early Mesozoic burst of morphological disparity in the slow-evolving coelacanth fish lineage. Scientific Reports. 2023;13: 11356. doi: 10.1038/s41598-023-37849-9 37443368 PMC10345187

[8] Friedman M, Coates MI. A newly recognized fossil coelacanth highlights the early morphological diversification of the clade. Proceedings of the Royal Society B: Biological Sciences. 2006;273: 245–250. doi: 10.1098/rspb.2005.3316 16555794 PMC1560029

[9] Lund R, Lund WL. Coelacanths from the Bear Gulch Limestone (Namurian) of Montana and the evolution of the Coelacanthiformes. Carnegie Museum of Natural History; 1985.

[10] Smith JLB. A living coelacanthid fish from South Africa. Transactions of the Royal Society of South Africa. 1940;28: 1–106.

[11] Pouyaud L, Wirjoatmodjo S, Rachmatika I, Tjakrawidjaja A, Hadiaty R, Hadie W. A new species of coelacanth. Genetic and morphologic proof. Comptes Rendus de L’academie des sciences Serie III, Sciences de la vie. 1999;322: 261–267. 10.1016/s0764-4469(99)80061-4 10216801

[12] Kadarusman S, H.Y. P, L. H, R. H, I.B. G, E. W, et al. A thirteen-million-year divergence between two lineages of Indonesian coelacanths. Scientific reports. 2020;10: 192. doi: 10.1038/s41598-019-57042-1 31932637 PMC6957673

[13] Cloutier R. The primitive actinistian Miguashaia bureaui Schultze (Sarcopterygii). Devonian fishes and plants of Miguasha. Quebec, Canada; 1996. pp. 227–247.

[14] Long JA. A new genus of fossil coelacanth (Osteichthyes: Coelacanthiformes) from the Middle Devonian of southeastern Australia. Records of the Western Australian Museum. 1999;Supplement, 57: 37–53.

[15] Zhu M, Yu X, Lu J, Qiao T, Zhao W, Jia L. Earliest known coelacanth skull extends the range of anatomically modern coelacanths to the Early Devonian. Nature Communications. 2012;3: 772. doi: 10.1038/ncomms1764 22491320

[16] Cavin L, Furrer H, Obrist C. New coelacanth material from the Middle Triassic of eastern Switzerland, and comments on the taxic diversity of actinistans. Swiss Journal of Geosciences. 2013;106: 161–177.

[17] Wen W, Zhang QY, Hu SX, Benton MJ, Zhou CY, Tao X, et al. Coelacanths from the Middle Triassic Luoping Biota, Yunnan, South China, with the earliest evidence of ovoviviparity. Acta Palaeontologica Polonica. 2013;58: 175–193.

[18] Romano C, Koot MB, Kogan I, Brayard A, Minikh AV, Brinkmann W, et al. Permian–Triassic Osteichthyes (bony fishes): diversity dynamics and body size evolution. Biological Reviews. 2016;91: 106–147. doi: 10.1111/brv.12161 25431138

[19] Ferrante C, Menkveld-Gfeller U, Cavin L. The first Jurassic coelacanth from Switzerland. Swiss Journal of Palaeontology. 2022;141: 1–20. 36164559 10.1186/s13358-022-00257-z PMC9499918

[20] Cavin L, Cupello C, Yabumoto Y, Fragoso L, Deesri UAB, P.M. Phylogeny and evolutionary history of mawsoniid coelacanths. Bulletin of the Kitakyushu Museum of Natural History and Human History, Series A (Natural History). 2019; 3–13.

[21] Dutel H, Maisey JG, Schwimmer DR, Janvier P, Herbin M, Clément G, et al. The giant Cretaceous coelacanth. and its bearing on Latimerioidei interrelationships PLoS One. 2012;7: 49911. 10.1371/journal.pone.0049911 PMC3507921 23209614

[22] Cavin L, Toriño P, Vranken N, Carter B, Polcyn MJ, Winkler D. The first late cretaceous mawsoniid coelacanth (Sarcopterygii: Actinistia) from North America: Evidence of a lineage of extinct ‘living fossils.’ Plos one. 2021;16: 0259292. 10.1371/journal.pone.0259292 PMC8584698 34762682

[23] Toriño P, Dutel H, Soto M, Norbis W, Ezquerra V, Perea D. Reconstructing an ancient fish: Three‐dimensional skeletal restoration of the head of Mawsonia (Sarcopterygii, Actinistia) using CT scan, and an adjusted model for body size estimation in fossil coelacanths. Journal of Anatomy. 2024. doi: 10.1111/joa.14054 38749764 PMC11306766

[24] Dutel H, Herbin M, Clément G. First occurrence of a mawsoniid coelacanth in the Early Jurassic of Europe. Journal of Vertebrate Paleontology. 2015;35: 929581.

[25] Carvalho MS, Maisey JG. New occurrence of Mawsonia (Sarcopterygii: Actinistia) from the Early Cretaceous of the Sanfranciscana Basin. Minas Gerais, southeastern Brazil Geological Society, London, Special Publications. 2008;295: 109–144.

[26] Soto M, Carvalho MS, Maisey JG, Perea D, Silva JD. Coelacanth remains from the Late Jurassic–? earliest Cretaceous of Uruguay: the southernmost occurrence of the Mawsoniidae. Journal of Vertebrate Paleontology. 2012;32: 530–537.

[27] Fragoso LGC, Brito P, Yabumoto Y. Axelrodichthys araripensis Maisey, 1986 revisited. Historical Biology. 2018.

[28] Yabumoto Y. A new coelacanth from the Early Cretaceous of Brazil (Sarcopterygii, Actinistia. Paleontological Research. 2002;6: 343–350.

[29] Yabumoto Y. A new Mesozoic coelacanth from Brazil (Sarcopterygii, Actinistia. Paleontological Research. 2008;12: 329–343.

[30] Cupello C, Batista TA, Fragoso LG, Brito PM. Mawsoniid remains (Sarcopterygii: Actinistia) from the lacustrine Missão Velha formation (lower cretaceous) of the Araripe Basin, North-east Brazil. Cretaceous Research. 2016;65: 10–16.

[31] Cavin L, Tong H, Buffetaut E, Wongko K, Suteethorn V, Deesri U. The First Fossil Coelacanth from Thailand. Diversity. 2023;15: 286.

[32] Cavin L, Forey PL, Buffetaut E, Tong H. Latest European coelacanth shows Gondwanan affinities. Biology Letters. 2005;1: 176–177. doi: 10.1098/rsbl.2004.0287 17148159 PMC1626220

[33] Cavin L, Valentin X, Garcia G. A new mawsoniid coelacanth (Actinistia) from the Upper Cretaceous of Southern France. Cretaceous Research. 2016;62: 65–73.

[34] Cavin L, Buffetaut E, Dutour Y, Garcia G, Le Loeuff J, Mechin A, et al. The last known freshwater coelacanths: New Late Cretaceous mawsoniid remains (Osteichthyes: Actinistia) from southern France. Plos one. 2020;15: 0234183. doi: 10.1371/journal.pone.0234183 32502171 PMC7274394

[35] Deesri U, Cavin L, Amiot R, Bardet N, Buffetaut E, Cuny G, et al. A mawsoniid coelacanth (Sarcopterygii: Actinistia) from the Rhaetian (Upper Triassic) of the Peygros quarry, Le Thoronet (Var, southeastern France. Geological Magazine. 2018;155: 187–192.

[36] Hartung J, Sander PM, Friedman M, Wintrich T. First record of mawsoniid coelacanths (Actinistia, Sarcopterygii) from the marine Rhaetian (Upper Triassic) of Bonenburg. Germany Journal of Vertebrate Paleontology. 2021;41: 1931258.

[37] Schaeffer B. Late Triassic fishes from the western United States. Bulletin of the AMNH. 1967;v. 135: 6.

[38] Elliott DK. A new specimen of Chinlea sorenseni from the Chinle Formation, Dolores River, Colorado. Journal of the Arizona-Nevada Academy of Science. 1987; 47–52.

[39] Schaeffer B. A study of Diplurus longicaudatus with notes on the body form and locomotion of the Coelacanthini. American Museum novitates. 1948.

[40] Schaeffer B. The Triassic coelacanth fish Diplurus, with observations on the evolution of the Coelacanthini. Bulletin of the American Museum of Natural History. 1952;99: 27.

[41] Millot J, Anthony J. Anatomie de Latimeria chalumnae. Tome I. Squelette et Muscles et formations de soutien. Paris: Centre National de la Recherche Scientifique; 1958.

[42] Nelson GJ. Gill arches and the phylogeny of fishes, with notes on the classification of vertebrates. Bulletin of the American Museum of Natural History. 1969;141.

[43] Brito PM, Meunier FJ, Clement G, Geffard‐Kuriyama D. The histological structure of the calcified lung of the fossil coelacanth Axelrodichthys araripensis (Actinistia: Mawsoniidae. Palaeontology. 2010;53: 1281–1290.

[44] Cupello C, Meunier FJ, Herbin M, Clément G, Brito PM. Lung anatomy and histology of the extant coelacanth shed light on the loss of air-breathing during deep-water adaptation in actinistians. Royal Society Open Science. 2017;4: 161030. doi: 10.1098/rsos.161030 28405393 PMC5383850

[45] Cupello C, Clément G, Brito PM. Evolution of air breathing and lung distribution among fossil fishes. Evolution and Development of Fishes. Cambridge: Cambridge University Press; 2019.

[46] Forey PL. Latimeria chalumnae and its pedigree. The biology of Latimeria chalumnae and evolution of coelacanths. 1991. pp. 75–98.

[47] Arratia G, Schultze HP. A new fossil actinistian from the Early Jurassic of Chile and its bearing on the phylogeny of Actinistia. Journal of Vertebrate Paleontology. 2015;35: 983524.

[48] Forey PL. The coelacanth Rhabdoderma in the Carboniferous of the British Isles. Palaeontology. 1981;24: 203–229.

[49] Clément G. A new coelacanth (Actinistia, Sarcopterygii) from the Jurassic of France, and the question of the closest relative fossil to Latimeria. Journal of Vertebrate Paleontology. 2005;25: 481–491.

[50] Johanson Z, Long JA, Talent JA, Janvier P, Warren JW. Oldest coelacanth, from the Early Devonian of Australia. Biology letters. 2006;2: 443–446. doi: 10.1098/rsbl.2006.0470 17148426 PMC1686207

[51] Cavin L, Grădinaru E. Dobrogeria aegyssensis, a new early Spathian (Early Triassic) coelacanth from north Dobrogea (Romania. Acta Geologica Polonica. 2014;64: 161–187.

[52] Maisey JG. Coelacanths from the lower cretaceous of Brazil. American Museum of Natural History; 1986.

[53] Toriño P, Soto M, Perea D, Carvalho MSS. New findings of the coelacanth Mawsonia Woodward (Actinistia, Latimerioidei) from the Late Jurassic–Early Cretaceous of Uruguay: Novel anatomical and taxonomic considerations and an emended diagnosis for the genus. Journal of South American Earth Sciences. 2021;107: 103054.

[54] Stensiö EA. Triassic Fishes from East Greenland. Reitzel CA, editor. 1932.

[55] Mansuit R, Clément G, Herrel A, Dutel H, Tafforeau P, Santin MD, et al. Development and growth of the pectoral girdle and fin skeleton in the extant coelacanth Latimeria chalumnae. Journal of Anatomy. 2020;236: 493–509. doi: 10.1111/joa.13115 31713843 PMC7018633

[56] Andrews SM. Interrelationships of crossopterygians. In: Greenwood PH, Miles RS, Patterson C, editors. Interrelationships of Fishes. London: Academic Press; 1973. pp. 137–177,.

[57] Gardiner BG. The relationships of the palaeoniscid fishes, a review based on new specimens of Mimia and Moythomasia from the Upper Devonian of Western Australia. Bulletin of the British Museum (Natural History), Geology Series. 1984;37: 173–428.

[58] Friedman M, Coates MI, Anderson P. First discovery of a primitive coelacanth fin fills a major gap in the evolution of lobed fins and limbs. Evolution & Development. 2007;9: 329–337. 10.1111/j.1525-142X.2007.00169.x 17651357

[59] Schultze HP. Coelacanth fish (Actinista, Sarcopterygii) From the Late Pennsylvanian of the Kinney Brick Company Quarry. New Mexico: New Mexico Bureau Mines and Mineral Resources Bulletin. 1992;138: 205–209.

[60] Beltan L. A propos de l’ichthyofaune triasique de la Catalogne espagnole. Colloques Internationaux du Centre National de la Recherque Scientifique. 1975;218: 273–280.

[61] Jessen H. Weitere fischreste aus dem Oberen Plattenkalk der Bergisch-Gladbach Paffrather Mulde Oberdevon Rheinisches Schiefergebirge. Palaeontographica A. 1973;143: 159–187.

[62] Stensiö EA. On the Devonian coelacanthids of Germany with special reference to the dermal skeleton. Kungliga Svenska Vetenskapsakademiens Handlingar. 1937;16: 1–56.

[63] Gess RW, Coates MI. Fossil juvenile coelacanths from the Devonian of South Africa shed light on the order of character acquisition in actinistians. Zoological Journal of the Linnean Society. 2015;175: 360–383.

[64] Mondéjar‐Fernández J, Meunier FJ, Cloutier R, Clément G, Laurin M. A microanatomical and histological study of the scales of the Devonian sarcopterygian Miguashaia bureaui and the evolution of the squamation in coelacanths. Journal of Anatomy. 2021;239: 451–478. doi: 10.1111/joa.13428 33748974 PMC8273612

[65] Renesto S, Stockar R. First record of a coelacanth fish from the Middle Triassic Meride Limestone of Monte San giorgio (canton ticino, Switzerland. Rivista Italiana di Paleontologia e Stratigrafia. 2018;124.

[66] Woodward AS. I.—On some Permo-Carboniferous fishes from Madagascar. Annals and Magazine of Natural History. 1910;5: 1–6.

[67] Moy-Thomas JA. The coelacanth fishes from Madagascar. Geological Magazine. 1935;72: 213–227.

[68] Schaumberg G. Neubeschreibung von Coelacanthus granulatus Agassiz (Actinistia, Pisces) aus dem Kupferschiefer von Richelsdorf (Perm, W.-Deutschland. Paläontologische Zeitschrift. 1978;52: 169–197.

[69] Woodward AS. A synopsis of the vertebrate fossils of the English Chalk. Proceedings of the Geologists’ Association. 1888;10: 273–338.

[70] Lambers PH. A redescription of the coelacanth Macropoma willemoesii Vetter from the lithographic limestone of Solnhofen (Upper Jurassic, Bavaria. Mesozoic Fishes—Systematics and Paleoecology. 1996. pp. 395–407.

[71] Reis OM. Die Coelacanthinen mit besonderer Berücksichtigung der im Weissen Jura Bayerns vorkommenden Arten. Palaeontographica. 1888; 1–96.

[72] Woodward AS. The Fossil Fishes of the English Chalk. Part V, Monographs of the Palaeontographical Society. 1909;63: 153–184.

[73] Lambers P. On the ichthyofauna of the Solnhofen lithographic limestone (Upper Jurassic. Germany; 1992.

[74] Clément G. The actinistian (Sarcopterygii) Piveteauia madagascariensis Lehman from the Lower Triassic of northwestern Madagascar: a redescription on the basis of new material. Journal of Vertebrate Paleontology. 1999;19: 234–242.

[75] Meunier FJ, Cupello C, Herbin M, Clément G, Brito PM. The lungs of extinct and extant coelacanths: a morphological and histological review. Cybium: Revue Internationale d’Ichtyologie. 2021.

[76] Wang N, Liu HT. Coelacanth fishes from the marine Permian of Zhejiang, South China. Vertebrata Palasiatica. 1981;19: 305.

[77] Dutel H, Galland M, Tafforeau P, Long JA, Fagan MJ, Janvier P, et al. Neurocranial development of the coelacanth and the evolution of the sarcopterygian head. Nature. 2019;569: 556–559. doi: 10.1038/s41586-019-1117-3 30996349

[78] Nulens R, Scott L, Herbin M. An updated inventory of all known specimens of the coelacanth, Latimeria spp. South African Institute for Aquatic Biodiversity. 2011.

[79] Yabumoto Y, Brito PM. The second record of a mawsoniid coelacanth from the Lower Cretaceous Crato Formation, Araripe Basin, northeasthern Brazil, with comments on the development of coelacanths. Mesozoic fishes. 2013;5: 489–497.

[80] Hensel K. Morphologie et interprétation des canaux et canalicules sensoriels céphaliques de Latimeria chalumnae Smith, 1939 (Osteichthyes, Crossopterygii, Coelacanthiformes. Bulletin du Muséum national d’histoire naturelle Section A, Zoologie, biologie et écologie animales. 1986;8: 379–407.

[81] Berra TM. Some 20th century fish discoveries. Environmental Biology of Fishes. 1997;50: 1–12.

[82] Cau A, Beyrand V, Voeten DF, Fernandez V, Tafforeau P, Stein K, et al. Synchrotron scanning reveals amphibious ecomorphology in a new clade of bird-like dinosaurs. Nature. 2017;552: 395–399. doi: 10.1038/nature24679 29211712

[83] Carlson KJ, Stout D, Jashashvili T, Ruiter DJ, Tafforeau P, Carlson K, et al. The endocast of MH1, Australopithecus sediba. Science. 2011;333: 1402–1407. doi: 10.1126/science.1203922 21903804

[84] Mirone A, Brun E, Gouillart E, Tafforeau P, Kieffer J. The PyHST2 hybrid distributed code for high speed tomographic reconstruction with iterative reconstruction and a priori knowledge capabilities. Nuclear Instruments and Methods in Physics Research Section B: Beam Interactions with Materials and Atoms. 2014;324: 41–48.

[85] Paganin D, Mayo SC, Gureyev TE, Miller PR, Wilkins SW. Simultaneous phase and amplitude extraction from a single defocused image of a homogeneous object. Journal of microscopy. 2002;206: 33–40. doi: 10.1046/j.1365-2818.2002.01010.x 12000561

[86] Manuelli L, Covain R, Cavin L. A 3D reconstruction of the skull of the West Indian Ocean coelacanth Latimeria chalumnae. M3. 2023;9: e211.

[87] Hensel K, Balon EK. The sensory canal systems of the living coelacanth, Latimeria chalumnae: A new instalment. Environmental Biology of Fishes. 2001;61: 117–124.

[88] Swofford DL, Sullivan J. Phylogeny inference based on parsimony and other methods using PAUP*. The phylogenetic handbook: a practical approach to DNA and protein phylogeny, cáp. 2003. pp. 160–206.

[89] Müller KF. The efficiency of different search strategies in estimating parsimony jackknife, bootstrap, and Bremer support. BMC Evolutionary Biology. 2005;5: 1–10. 16255783 10.1186/1471-2148-5-58 PMC1282575

