# Supplementary material for: The most detailed anatomical reconstruction of a Mesozoic coelacanth
Source: PLoS One. 2024 Nov 6;19(11):e0312026. doi: 10.1371/journal.pone.0312026 (PMC11540180; doi:10.1371/journal.pone.0312026)
Supplement: S1 Appendix — The character numbers correspond to the ones of Ferrante and Cavin [7] unless stated otherwise. (DOCX) [file pone.0312026.s002.docx]

**Dermal bones of the skull roof:**

Character 1

Parietonasal and postparietal shields

0. free from one to another

1. sutured to each other

Character 2

Parietonasal versus postparietal shields

0. smaller or almost of the same length

1. longer

Character 3

Snout bones

0. lying free from one another

1. consolidated

Character 4

Premaxillary teeth

0. equal or more than 5

1. equal or less than 4

Character 5

Premaxilla

0. with dorsal lamina

1. without dorsal lamina

Character 6

Anterior opening of the rostral organ

0. contained within premaxilla

1. within separated rostral ossicles

Character 7

Internasal

0. several

1. one or none

Character 8

Parietal

0. one pair

1. two pairs

Character 9

Anterior and posterior pairs of parietals

0. of similar size

1. of dissimilar size

Character 10

Parietals and postparietals

0. without raised areas

1. with raised areas

Character 11

Parietal descending process

0. absent or highly reduced to a ridge

1. present

Character 12

Number of supraorbitals and tectals

0. equal or less than 9

1. equal or more than 10

Character 13

Preorbital

0. absent

1. present

Character 14

Intertemporal

0. absent

1. present

Character 15

Postparietal descending process

0. absent or highly reduced to a ridge

1. present

Character 16

Supratemporal descending process

0. absent or highly reduced to a ridge

1. present

Character 17

Posterior margin of the skull roof

0. straight

1. embayed

Character 18

Extrascapulars

0. sutured with postparietals

1. free

Character 19

Extrascapulars

0. behind level of neurocranium

1. forming part of the skull roof

Character 20

Pair(s) of lateral extrascapulars (without the triple junction for sensory canals)

0. none

1. one

2. two or more

Character 21

Median extrascapular

0. present

1. absent

Character 22

Supraorbital sensory canal

0. running through centre of ossification

1. following sutural course

Character 23

Supraorbital sensory canals opening as

0. few pores at the sutural contact of bones

1. bifurcating pores

2. many pores within bones

3. continuous groove crossed by pillars

4. continuous groove without pillars

Character 24

Medial branch of otic canal

0. absent

1. present

Character 25

Anterior branches of supratemporal commissure

0. absent

1. present

Character 26

Pit lines

0. marking postparietals

1. not marking postparietals

Character 27

Middle and posterior pit lines

0. within posterior half of postparietals

1. within anterior third

Character 28

Dermal bones of the skull roof ornamented with

0. coarse and/or irregularly shaped tubercles and/or elongated continuous/discontinuous vermiform/linear ridged tuberculation

1. round tubercles

2. coarse rugosities and fine to pronounced striae

3. mostly or entirely unornamented

**Cheek bones and sensory canals:**

Character 29

Cheek bones

0. sutured to one another

1. separated from one another

Character 30

Spiracular (postspiracular)

0. absent

1. present

Character 31

Postorbital

0. simple, without anterodorsal

excavation

1. anterodorsal excavation in the postorbital

Character 32

Postorbital

0. without anterior process

1. with anterior process

Character 33

Postorbital

0. large

1. reduced to a narrow tube surrounding the sensory canal only

Character 34

Postorbital

0. entirely behind the level of the intracranial joint

1. spanning the intracranial joint

Character 35

Jugal

0. present

1. absent

Character 36

Squamosal

0. large

1. reduced to a narrow tube surrounding the jugal sensory canal only

Character 37

Squamosal

0. limited to the mid-level of cheek

1. extending behind the postorbital to reach the skull roof

Character 38

Preopercle

0. large

1. reduced to a narrow tube surrounding the preopercular canal

Character 39

Preopercle

0. undifferentiated

1. developed as a posterior tube-like canal-bearing portion and an anterior blade-like portion

Character 40

Position of the preopercle within the cheek

0. posterior to the squamosal and/or the postorbital

1. below or anterior to the squamosal and the postorbital

Character 41

Subopercle

0. absent

1. present

Character 42

Anterior end of the lachrymojugal

0. simple

1. angled and/or expanded

Character 43

Lachrymojugal

0. with parallel margins along its entire lenght

1. with a more or less thick triangular portion

Character 44

Contact between the lachrymojugal and the preorbital or tectal-supraorbital series

0. present

1. absent

Character 45

Posterior nostril on the lachrymojugal

0. not marked

1. marked

Character 46

Posterior opening of the rostral organ marks

0. the preorbital

1. the lachrymojugal

2. the tectal and/or no bones

Character 47

Posterior opening(s) of the rostral organ mark(s) bone as

0. foramen(s)

1. notch(es) or groove(s)

2. not marking bone

Character 48

Anterior and/or posterior branches of the infraorbital canal within the postorbital

0. absent (canal simple)

1. present

Character 49

Infraorbital sensory canal

0. running through centre of postorbital

1. running at the anterior margin of the postorbital

Character 50

Prominent branches of the jugal sensory canal within the squamosal

0. absent (canal simple)

1. present

Character 51

Jugal sensory canal

0. running through centre of bone

1. running along the ventral margin of the squamosal

Character 52

Infraorbital, jugal and preopercular sensory canals

0. opening through many tiny pores

1. opening through a few large pores

2. opening as a large, continuous groove crossed by pillars

Character 53

Pit lines

0. marking cheek bones

1. failing to mark cheek bones

Character 54

Dermal bones of the cheek ornamented with

0. coarse and/or irregularly shaped tubercles and/or elongated continuous/discontinuous vermiform/linear ridged tuberculation

1. round tubercles

2. coarse rugosities and fine to pronounced striae

3. mostly or entirely unornamented

Character 55

Orbital space

0. small and occupied entirely by the eye

1. large and not entirely occupied by the eye

Character 56

Sclerotic ossicles

0. absent

1. present

**Lower jaw:**

Character 57

Retroarticular and articular

0. co-ossified

1. separated

Character 58

Dentary

0. simple

1. dentary hook-shaped

Character 59

Dentary

0. without prominent lateral swelling

1. with swelling

Character 60

Dentary

0. with fused dentary teeth

1. with separated dentary teeth or edentulous

Character 61

Principal coronoid

0. lying free

1. sutured to angular

Character 62

Number of anterior coronoids

0. four or more

1. three or less

Character 63

Coronoid

0. opposite to the posterior end of dentary not modified

1. modified

Character 64

Coronoid fangs

0. absent

1. present

Character 65

Prearticular and/or coronoid teeth

0. pointed and smooth

1. rounded and marked with fine striations radiating from the crown

Character 66

Dentary sensory pore [character 67 from Ferrante and Cavin (7)]

0. absent

1. present

Character 67

Mandibular sensory canal on the splenial [character 68 from Ferrante and Cavin (7)]

0. opening through laterally directed pores

1. opening through ventrally directed pores

Character 68

Oral pit line [Ferrante and Cavin (7): character 69]

0. marking the angular

1. not marking the angular

Character 69

Oral pit line [character 70 from Ferrante and Cavin (7)]

0. confined to angular

1. oral pit line reaching forward to the dentary and/or the splenial

**Neurocranium, parasphenoid and vomer:**

Character 70

Orbitosphenoid and basisphenoid regions [character 71 from Ferrante and Cavin (7)]

0. co-ossified

1. separate

Character 71

Processus connectens [character 72 from Ferrante and Cavin (7)]

0. failing to meet parasphenoid

1. meeting parasphenoid

Character 72

Basipterygoid process [character 73 from Ferrante and Cavin (7)]

0. absent

1. present

Character 73

Temporal excavation [character 74 from Ferrante and Cavin (7)]

0. not lined with bone

1. lined with bone

Character 74

Otico-occipital [character 75 from Ferrante and Cavin (7)]

0. solid

1. separated to prootic/opisthotic

Character 75

Supraoccipital [character 76 from Ferrante and Cavin (7)]

0. absent

1. present

Character 76

Toothed area of the parasphenoid [character 77 from Ferrante and Cavin (7)]

0. covers most of the ventral surface

1. restricted to the anterior half

Character 77

Buccohypophysial canal [character 78 from Ferrante and Cavin (7)]

0. closed

1. opening through parasphenoid

Character 78

Parasphenoid [character 79 from Ferrante and Cavin (7)]

0. without ascending laminae anteriorly

1. with ascending laminae

Character 79

Suprapterygoid process [character 80 from Ferrante and Cavin (7)]

0. absent

1. present

Character 80

Vomers [character 81 from Ferrante and Cavin (7)]

0. not meeting in the midline

1. meeting medially

Character 81

Prootic [character 82 from Ferrante and Cavin (7)]

0. without complex suture with the basioccipital

1. with a complex suture

Character 82

Superficial ophthalmic branch of anterodorsal lateral line nerve [character 83 from Ferrante and Cavin (7)]

0. not piercing antotic process

1. piercing antotic process

Character 83

Process on braincase for articulation of infrapharyngobranchial 1 [character 84 from Ferrante and Cavin (7)]

0. absent

1. present

Character 84

Separate lateral ethmoids [character 85 from Ferrante and Cavin (7)]

0. absent

1. present

Character 85

Separate basioccipital [character 86 from Ferrante and Cavin (7)]

0. absent

1. present

Character 86

Dorsum sellae [character 87 from Ferrante and Cavin (7)]

0. small

1. large and constricting entrance to cranial cavity anterior to the intracranial joint

**Palate, Hyoid and gill arches:**

Character 87

Ventral swelling of the palatoquadrate [character 88 from Ferrante and Cavin (7)]

0. absent

1. present

Character 88

Basibranchial tooth plates [character 89 from Ferrante and Cavin (7)]

0. three median pairs or more

1. two median pairs or less

Character 89

Anterior basibranchial tooth plates [character 90 from Ferrante and Cavin (7)]

0. paired

1. fused

**Postcranial skeleton:**

Character 90

Extracleithrum [character 91 from Ferrante and Cavin (7)]

0. absent

1. present

Character 91

Anocleithrum [character 92 from Ferrante and Cavin (7)]

0. simple

1. forked

Character 92

Number of neural arches [character 93 from Ferrante and Cavin (7)]

0. equal or more than 50

1. equal or less than 49

Character 93

Posterior neural and haemal spines [character 94 from Ferrante and Cavin (7)]

0. abutting one another

1. not abutting

Character 94

Occipital neural arches [character 95 from Ferrante and Cavin (7)]

0. not expanded

1. expanded

Character 95

Ossified ribs [character 96 from Ferrante and Cavin (7)]

0. absent

1. present

Character 96

Ossified lung [character 97 from Ferrante and Cavin (7)]

0. absent

1. present

Character 97

Basal plate of the anterior dorsal fin [character 98 from Ferrante and Cavin (7)]

0. with smooth ventral margin

1. emarginated and accommodating the tips of adjacent neural spines

Character 98

Fin rays in the anterior dorsal fin [character 99 from Ferrante and Cavin (7)]

0. more or equal to 11

1. less than or equal as 10

Character 99

Anterior dorsal fin [character 100 from Ferrante and Cavin (7)]

0. without denticles

1. with denticles

Character 100

Basal support of the second dorsal fin [character 101 from Ferrante and Cavin (7)]

0. simple

1. forked anteriorly

Character 101

Pelvics [character 102 from Ferrante and Cavin (7)]

0. abdominal

1. thoracic

Character 102

Pelvic bones of each side [character 103 from Ferrante and Cavin (7)]

0. remain separate

1. fused in midline

Character 103

Diphycercal tail [character 104 from Ferrante and Cavin (7)]

0. absent

1. present

Character 104

Caudal lobes [character 105 from Ferrante and Cavin (7)]

0. symmetrical

1. asymmetrical

Character 105

Fin rays [character 106 from Ferrante and Cavin (7)]

0. more numerous than radials

1. equal in number

Character 106

Fin ray [character 107 from Ferrante and Cavin (7)]

0. branched

1. unbranched

Character 107

Paired fin rays [character 108 from Ferrante and Cavin (7)]

0. slender

1. expanded

Character 108

Median fin rays [character 109 from Ferrante and Cavin (7)]

0. slender

1. expanded

Character 109

Lateral line openings in scales [character 110 from Ferrante and Cavin (7)]

0. single

1. multiple

Character 110

Ventral keel scales [character 111 from Ferrante and Cavin (7)]

0. absent

1. present

Character 111

Scale ornament [character 112 from Ferrante and Cavin (7)]

0. not differentiated

1. differentiated
